# Supplementary material for: Approaching a fully-polarized state of nuclear spins in a solid
Source: Nat Commun. 2024 Feb 2;15:985. doi: 10.1038/s41467-024-45364-2 (PMC10837425; doi:10.1038/s41467-024-45364-2)
Supplement: Supplementary file 1 — Supplementary Information [file 41467_2024_45364_MOESM1_ESM.pdf]

Supplemental Information: Approaching a fully-polarized state of nuclear spins in a solid

Peter Millington-Hotze, Harry E. Dyte, Santanu Manna, Saimon F. Covre da Silva, Armando Rastelli, and Evgeny A. Chekhovich

CONTENTS

Supplementary Note 1. Sample structure 2

Supplementary Note 2. Electron-nuclear spin system of a quantum dot 3

Supplementary Note 3. Experimental methods and techniques 9
- A. Pump probe experiment timing 9
- B. Optical pumping of quantum dot nuclear spins 12
- C. Optical probing of quantum dot nuclear spins 16
- D. Radiofrequency control of nuclear spins 17

Supplementary Note 4. Additional experimental data and discussion 19
- A. Extended data from nuclear spin pumping measurements 19
- B. Estimate of the electron tunneling rate 26
- C. Nuclear spin buildup dynamics 29
- D. Relation between nuclear spin cooling and state narrowing 32

Supplementary Note 5. Derivation of nuclear spin polarization 35
- A. Nuclear magnetic resonance thermometry of spin-3/2 nuclei 35
- B. Corrections for the nuclei with small or inverted quadrupolar shifts 38
- C. NMR spectra of the QD nuclei 42
- D. Calibration of the adiabatic radiofrequency sweeps 46
- E. Model fitting for derivation of the nuclear spin polarization degree 49
- F. Error analysis in model fitting of the nuclear spin polarization data 53

Supplementary Note 6. Raw data 57

References 59

Supplementary Note 1. SAMPLE STRUCTURE

The sample is grown using molecular beam epitaxy (MBE) on a semi-insulating GaAs (001) substrate. The layer sequence of the semiconductor structure is shown in Supplementary Fig. 1. The growth starts with a layer of $Al_{0.95}Ga_{0.05}As$ followed by a single pair of $Al_{0.2}Ga_{0.8}As$ and $Al_{0.95}Ga_{0.05}As$ layers acting as a Bragg reflector in optical experiments. Then, a 95 nm thick layer of $Al_{0.15}Ga_{0.85}As$ is grown, followed by a 95 nm thick layer of $Al_{0.15}Ga_{0.85}As$ doped with Si at a volume concentration of 1.0×10^{18} cm^{-3}. The low Al concentration of 0.15 in the Si doped layer mitigates the issues caused by the deep DX centers [1–3]. The n-type doped layer is followed by the electron tunnel barrier layers: first a 5 nm thick $Al_{0.15}Ga_{0.85}As$ layer is grown at a reduced temperature of 560 °C to suppress Si segregation, followed by a 10 nm thick $Al_{0.15}Ga_{0.85}As$ and then a 15 nm thick $Al_{0.33}Ga_{0.67}As$ layer grown at 600 °C. Aluminium droplets are grown on the surface of the $Al_{0.33}Ga_{0.67}As$ layer and are used to etch the nanoholes [4, 5]. Atomic force microscopy shows that typical nanoholes have a depth of $\approx$ 6.5 nm and are $\approx$ 70 nm in diameter. Next, a 2.1 nm thick layer of GaAs is grown to form QDs by infilling the nanoholes as well as to form the quantum well (QW) layer. Thus, the maximum height of the QDs in the growth z direction is $\approx$ 9 nm. The GaAs layer is followed by a 268 nm thick $Al_{0.33}Ga_{0.67}As$ barrier layer. Finally, the p-type contact layers doped with C are grown: a 65 nm thick layer of $Al_{0.15}Ga_{0.85}As$ with a 5×10^{18} cm^{-3} doping concentration, followed by a 5 nm thick layer of $Al_{0.15}Ga_{0.85}As$ with a 9×10^{18} cm^{-3} concentration, and a 10 nm thick layer of GaAs with a 9×10^{18} cm^{-3} concentration.

The sample is processed into a $p-i-n$ diode structure. Mesa structures with a height of 250 nm are formed by etching away the p-doped layers and depositing Ni(10 nm)/AuGe(150 nm)/Ni(40 nm)/Au(100 nm) on the etched areas. The sample is then annealed to enable diffusion down to the n-doped layer to form the ohmic back contact. The top gate contact is formed by depositing Ti(15 nm)/Au(100 nm) on to the p-type surface of the mesa areas. Quantum dot photoluminescence (PL) is excited and collected through the top of the sample. The sample gate bias V_{Gate} is the bias of the p-type top contact with respect to the grounded n-type back contact. Due to the large thickness of the top $Al_{0.33}Ga_{0.67}As$ layer, the tunneling of the holes is suppressed, whereas tunnel coupling to the n-type layer enables deterministic charging of the quantum dots with electrons by changing V_{Gate}.

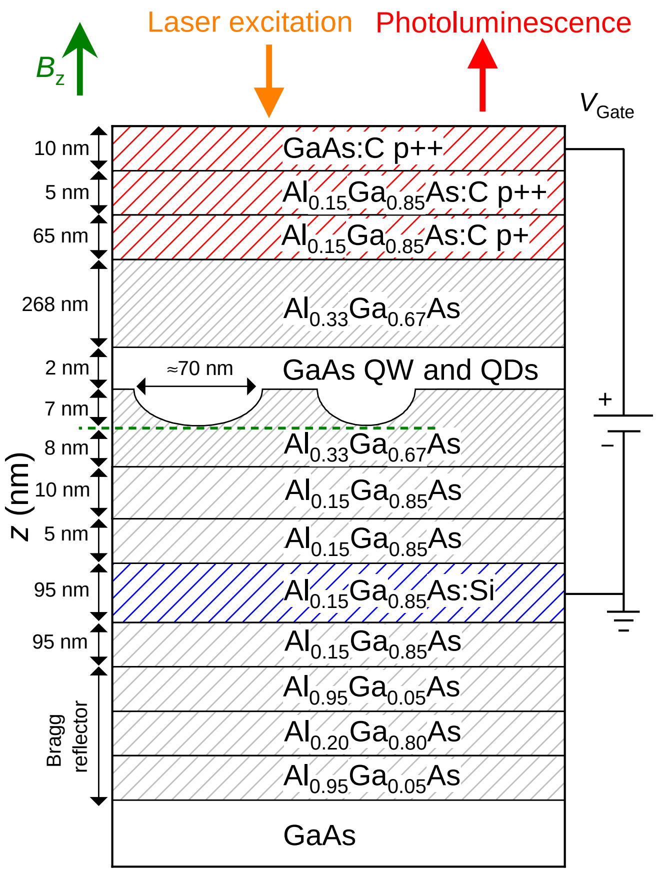

Supplementary Figure 1. **Quantum dot sample structure used in this work.**

Supplementary Note 2. ELECTRON-NUCLEAR SPIN SYSTEM OF A QUANTUM DOT

The band structure of the electrons and holes in a GaAs QD is sketched in Supplementary Fig. 2 (see for example Ref. [6] for a review). The electron conduction band in GaAs has spin $s = 1/2$, with two possible spin projections $s_z = \pm 1/2$ along the quantizing magnetic field. The valence band is four-fold degenerate at the center of the Brillouin zone in bulk GaAs. The confinement along the z growth axis is sufficient to split the valence band into the heavy hole and light hole subbands with total momentum projections $j_z = \pm 3/2$ and $j_z = \pm 1/2$, respectively. The typical heavy-light hole splitting is $\Delta E_{\mathrm{hh-lh}} \approx 10 - 15$ meV in GaAs/AlGaAs quantum wells, depending on their thickness [7, 8]. The selection rules for the ground state heavy-hole excitons are such that σ^+ (σ^-) circularly polarized light couples only to the $s_z = -1/2$ ($s_z = +1/2$) electron state in the conduction band. For the light hole excitons the selection rules are inverted. This means that high-fidelity initialization of the electron spin via circularly-polarized optical pumping is only

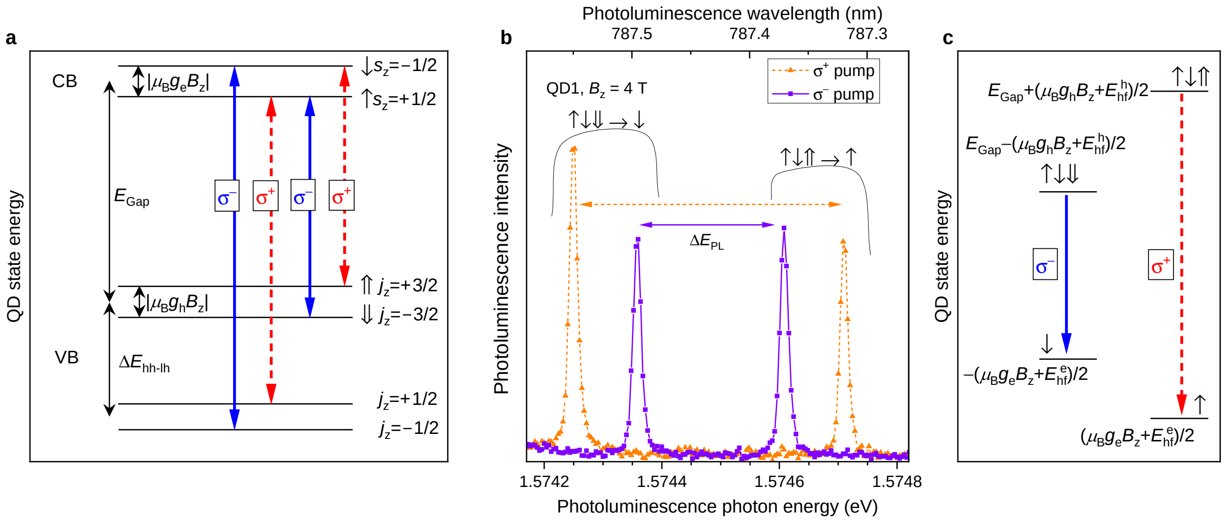

Supplementary Figure 2. **Optical transitions in a GaAs quantum dot.** **a,** Electron energy levels in the conduction band (CB) and hole energy levels in the valence band (VB). Electron states with spin up (↑) and down (↓) have spin projections $s_z = +1/2$ and $s_z = -1/2$, respectively. The heavy hole states with pseudospin up (⇑) and down (⇓) have momentum projections $j_z = +3/2$ and $j_z = -3/2$, respectively. The light hole states have momentum projections $j_z = \pm 1/2$. Dashed (solid) arrows show σ^+ (σ^-) polarized optical transitions. **b,** Typical photoluminescence spectra of an individual QD. The spectral splitting $\Delta E_{\rm PL}$ depends on the helicity of the optical pumping ($\sigma^\pm$) due to the buildup of the nuclear spin polarization. **c,** Energy level diagram of a negatively charged trion in a GaAs QD. The electron ground state is split by the Zeeman energy $\mu_{\rm B} g_{\rm e} B_{\rm z}$ and the hyperfine shift $E^{\rm e}_{\rm hf}$. The X^- trion energy includes the QD bandgap energy $E_{\rm Gap}$, the Zeeman splitting and a small [9] hyperfine shift $E^{\rm h}_{\rm hf}$. The electron and hole g-factors are $g_{\rm e}$ and $g_{\rm h}$, respectively, with $|g_{\rm h}| \gg |g_{\rm e}|$ in the studied QDs. Arrows depict the two optically allowed transitions responsible for the spectral doublet in (b). Note that the g-factors shown in (a) do not necessarily coincide with those shown in (c), since the states used for excitation and detection are in general different.

possible for a sufficiently large spectral separation $\Delta E_{\rm hh-lh}$ of the heavy and light hole exciton transitions.

Apart from the quantum-well type of confinement along the z axis, carriers in a QD are also confined in the orthogonal xy plane. In a real semiconductor structure there is always some breaking of the symmetry in the xy plane. Such in-plane anisotropy can mix the heavy and light holes, so that the eigenstates are no longer described by pure $j_z = \pm 3/2$ or $j_z = \pm 1/2$ projections. As a result of such mixing the selection rules change, and the optical transitions in general become elliptically polarized.

In the pump-probe experiments we use photoluminescence of a negatively charged trion X^-, where the electron-hole recombination occurs in presence of another resident electron. The X^-

spectra, such as shown in Supplementary Fig. 2b, tend to have the narrowest linewidths and their Zeeman splittings are free from the non-linearity which is presented for neutral excitons X^0 due to the fine structure splitting. The energies of the states involved in X^- photoluminescence are shown in Supplementary Fig. 2c. The energy of the ground state resident electron is $(\mu_{\rm B} g_{\rm e} B_{\rm z} + E^{\rm e}_{\rm hf}) s_{\rm z}$, while the energy of the optically excited trion is $E_{\rm Gap} + (\mu_{\rm B} g_{\rm h} B_{\rm z} + E^{\rm h}_{\rm hf}) j_{\rm z}/3$, where $E_{\rm Gap}$ is the X^- transition energy in the absence of Zeeman and hyperfine shifts. Taking the differences and substituting the momentum projections allowed by the selection rules $s_{\rm z} + j_{\rm z} = \pm 1$, we find the photon energies of the two optically-allowed transitions $E_{\rm Gap} \pm \left(\mu_{\rm B}(g_{\rm h} - g_{\rm e}) B_{\rm z} + (E^{\rm h}_{\rm hf} - E^{\rm e}_{\rm hf})\right)/2$, where $g_{\rm e}$ ($g_{\rm h}$) is the electron (heavy hole) g-factor, $E^{\rm e}_{\rm hf}$ ($E^{\rm h}_{\rm hf}$) is the electron (heavy hole) hyperfine shift. The splitting of the spectral doublet is then

$$\Delta E_{\rm PL} = \mu_{\rm B}(g_{\rm h} - g_{\rm e}) B_{\rm z} + (E^{\rm h}_{\rm hf} - E^{\rm e}_{\rm hf}). \tag{S1}$$

Next we eliminate the Zeeman contribution and define the excitonic hyperfine shift:

$$E_{\rm hf} = -(\Delta E_{\rm PL} - \Delta E_{\rm PL,0}) = E^{\rm e}_{\rm hf} - E^{\rm h}_{\rm hf}, \tag{S2}$$

where $\Delta E_{\rm PL,0}$ is the photoluminescence doublet splitting at zero nuclear spin polarization. The valence band hole hyperfine interaction is of the order of 10% of the electron hyperfine interaction [9]. Consequently, the excitonic hyperfine shift $E_{\rm hf}$ is dominated by the electronic contribution $E^{\rm e}_{\rm hf}$.

The Hamiltonian describing the nuclear spin system alone includes the Zeeman, the quadrupolar and the dipole-dipole terms. The Zeeman term accounts for the coupling of the QD nuclear spins $\mathbf{I}_j$ to the static magnetic field $B_{\rm z}$ directed along the z axis:

$$\mathcal{H}_{\rm Z,N} = -\sum_{j=1}^{N} \hbar \gamma_j B_{\rm z} \hat{I}_{{\rm z},j}, \tag{S3}$$

where the summation goes over all individual nuclei $1 \leq j \leq N$, $\hbar = h/(2\pi)$ is the reduced Planck's constant, γ_j is the gyromagnetic ratio of the j-th nuclear spin and $\hat{\mathbf{I}}_j$ is a vector of spin operators with Cartesian components $(\hat{I}_{{\rm x},j}, \hat{I}_{{\rm y},j}, \hat{I}_{{\rm z},j})$. The result of the Zeeman term alone is a spectrum of equidistant single-spin eigenenergies $m\hbar\gamma_j B_{\rm z}$, corresponding to $2I+1$ eigenstates with $\hat{I}_{\rm z}$ projections m satisfying $-I \leq m \leq +I$.

The interaction of the nuclear electric quadrupolar moment with the electric field gradients is described by the term (Ch. 10 in Ref. [10]):

$$\mathcal{H}_{\rm Q,N} = \sum_{j=1}^{N} \frac{q_j}{6} [3\hat{I}^2_{{\rm z}',j} - I_j^2 + \eta_j(\hat{I}^2_{{\rm x}',j} - \hat{I}^2_{{\rm y}',j})], \tag{S4}$$

where q_j and η_j describe the magnitude and asymmetry of the electric field gradient tensor, whose principal axes are $x'y'z'$. The strain is inhomogeneous within the QD volume, so that q_j and η_j vary between the individual nuclei. The axes $x'y'z'$ are different for each nucleus and generally do not coincide with crystallographic axes or magnetic field direction. In lattice-matched GaAs/AlGaAs QDs the electric field gradients at the nuclear sites do not exceed $q_j/h \approx 200$ kHz, as witnessed via NMR spectroscopy. At sufficiently strong magnetic fields $|\hbar\gamma_j B_z| \gg |q_j|$, quadrupolar effects can be treated perturbatively – the main effect is the anharmonicity of the nuclear spin energies and the resulting quadrupolar NMR multiplet of $2I$ magnetic-dipole transitions, split by $\nu_Q \approx q_j/h$. The $m = \pm 1/2$ states of a half-integer nuclear spin are influenced by quadrupolar effects only in the second order, resulting in a smaller inhomogeneous broadening, which scales as $\propto \nu_Q^2/\nu_L$ with nuclear spin Larmor frequency $\nu_L = \gamma B_z/(2\pi)$.

Quadrupolar effects have a direct impact on nuclear spin cooling, which we now discuss briefly. According to the Hamiltonians of Supplementary Equations S3 and S4, the nuclear eigenstates have well-defined spin projections along the external magnetic field only under special conditions. For example, if the quadrupolar shifts are absent ($\nu_Q = 0$), or if the quadrupolar tensor has high symmetry ($\eta_j = 0$) with the major axis parallel to magnetic field ($z' \parallel z$), then the energy eigenstates are also the eigenstates of the nuclear spin z-projection operator $\hat{I}_{z,j}$, with eigenvalues $m = \pm 1/2, 3/2$ in case of spin-3/2 nuclei. But this is not the case for an arbitrary quadrupolar tensor. Thus, if a nuclear spin is initialized into a state with a definite m (for example through interaction with optically pumped electron spins), it will be a superposition state, which will evolve in time, decaying probabilistically (through decohering) into one of the energy eigenstates. In simple words, the nuclear spin eigenstates are generally not aligned with the external magnetic field. As a result, achieving complete nuclear spin polarization is strictly speaking impossible in the presence of low-symmetry quadrupolar interactions, as has been pointed out previously [11]. The scale of the effect can be characterized by considering the expectation value of the spin projection operator $\hat{I}_{z,j}$ for the true energy eigenstates. First-order expansion of the exact solution of the Hamiltonians of Supplementary Equations S3 and S4 shows that the deviation of the $\hat{I}_{z,j}$ expectation values from the ideal half-integer values scales as $\propto (\nu_Q/\nu_L)^2$. For GaAs QDs ($|\nu_Q| \lesssim 200$ kHz) in high magnetic field ($\nu_L \approx 100$ MHz) the deviation parameter is very small $(\nu_Q/\nu_L)^2 \approx 4 \times 10^{-6}$. The picture is very different with Stranski-Krastanov InGaAs QDs, where quadrupolar effects are large (up to $|\nu_Q| \lesssim 10$ MHz) and the distribution of the major quadrupolar axes is inhomogeneous [11, 12]. As a result, for a realistic range of magnetic fields, near-unity polarization of nuclear spins is fundamentally unachievable with InGaAs QDs [11].

Direct interaction between the nuclei is described by the dipole-dipole Hamiltonian:

$$\mathcal{H}_{\rm DD} = \sum_{1\leq j<k\leq N} b_{j,k}\left(3\hat{I}_{{\rm z},j}\hat{I}_{{\rm z},k} - \hat{\mathbf{I}}_j\cdot\hat{\mathbf{I}}_k\right),$$
$$b_{j,k} = \frac{\mu_0\hbar^2}{4\pi}\frac{\gamma_j\gamma_k}{2}\frac{1-3\cos^2\theta_{j,k}}{r_{j,k}^3} \quad \text{(S5)}$$

Here, $\mu_0 = 4\pi\times10^{-7}$ NA^{-2} is the magnetic constant and $r_{j,k}$ denotes the length of the vector, which forms an angle θ with the z axis and connects the two spins j and k. The typical magnitude of the interaction constants for the nearby nuclei in GaAs is $\max(|b_{j,k}|)/h \approx 100$ Hz. The Hamiltonian of Supplementary Eq. (S5) has been truncated to eliminate all spin non-conserving terms – this is justified for static magnetic field exceeding $\gtrsim 1$ mT. While the eigenstates of an isolated nucleus have well-defined spin projections m, the presence of the dipole-dipole interactions means that the true eigenstates of the nuclear spin ensemble in general cannot be written as product states of the single-nucleus states. The only two states where the nuclei are not entangled are the fully-polarized states, where all individual spins occupy the states with the same $m = -I$ or with the same $m = +I$. On the other hand, at high magnetic field the total z-projection operator $\sum_j \hat{I}_{{\rm z},j}$ approximately commutes with the nuclear spin Hamiltonian. Therefore, the nuclear ensemble eigenstates can be described by the well-defined total spin projections M. An example of an eigenenergy spectrum, calculated for $N = 6$ nuclei of ^{75}As, is shown in Fig. 4d of the main text. In this calculation we use $\nu_{\rm L} = 1.8$ kHz, $\nu_{\rm Q} = 0$ and the nuclei are taken from a single cubic cell of the group-III face-centered-cubic sublattice of GaAs. The bands observed in the spectrum correspond to the different values of M, ranging between $-NI$ and $+NI$. The broadening of each band is due to the dipole-dipole interactions, which lifts the degeneracy of the different states with the same M.

The interaction of the conduction band electron spin $\mathbf{s}$ with the ensemble of the QD nuclear spins is dominated by the contact (Fermi) hyperfine interaction, with the following Hamiltonian:

$$\mathcal{H}_{\rm hf}^{\rm e} = \sum_{j=1}^{N} a_j(\hat{s}_{\rm x}\hat{I}_{{\rm x},j} + \hat{s}_{\rm y}\hat{I}_{{\rm y},j} + \hat{s}_{\rm z}\hat{I}_{{\rm z},j}), \quad \text{(S6)}$$

where the hyperfine constant of an individual nucleus j is $a_j = A^{(j)}|\psi(\mathbf{r}_j)|^2 v$. Unlike a_j, the $A^{(j)}$ hyperfine constant is a parameter describing only the material and the isotope type to which nucleus j belongs, $|\psi(\mathbf{r}_j)|^2$ is the density of the electron envelope wavefunction at the nuclear site $\mathbf{r}_j$ of the crystal lattice, and v is the crystal volume per one cation or one anion. The definitions of the hyperfine constants differ between different sources. With the definition adopted here, a fully polarized isotope with spin I, hyperfine constant A and a 100% abundance (e.g. ^{75}As), would shift

the energies of the electron spin states $s_{\mathrm{z}} = \pm 1/2$ by $\pm AI/2$, irrespective of the shape of $|\psi(\mathbf{r}_j)|^2$. With such definition, the typical values in GaAs are $A \approx 50$ μeV [13].

For valence band holes the contact (Fermi) contribution vanishes, leaving the weaker dipole-dipole terms to dominate the hyperfine interaction. Compared to the valence band electrons, the coupling has a more complicated non-Ising form [9]. The effect of the net nuclear polarization on the heavy-hole spin splitting can be captured by a simplified expression:

$$\mathcal{H}_{\mathrm{hf}}^{\mathrm{h}} \approx \sum_{j=1}^{N} \frac{1}{3} C^{(j)} |\psi(\mathbf{r}_j)|^2 v \hat{j}_{\mathrm{z}} \hat{I}_{\mathrm{z},j}, \tag{S7}$$

where $\hat{j}_{\mathrm{z}}$ is the z component of the hole spin momentum operator. The valence band hyperfine material constants $C^{(j)}$ are sensitive to heavy-light hole mixing and both their signs and magnitudes depend on the material [9].

Owing to the flip-flop term $\propto (\hat{s}_{\mathrm{x}} \hat{I}_{\mathrm{x},j} + \hat{s}_{\mathrm{y}} \hat{I}_{\mathrm{y},j})$ of the hyperfine Hamiltonian (Supplementary Eq. S6) the eigenstates of the electron-nuclear central spin system are in general entangled, i.e. they cannot be written as a direct product of the electron spin single-particle state and the nuclear spin ensemble state. Consequently, when such a product state is generated through optical injection of a spin-polarized electron into the quantum dot, the wavefunction of the central spin system starts evolving. We estimate the rate of coherent evolution using the Rabi frequency $\propto \sqrt{\sum_j a_j^2}/h \approx A/(h\sqrt{N})$ derived previously in Ref. [14] for the limit of vanishing electron spin splitting. For a fully polarized nuclear spin ensemble coupled to an electron spin polarized in the opposite direction, this Rabi frequency describes the exact solution of periodic spin exchange between the electron and the collective nuclear spin state. Therefore, in order for dynamic nuclear spin polarization to be efficient, the polarized electron spins need to be removed and injected much faster than the hyperfine-induced Rabi rotations (otherwise the electron spin will periodically polarize and depolarize the nuclei, without any net spin transfer). For a typical GaAs QD with $N \approx 10^5$ nuclear spins we have $h\sqrt{N}/A \approx 25$ ns. When electron spin splitting is not zero, there is an increase in the frequency of coherent oscillations that follow initialization into a product electron-nuclear state. In the limit of large electron spin splitting this is approximately the electron spin resonance frequency. For experimental conditions used in our work the maximum sum of the net hyperfine shift and the electron Zeeman splitting at $B_{\mathrm{z}} = 10$ T does not exceed $\lesssim 180$ μeV, which corresponds to electron Larmor period of $\gtrsim 20$ ps. From these basic derivations, we arrive to a rough estimate that electron spin recycling must occur on a sub-picosecond timescale in order to achieve near-unity nuclear spin polarization.

Supplementary Note 3. EXPERIMENTAL METHODS AND TECHNIQUES

All measurements are performed in a liquid helium bath cryostat. The sample is placed in an insert tube filled with a low-pressure heat-exchange helium gas. The base temperature is ≈ 4.25 K. We use confocal microscopy configuration where QD photoluminescence (PL) is excited by a laser beam focused by a cryo-compatible apochromatic objective with a focal length of 2.89 mm and a numerical aperture of 0.81. The excitation spot diameter is ≈ 1 μm. Both the optical excitation and a static magnetic field $B_{\rm z}$ up to 10 T are applied along the sample growth axis z (Faraday geometry). Quantum dot photoluminescence is collected and collimated by the same cryo-compatible objective. The PL signal is dispersed in a two-stage grating spectrometer, followed by a pair of achromatic doublets, which transfers the spectral image onto a charge-coupled device (CCD) detector with a magnification of 3.75. The orientation of the semiconductor sample is verified by reflecting a collimated laser off the sample surface – the small unintentional tilt of the sample is found to be $\approx 0.7^{\circ}$. The laser used for optical pumping of the nuclear spins is a ring-cavity tunable titanium sapphire (Ti:Sa) laser, operating in a single-mode continuous-wave regime. This laser is coupled with a wavelength meter (30 MHz accuracy) for precise tuning and stabilization of the optical pumping wavelength. The sample gate bias is connected by a combination of a twisted pair (inside the cryostat) and a 50 Ω coaxial cable (outside the cryostat) to an arbitrary function generator through a low-pass LC filter with a 1.9 MHz cut-off frequency. Selective manipulation of the nuclear spins is achieved with a resonant radiofrequency oscillating magnetic field, generated by a small copper wire coil. This coil is placed to have its axis within the top surface of the semiconductor sample and perpendicular to the static magnetic field. A 50 Ω cryogenic coaxial cable is used to connect the coil to a radiofrequency amplifier with a maximum rated power of 100 W.

A. Pump probe experiment timing

Supplementary Fig. 3 shows the timing diagrams for the different types of experiments. Supplementary Fig. 3a shows the experimental cycle used in NMR spectroscopy, adiabatic sweep calibration and in nuclear spin polarization measurement (nuclear spin thermometry). The cycle consists of a radiofrequency burst between the pump and the probe optical pulses. During the cycle, the sample gate bias $V_{\rm Gate}$ is switched between the required levels by an arbitrary function generator. Both the pump and the probe optical pulses are implemented with mechanical shutters. A mechanical shutter on the spectrometer is synchronized with the probe laser shutter to prevent

the pump laser reaching the detector. Multiple pump-probe cycles, typically between 5 and 15, are accumulated by the CCD detector in order to improve the signal to noise ratio. For inverse NMR spectroscopy and adiabatic sweep calibration the pump duration is reduced to $T_{\mathrm{Pump}} = 5$ s to speed up the measurements. For saturation NMR spectroscopy and spin thermometry we use T_{Pump} between 25 s and 30 s in order to approach the steady-state of the nuclear spin polarization. The maximum T_{Pump} is limited by the need to collect photoluminescence from a sufficient number of probe pulses and the thermal noise of the CCD detector, which affects long exposures. While radiofrequency pulses can be applied at any bias, in this work we use $V_{\mathrm{Pump}} = -1.3$ V in order to keep the quantum dot free of charges during the radiofrequency manipulation of the nuclei.

Supplementary Fig. 3b shows a cycle used in the measurements of the nuclear spin buildup dynamics. Each cycle starts from a radiofrequency pulse that saturates the resonances of ^{75}As, ^{69}Ga, ^{71}Ga and ^{27}Al in order to depolarize these nuclei in the entire sample. Next, the pump pulse of a variable duration T_{Pump} is applied, and the resulting hyperfine shift is measured with a probe pulse.

Supplementary Fig. 3c shows a cycle used in the nuclear spin relaxation measurements. The cycles start with radiofrequency depolarization that is sufficiently long to eliminate any effect of the nuclear polarization left over from the previous measurement cycle. This is followed by optical pumping for $T_{\mathrm{Pump}} = 60$ s. After the pump, the sample is kept in the dark for a time T_{Dark} under gate bias V_{Dark}. After that, a probe pulse is applied to measure the fraction of the nuclear spin polarization that decayed during T_{Dark}. In this type of experiments the number of the pump-probe cycles used to collect the probe photoluminescence signal varies between 1 and 10 – single-shot probing is required when T_{Dark} exceeds a few hundred seconds. Furthermore, we perform measurements using the cycle of Supplementary Fig. 3c, but with a second radiofrequency (Rf) pulse added after the pump and before the dark interval. The duration of this second Rf pulse $T_{\mathrm{Rf,2}}$ is typically varied between 0 and 5 s to control the degree of the initial nuclear spin polarization. In principle, there are multiple ways to control the degree of the initial nuclear spin polarization in the quantum dot, such as the duration T_{Pump} of the pump or its power and wavelength. However, any such changes in the optical pumping also affect the rate of nuclear spin diffusion into the barriers around the quantum dot [15]. The degree of nuclear polarization in the barriers then affects the rate of nuclear spin relaxation in the subsequent dark interval. The advantage of the second radiofrequency pulse is that it depolarizes the nuclei at the same rate in the entire sample. Therefore, the spatial profile of the nuclear spin polarization P_{N} after the second pulse is simply a scaled profile of the P_{N} profile produced by the optical pulse. Spin diffusion

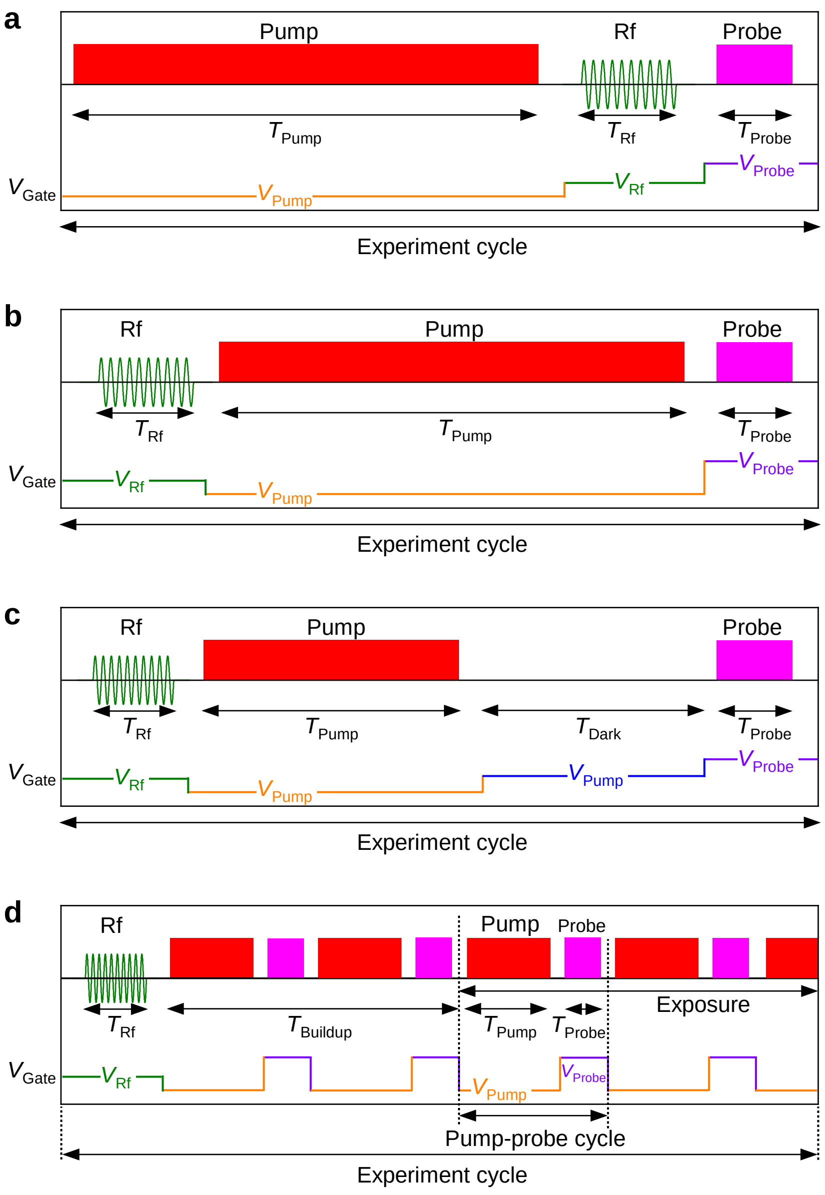

Supplementary Figure 3. **Timing diagrams of different experiments.** **a,** NMR spectroscopy and measurement of nuclear spin polarization. **b,** Nuclear spin buildup dynamics. **c,** Nuclear spin relaxation in the dark. **d,** Optical pump power and wavelength dependence of the steady state nuclear spin polarization.

is described by a linear differential equation, so that proportional reduction of P_{N} in the entire sample should not affect the timescales of the subsequent nuclear spin diffusion and relaxation in the dark. Consequently, any dependence of the relaxation time on the degree of the initial nuclear spin polarization (left after the second radiofrequency pulse) is ascribed purely to the reduction (narrowing) of the energy that the dipole-dipole reservoir can supply or absorb during the nuclear flip-flop events of the spin diffusion process. We note that the second radiofrequency pulse has

minimal effect on the measurement of the subsequent relaxation dynamics, since its duration is no more than 10% of the shortest measured nuclear spin relaxation time $T_{1,\mathrm{N}}$ (except for one measurement with $T_{\mathrm{Rf},2} = 18$ s).

Supplementary Fig. 3d shows the timing of the experiment used to study the dependence of the steady-state nuclear spin polarization on the optical pumping parameters such as gate bias V_{Pump}, pump power and wavelength. The experiment cycle starts with a single radiofrequency erase that eliminates any leftover nuclear polarization. Then the pump and probe pulses start, but the acquisition (CCD detector exposure) of the probe photoluminescence begins only after a delay $T_{\mathrm{Buildup}} = 50$ s. This delay allows nuclear spin polarization to build up closer towards its steady state so that relatively short pump pulses $T_{\mathrm{Pump}} = 5$ s can be used for faster acquisition of the photoluminescence signal. The probe pulses are kept short, in order to produce minimal nuclear spin depolarization during each pump-probe cycle (see details in Supplementary Note 3 C). The probe pulses are also much shorter than the pump ($T_{\mathrm{Probe}}/T_{\mathrm{Pump}} < 0.003$), to ensure minimal effect on the steady-state nuclear spin polarization.

B. Optical pumping of quantum dot nuclear spins

The steady-state nuclear spin polarization depends on the wavelength of the pump laser. Maximum hyperfine shifts $|E_{\mathrm{hf}}|$ are found to occur when the laser is resonant with a certain optical transition of the quantum dot. Calibration of the optimal pumping parameters starts with a measurement of a broad-range wavelength dependence – an example is shown in Supplementary Fig. 4. Once the individual spectral features, such as s-, p-, d- and f-shell exciton peaks, are identified a more detailed optimization is performed. We focus on the s-shell pumping peak and measure more detailed dependencies on the wavelength (or equivalently the pump photon energy) at different values of pump power P_{Pump} and sample gate bias V_{Pump}. The inset in Supplementary Fig. 4 shows an example of such a detailed dependence at the optimum $P_{\mathrm{Pump}} = 2.7$ mW, $V_{\mathrm{Pump}} = -2.7$ V. It can be seen that the pump laser needs to be tuned to within a narrow margin of ≈ 0.2 meV in order to achieve the highest possible $|E_{\mathrm{hf}}|$.

In the experimental setup the collimated pump laser beam first passes through a linear polarizer and then a $\lambda/2$ waveplate installed in a motorized rotation mount. This way it is possible to create arbitrary orientation of the linearly polarized beam, which is then directed to a cube beamsplitter, followed by a $\lambda/4$ waveplate installed in another motorized rotation mount. By placing the $\lambda/4$ waveplate last, it is possible to compensate for any polarization imperfections of the nominally non-

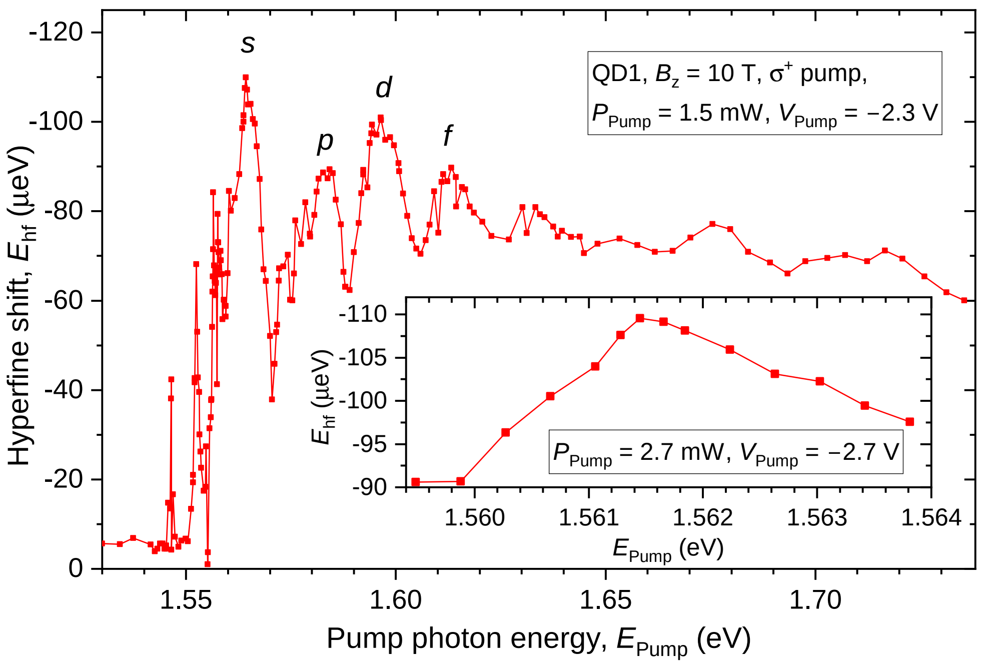

Supplementary Figure 4. **Calibration of the pump laser wavelength.** Hyperfine shift measured as a function of the pump laser wavelength expressed in terms of the photon energies $E_{\rm Pump}$. Results are shown for QD1 at $B_{\rm z} = 10$ T under σ^+ pumping. The pump power is $P_{\rm Pump} = 1.5$ mW and the gate bias is $V_{\rm Pump} = -2.3$ V. Inset shows a zoomed in dependence for the s-shell peak measured at slightly different optimized parameters $P_{\rm Pump} = 2.7$ mW, $V_{\rm Pump} = -2.7$ V.

polarizing beamsplitter and obtain a beam with high degree of circular polarization. This beam is then directed through a quartz window of the cryostat insert and the cryogenic objective, which focuses it on the surface of the QD semiconductor sample. In order to account for any polarization imperfections in the optical path we perform calibration measurements where both the $\lambda/2$ and $\lambda/4$ waveplate orientations are scanned and the resulting hyperfine shifts $E_{\rm hf}$ are measured. The results shown in Supplementary Fig. 5, indicate that the waveplate must be set within $\pm 2^\circ$ in order to attain the highest nuclear spin polarization degree. The optimal orientations of the $\lambda/2$ and $\lambda/4$ waveplates are different for the minimum negative (triangles) and the maximum positive (squares) $E_{\rm hf}$.

Once the orientations of the $\lambda/2$ and $\lambda/4$ waveplates are optimised, we examine the polarization state of the pump beam directed to the cryostat. To this end, we place a linear polarizer (analyzer) after the $\lambda/4$ waveplate, followed by a power meter. The linear polarizer is rotated to find the minimum ($I_{\rm min}$) and the maximum ($I_{\rm max}$) intensities of the transmitted beam. For a perfect circularly polarized beam $I_{\rm min} = I_{\rm max}$, whereas for a linearly polarized beam $I_{\rm min} = 0$. We

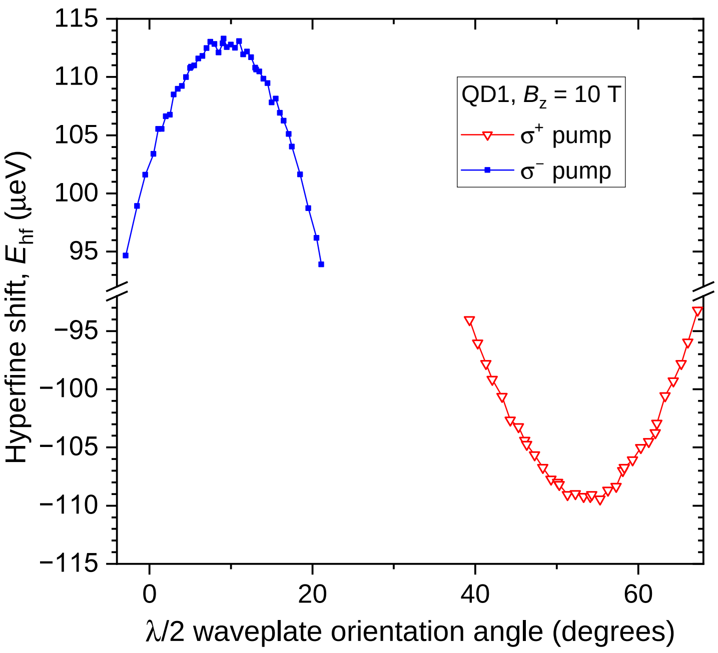

Supplementary Figure 5. **Calibration of the optical polarization of the pump.** Hyperfine shift measured as a function of the orientation angle of a $\lambda/2$ waveplate installed in the pump laser beam. Other parameters, such as $\lambda/4$ waveplate orientation, pump power, wavelength and bias are optimized separately for both σ^+ (triangles) and σ^- (squares) pumping of the QD nuclear spin polarization. Results are shown for QD1 at $B_z = 10$ T.

characterise the optimized beams using the degree of linear polarization $\rho_{\rm lin} = (I_{\rm max} - I_{\rm min})/I_{\rm max}$ and the analyzer orientation angle $\alpha_{\rm max}$ where the maximum intensity is achieved. These results are summarized in the polar plot of Supplementary Fig. 6.

The optimal degree $\rho_{\rm lin}$ and orientation $\alpha_{\rm max}$ of the linearly polarized components vary between individual quantum dots. Moreover, optimal polarization parameters are different for σ^+ and σ^- pumping and even depend on magnetic field for the same QD1. Such variability, as well as the large values of $\rho_{\rm lin} \lesssim 0.4$ suggest that polarization imperfections in the optical elements (e.g. mechanical stress in the cryo-objective) are not the major contribution. The large deviation of the optimal optical pumping from pure circular polarization is therefore attributed to the properties of the individual quantum dots. These may include anisotropy of the QD shape and inhomogeneous microstrains that make semiconductor material around the QD act as an optical waveplate and give rise to heavy-light hole mixing [16] that causes optical selection rules to depart from those of the bulk GaAs. Indeed, previous studies have shown that a sufficiently large uniaxial strain $\gtrsim 0.5$ % can flip the valence band hole quantization axis into the sample growth plane [17]. We note that

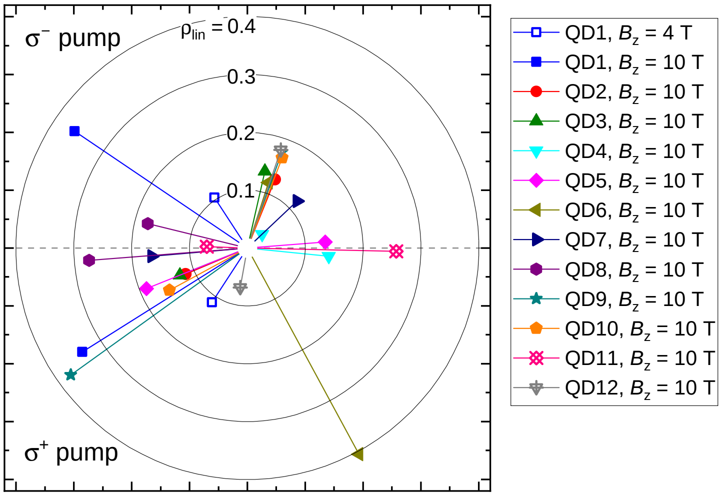

Supplementary Figure 6. **Optical polarization properties of the optimal nuclear spin pumping beams.** Vectors show the orientation of the linear polarization and the magnitude of the linear polarization degree ρ_{lin} of the pump beam that produces maximum nuclear spin polarization in individual dots QD1 - QD12. The bottom part shows the results for minimum negative hyperfine shift (σ^+ character of the circularly polarized component of the pump) while the top part shows results for maximum positive hyperfine shift (σ^- character). The horizontal and vertical axes of the plot correspond approximately to the edges of the semiconductor sample, cleaved along the [110] and [1$\bar{1}$0] crystallographic directions.

the indirect effect of strain on nuclear spin cooling through mixing of the valence band states should be distinguished from the direct effect through modification of the nuclear spin eigenstates discussed in Supplementary Note 2.

Another measure of the QD anisotropy is the fine structure splitting (FSS) of a neutral exciton at zero magnetic field. While we have not conducted systematic correlation studies, selective measurement on QD1, where very large nuclear spin polarization has been achieved, reveals a FSS of ≈ 28 μeV. This is considerably larger than the few-μeV FSS observed in symmetric GaAs QDs [18]. This comparison suggests that QD anisotropy does not preclude large nuclear spin polarization, as long as optical selection rules permit coupling to spin-polarized conduction band electronic states. Depending on the type of anisotropy, the heavy hole state $j_z = +3/2$ is mixed with the $j_z = -1/2$ or the $j_z = +1/2$ light-hole state. In case of $j_z = -1/2$ admixing to $j_z = +3/2$ the resulting exciton state becomes elliptically polarized, whereas the admixture of $j_z = +1/2$ to $j_z = +3/2$ is associated with optical transition dipole element polarized along the sample growth axis z [19, 20]. From that perspective, optimization of the $\lambda/2$ and $\lambda/4$ waveplate angles can be understood as matching of the optical pump polarization to the elliptical polarization of

the QD optical transition, allowing generation of spin-polarized electrons. In case of z-polarized optical transition, such polarization matching is not possible, which may explain why nuclear spin polarization degree is much lower than unity in some of the QDs (Fig. 3f of the main text) even after optimizing the elliptical polarization of the pump laser.

It is also possible that spin-polarized holes contribute to nuclear spin pumping, although the hyperfine flip-flops of the heavy holes are expected to be much smaller than for electrons [9]. Further investigations (both experimental and theoretical) would be needed to elucidate which types of QD anisotropies are compatible with efficient nuclear spin polarization. For brevity, throughout this work we use the term "σ^+ pumping" ("σ^- pumping") to describe the optimized elliptically-polarized optical pumping beam with a σ^+ (σ^-) character of the circularly polarized component.

C. Optical probing of quantum dot nuclear spins

For optical probing of the nuclear spin polarization we use a diode laser emitting at 690 nm. Sample forward bias, typically +0.7 V, and the probe power are chosen to maximize (nearly saturate) PL intensity of the ground state X^- trion. The difference between the spectral splitting $\Delta E_{\rm PL}$ of the X^- trion doublet and the same splitting $\Delta E_{\rm PL,0}$ measured for depolarized nuclei reveals the hyperfine shifts $E_{\rm hf} = -(\Delta E_{\rm PL} - \Delta E_{\rm PL,0})$. Illumination with a probe laser inevitably acts back on the nuclear spin polarization. In order to quantify such back-action we perform calibration measurements with examples shown in Supplementary Fig. 7. In these experiments the QD is first pumped with a σ^+ or σ^- polarized laser with power and bias set to maximize the steady state nuclear polarization. Then the pump is switched off and the probe laser pulse is applied. The hyperfine shift $E_{\rm hf}$ is measured from PL spectroscopy at the end of this probe. It can be seen that the probe induces decay of the nuclear spin polarization. For QD1 the unwanted probe-induced depolarization is faster at $B_{\rm z} = 4$ T (solid symbols) compared to $B_{\rm z} = 10$ T (open symbols), even though the same probe power of $P_{\rm Probe} = 30$ nW is used at both fields. For selective-NMR measurements of the nuclear spin polarization (spin thermometry) in QD1 we use $T_{\rm Probe} = 15$ ms at $B_{\rm z} = 10$ T, so that the resulting depolarization is negligible ($< 1\%$). PL intensity of the same QD1 is weaker at $B_{\rm z} = 4$ T so we use a longer $T_{\rm Probe} = 24$ ms in order to obtain a sufficiently strong probe PL signal. However, this leads to a larger depolarization of $\approx 4\%$ under σ^- pumping. Depolarization itself is not an issue, since it would simply rescale all the measured $E_{\rm hf}$, which would not affect the differential NMR spin thermometry. In practice, the probe-induced depolarization

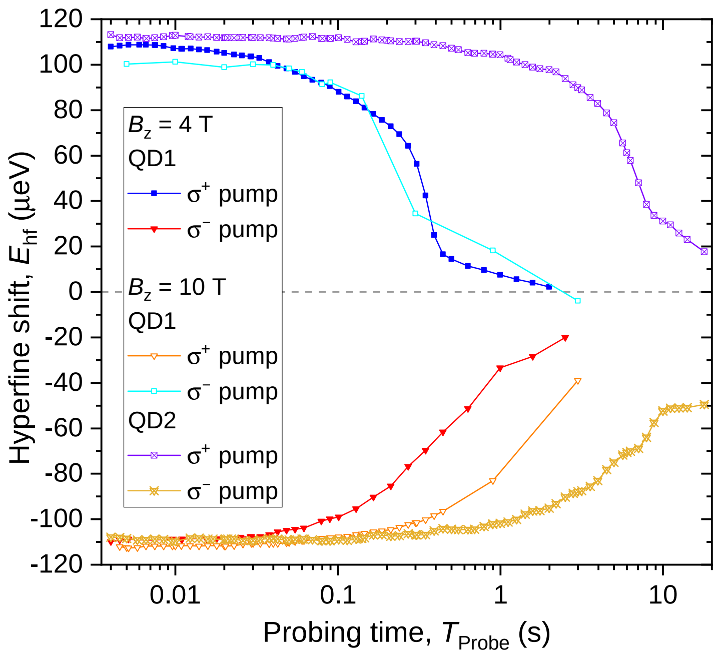

Supplementary Figure 7. **Calibration of the optical probing of the QD nuclear spin polarization.** Hyperfine shift measured as a function of the probing time T_{Probe} following a σ^+ (triangles) or σ^- (squares) pumping of the nuclear spin polarization in a QD. For QD1 results are shown for $B_z = 4$ T (solid symbols) and $B_z = 10$ T (open symbols), measured with a probe power of $P_{\text{Probe}} = 30$ nW. For QD2 results are shown for $B_z = 10$ T (crossed symbols), measured with a probe power of $P_{\text{Probe}} = 7$ nW.

also depends on the instantaneous E_{hf} – such nonlinearity is what causes the distortion, resulting in larger uncertainties of the nuclear spin polarization measured at $B_z = 4$ T. For QD2 we use a lower probe power $P_{\text{Probe}} = 7$ nW (crossed symbols in Supplementary Fig. 7), which leads to an even slower probe-induced depolarization than for QD1. This allows to have a longer probe pulse ($T_{\text{Probe}} = 40$ ms) for QD2, while keeping parasitic depolarization small ($< 1\%$).

D. Radiofrequency control of nuclear spins

The radiofrequency oscillating magnetic field $B_x \perp z$ is produced by a coil placed at a distance of ≈ 0.5 mm from the QD sample. The coil is made of 10 turns of a 0.1 mm diameter enameled copper wire wound on a ≈ 0.4 mm diameter spool in 5 layers, with 2 turns in each layer. Two main types of radiofrequency signals are used in this work. The first type is a frequency-swept monochromatic excitation which is used for adiabatic inversion of the nuclear spin population. The amplitude of the radiofrequency field is constant and the frequency is swept linearly in time. Radiofrequency sweeps are discussed further in Supplementary Note 5 D.

The second type is the broadband radiofrequency excitation which is required to saturate inhomogeneously broadened quadrupolar resonances. The typical width of the resonances that needs to be saturated is tens to hundreds of kHz (further details are given in Supplementary Note 5 B), which is significantly larger than the typical homogeneous NMR linewidth ($<$ 1 kHz). Therefore monochromatic radiofrequency excitation cannot provide a sufficiently uniform saturation of the entire inhomogeneously broadened resonance. This necessitates the use of a broadband radiofrequency excitation. Ideally, one wants a signal with a rectangular spectral profile, which has a constant spectral density in the required frequency interval, and a zero intensity outside that interval. In practice, when implementing the radiofrequency waveforms on a digital generator, it is convenient to approximate the required rectangular spectral band with a frequency comb. In spectral domain, the comb consists of periodically spaced monochromatic modes of constant amplitude, covering the desired interval of frequencies. The mode spacing of 120 Hz is chosen to be smaller than the homogeneous NMR linewidth. Under these conditions, by using a sufficiently small amplitude of each mode we achieve exponential depolarization (i.e. without nuclear spin Rabi oscillations) of the nuclear spin ensemble [21] with a typical time constant of $\tau \approx 30$ ms. The saturation of a chosen NMR resonance is achieved by applying a frequency comb excitation for a period of $\approx 5\tau$. When subject to such excitation, the nuclear spins undergo slow Rabi rotation, transitioning between the spin states parallel and antiparallel to the external magnetic field [22]. Due to the nuclear-nuclear dipole-dipole interactions each nuclear spin is subject to a local field. The randomness of these local fields results in dephasing between Rabi precessions of the individual nuclei. Consequently, the nuclear spin ensemble becomes depolarized (i.e. each nucleus is randomly polarized) after a long saturation pulse.

For the saturation NMR spectra, shown in Fig. 3b of the main text, we use a frequency comb with a total width of 6 kHz. The central frequency of the comb is scanned to obtain the spectra – this frequency is the horizontal axis of the spectral plots. For the high-resolution NMR spectra, shown in Fig. 3a of the main text, we employ the "inverse NMR" technique [23] which enhances the NMR signal and allows the spectra to be measured even on those nuclear spin transitions that are depopulated at high polarization degrees. In this approach the radiofrequency excitation spectrum is a broadband frequency comb with a narrow gap. The central frequency of the gap is scanned and is used for the horizontal axis of the "inverse NMR" spectra. The width of the gap controls the balance between the NMR signal amplitude and the spectral resolution. For the spectra of Fig. 3a of the main text we use a 4 kHz gap to measure the satellite transitions and a 2 kHz gap to measure the narrow central transition NMR peak. The spectra of Fig. 3a are measured with

σ^+ optical pumping. When measured with σ^- pumping, the NMR spectral peaks are shifted to a higher frequency, typically by ≈ 1 kHz. This shift is attributed to dipolar interaction of the measured isotope with the polarized nuclei of the other isotopes.

During the NMR measurements, the superconducting magnet is set into persistent mode, resulting in a predictable nearly-linear decay of the NMR frequencies with time. The typical relative decay rate is $\approx 5 \times 10^{-10}$ s^{-1}. The NMR measurements are programmed to track this decay of the persistent magnetic field. The typical errors in NMR frequencies resulting from uncertainty in the decay rate are within 1 kHz. Some apparent shifts between the same NMR peaks plotted in Fig. 3a and in Fig. 3b of the main text include these contributions of the dipolar interactions and the decaying magnetic fields. In addition, the difference in the spectral resolutions causes some apparent shifts. In particular, in the higher-resolution spectra (4 kHz, Fig. 3a of the main text) the satellite transitions appear as asymmetric peaks. By contrast, in the lower-resolution spectra (6 kHz, Fig. 3b of the main text) the same peaks are smoothed and appear shifted.

Supplementary Note 4. ADDITIONAL EXPERIMENTAL DATA AND DISCUSSION

A. Extended data from nuclear spin pumping measurements

Our approach to maximizing the nuclear spin polarization is through line-search optimization of the optical pumping parameters, such as pump photon energy E_{Pump}, pump power P_{Pump}, optical polarization and the sample bias V_{Pump}. In order to understand the physics of the nuclear spin pumping process we also measure a systematic parametric dependence. Given the typical timescales of the nuclear spin process ($1-100$ s) it is not possible to explore the entire parameter space within a reasonable experimental time. Therefore, we measure various one- and two- dimensional sections in the multidimensional parameter space. An example is shown in Supplementary Fig. 4, where a one-dimensional dependence on E_{Pump} is shown. The spectral dependence of the nuclear spin polarization is interpreted by comparing it with photoluminescence spectroscopy.

Supplementary Fig. 8a is a bias-dependent photoluminescence spectroscopy map measured under non-resonant optical excitation (HeNe laser emitting at 632.8 nm). At low excitation power $P_{\text{Exc}} = 0.5$ nW we observe multiple spectral features, labelled accordingly. The emission of the bulk GaAs free exciton is observed at ≈ 1.518 eV, accompanied by a low energy band at ≈ 1.50 eV arising from doping and impurities. The broad emission between $\approx 1.57 - 1.67$ eV is attributed to both the Si-doped and C-doped AlGaAs layers. The peak at ≈ 1.675 eV is also ascribed to

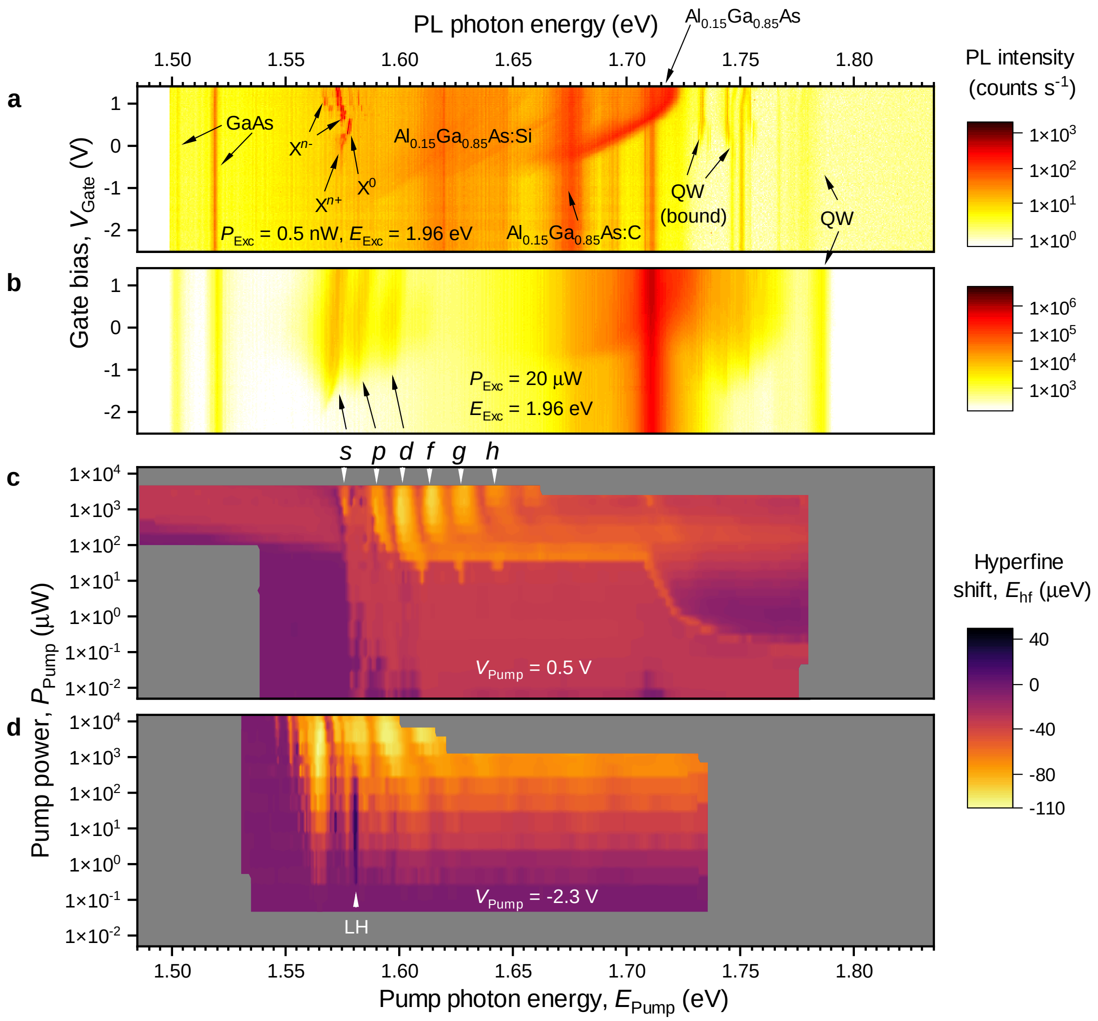

Supplementary Figure 8. **Power dependence of nuclear spin pumping. a,** Bias dependent photoluminescence spectra of QD1 at $B_z = 10$ T measured at a low excitation power $P_{\rm Exc} = 0.5$ nW. Optical excitation is continuous wave at a wavelength of 632.8 nm. Each spectrum is an average of three 20 s long exposures. **b,** Same photoluminescence spectra but at a high power of $P_{\rm Exc} = 20$ μW. Each spectrum is an average of three 50 ms long exposures. **c,** Steady-state hyperfine shift measured as a function of the pump power $P_{\rm Pump}$ and the pump photon energy $E_{\rm Pump}$. Measurement is conducted on QD1 at $B_z = 10$ T using σ^+ pump polarization. The gate bias is kept at $V_{\rm Pump} = +0.5$ V during pumping. The regions where no data was measured are shown in gray. **d,** Same as (c) but for $V_{\rm Pump} = -2.3$ V. "LH" labels the feature ascribed to the resonant absorption of a light-hole exciton.

AlGaAs:C. The undoped $Al_{0.15}Ga_{0.85}As$ layers emit at ≈ 1.71 eV under positive bias $V_{\rm Gate} \approx +1$ V. When the bias is reduced to $V_{\rm Gate} \approx 0$ V the emission of the $Al_{0.15}Ga_{0.85}As$ electron tunnel barrier Stark-shifts down to ≈ 1.675 eV. By contrast, the luminescence of the $Al_{0.15}Ga_{0.85}As$ layer bellow the n-type barrier is not affected by the applied electric field and remains at ≈ 1.71 eV.

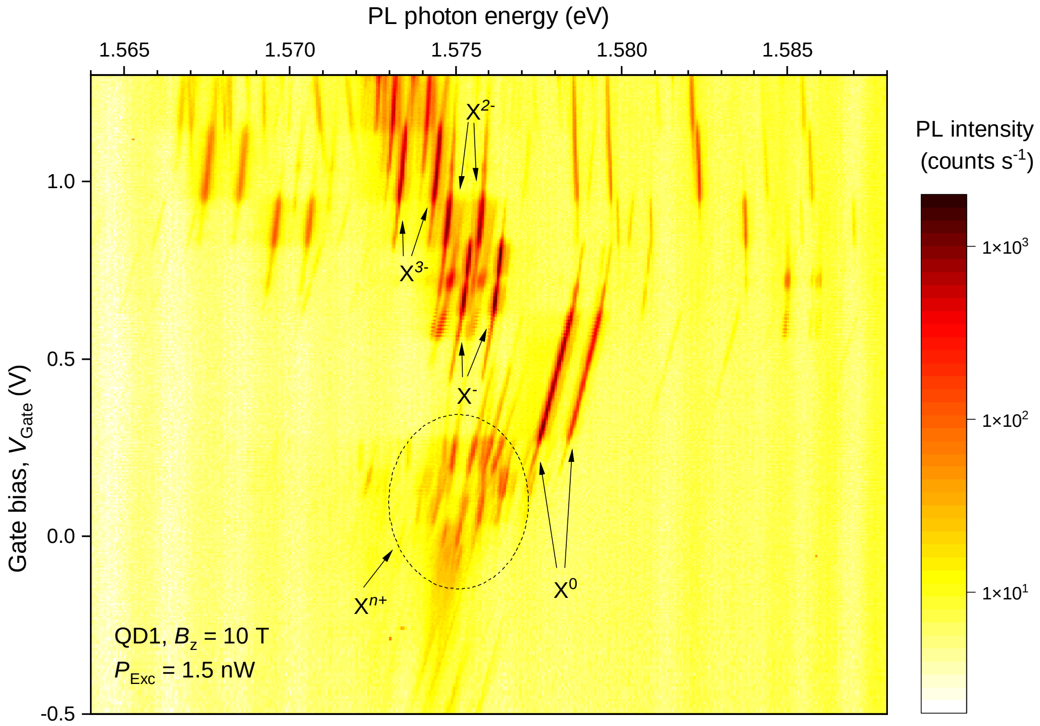

Supplementary Figure 9. **Photoluminescence spectroscopy of QD excitons. a,** Bias dependent photoluminescence spectra of QD1 at $B_\mathrm{z} = 10$ T measured at a low excitation power $P_\mathrm{Exc} = 1.5$ nW and an excitation wavelength of 632.8 nm.

The emission at ≈ 1.785 eV is ascribed to the quantum well (QW) formed when the nanoholes are infilled with GaAs. The narrower lines observed at low excitation power around $\approx 1.73 - 1.75$ eV are attributed to the QW excitons bound by the thickness fluctuations of the QW. Single-QD emission is observed between $\approx 1.56 - 1.59$ eV as a series of Zeeman doublets that switch over as the gate bias V_Gate is changed. The higher-resolution spectra of the QD excitons are shown in Supplementary Fig. 9. At $V_\mathrm{Gate} \approx +0.5$ V photoluminescence is dominated by the neutral exciton X^0, identified from its fine structure splitting at $B_\mathrm{z} = 0$ T. At more negative biases the emission of positively charged excitons dominates, since electrons rapidly tunnel out of the dot, leaving excess photogenerated (non-equilibrium) holes. At more positive biases the emission of X^0 is superseded by the negatively charged trion X^-, which becomes dominant when QD confines a resident (equilibrium) electron. At even more positive biases the dot is charged with multiple resident electrons. The spectral features originating from doubly (X^{2-}) and triply (X^{3-}) charged excitons can be distinguished, while the photoluminescence peaks at even higher charge numbers tend to overlap.

When the power is increased ($P_\mathrm{Exc} = 20$ μW in Supplementary Fig. 8b) the emission peaks of

the s-shell QD excitons with different charging broaden into a single red-shifted band (≈ 1.572 eV). Emission of the higher-shell excitons (p, d and f) also becomes visible. By contrast, photoluminescence intensity of the AlGaAs layer (located below the doped region) increases with power without saturation.

Supplementary Fig. 8c shows the two-dimensional map of the hyperfine shift $E_{\rm hf}$ measured as a function of the pump power $P_{\rm Pump}$ and photon energy $E_{\rm Pump}$ at a fixed $V_{\rm Pump} = +0.5$ V, which roughly corresponds to a bias where the QD equilibrium state switches from 0 to 1 electron. Nuclear spin pumping evidently takes place at powers as low as $P_{\rm Pump} \approx 10$ nW (which is close to saturation power of the s-shell QD excitons) as long as the pump laser is tuned above the s-shell exciton transition, so that the QD can absorb the pump photons. However, the resulting nuclear spin polarization degree is low, characterized by $E_{\rm hf} \approx -30$ μeV. Spin pumping efficiency increases when the pump power is increased to hundreds of μW, which is well above the ground state exciton saturation. The lowest negative $E_{\rm hf} \approx -90$ μeV is achieved at $P_{\rm Pump} \approx 1$ mW. At this high power, a series of spectral peaks is observed. Their periodicity matches the periodicity observed in the high-power photoluminescence spectra (Supplementary Fig. 8b), which allows us to identify the peaks as originating from different excitonic shells (up to six visible). The mechanism of nuclear spin pumping can then be understood to arise from resonant absorption of the circularly polarized pump photons, which generate spin-polarized electrons and holes in the excited orbital states. It also follows from Supplementary Fig. 8c that at $V_{\rm Pump} = +0.5$ V the steady-state $|E_{\rm hf}|$ produced by pumping via the higher d and f shells is larger than via the p and ground s shells. Excitation via higher shells means that excitons can relax towards the ground state before recombination. Such energy relaxation provides a route for a simultaneous exchange of spin with the nuclei [24], since it helps to absorb or supply a small amount of energy required to compensate the mismatch of the electron and nuclear Zeeman energies. Without the coupling to external energy reservoirs the electron-nuclear spin flip-flop would be energetically forbidden.

At high powers $P_{\rm Pump} \gtrsim 100$ μW, nuclear spins can be polarized even via optical excitation well below the ground state QD exciton transition, indicating that it's a distinct spin pumping mechanism which we further discuss below.

Supplementary Fig. 8d shows the same dependence of $E_{\rm hf}$ on $P_{\rm Pump}$ and $E_{\rm Pump}$ but with the sample gate voltage changed to a large reverse bias regime $V_{\rm Pump} = -2.3$ V. At this bias, no QD photoluminescence is observed, even at high optical excitation power, meaning that the excitons become ionized before they can recombine to emit a photon. Nevertheless, nuclear spin pumping is observed and is more efficient than at $V_{\rm Pump} = +0.5$ V. Under large reverse bias, a higher

threshold power (of a few μW) is needed to induce measurable nuclear spin polarization, which can be explained by the need for the optical excitation to outpace the fast tunneling of the charges from the QD. We again observe spectral peaks that can be matched to the individual excitonic shells (at $V_{\rm Pump} = -2.3$ V the shells are red-shifted with respect to $V_{\rm Pump} = +0.5$ V because of the Stark shift). Large $|E_{\rm hf}|$ are observed for all four lowest exciton peaks, but from experiments on multiple individual QDs we consistently find that pumping through the ground state s-shell exciton under large reverse bias leads to the most efficient spin pumping (characterized by the highest $|E_{\rm hf}|$).

It is also worth noting that inverted $E_{\rm hf}$ is observed under certain pumping conditions. For example, in Supplementary Fig. 8d we observe $E_{\rm hf} > 0$ around $P_{\rm Pump} \approx 10$ μW and $E_{\rm Pump} \approx$ 1.580 eV, which is $\approx$ 16 meV above the energy of the s-shell nuclear spin pumping peak. This feature at $E_{\rm Pump} \approx 1.580$ eV, labelled "LH", is ascribed to a light-hole exciton. Optical excitation of a heavy-hole exciton transition with a σ^+ polarized light (with photons carrying a +1 momentum in units of $\hbar$) generates a hole with momentum projection $j_{\rm z} = +3/2$ and an electron with a $s_{\rm z} = -1/2$ spin projection. The $s_{\rm z} = -1/2$ electrons then lead to nuclear spin pumping with a negative $E_{\rm hf} < 0$, as indeed observed in Supplementary Fig. 8d for a wide range of the pump parameters. However, when resonant with a light-hole exciton transition, the same σ^+ photon generates a hole with $j_{\rm z} = +1/2$ and an electron with $s_{\rm z} = +1/2$ (see Supplementary Fig. 2a), which then leads to an inverted $E_{\rm hf} > 0$. The same argument applies to σ^- optical excitation, and manifests in experiments as $E_{\rm hf} < 0$, observed under resonant excitation of the light-hole exciton transition.

In order to investigate the nuclear spin pumping mechanisms further, we fix the pump power at $P_{\rm Exc} = 1.5$ mW and measure $E_{\rm hf}$ as a function of $E_{\rm Pump}$ and $V_{\rm Pump}$. The results are presented in Supplementary Fig. 10c, which shows an extension of the data from Fig. 2c of the main text. The spectral peaks, ascribed to individual excitonic shells, are seen to Stark-shift with the applied bias (Supplementary Fig. 10c). The largest hyperfine shift $|E_{\rm hf}|$ is again observed for the s-shell exciton at reverse bias, which varies between -2.7 and -2.1 V for different individual QDs. The higher-shell excitons (p, d, etc.) differ from the s shell in that their excitation can be followed by relaxation into a lower energy shell. The slightly lower $|E_{\rm hf}|$ can then be ascribed to such relaxation between shells, which may involve flipping of the electron without spin transfer to the nuclei. In other words, relaxation between shells may result in a reduced electron spin polarization, which in turn leads to reduction of the maximum achievable $|E_{\rm hf}|$. Resonant pumping into the s-shell can only generate up to two electrons and two holes in the QD. This leaves only four excitonic complexes that can take part in dynamic nuclear spin polarization: neutral exciton X^0, neutral biexciton

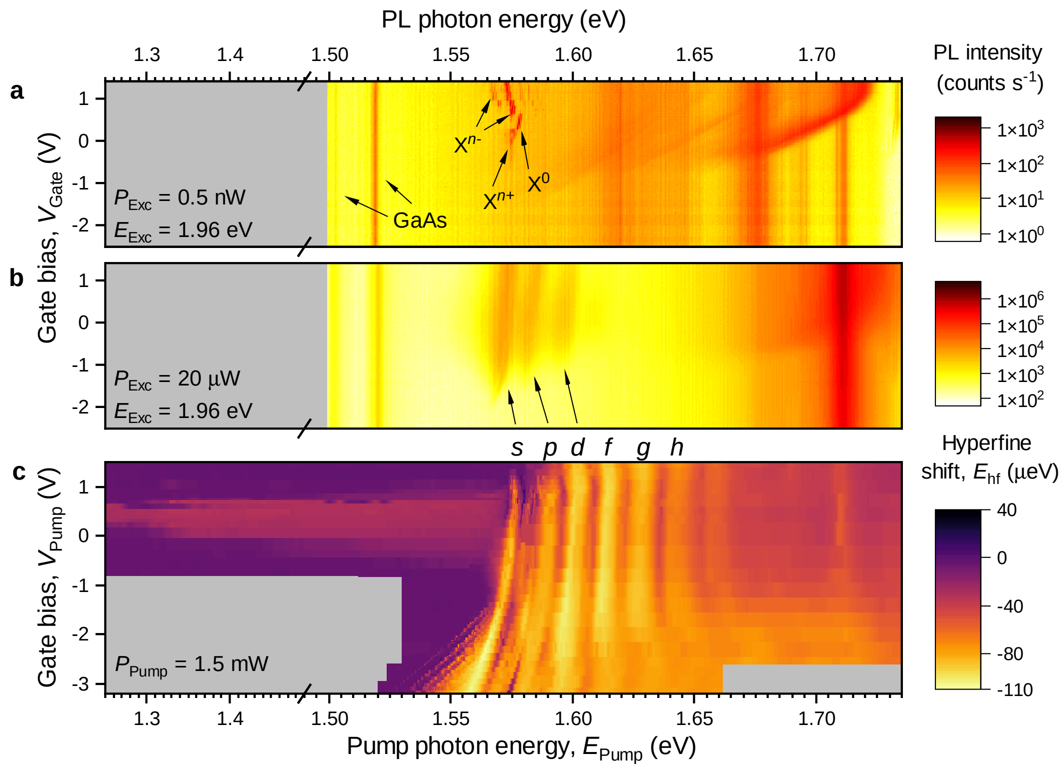

Supplementary Figure 10. **Bias dependence of nuclear spin pumping. a, b,** Same bias-dependent photoluminescence spectra as in Supplementary Figs. 8a,b reproduced for reference. The scale of the horizontal axis is changed at 1.49 eV for better visibility and resolution. **c,** Steady-state hyperfine shift measured as a function of the pump bias V_{Pump} and the pump photon energy E_{Pump}. Measurement is conducted on QD1 at $B_{\mathrm{z}} = 10$ T. Excitation power is $P_{\mathrm{Exc}} = 1.5$ mW with a σ^+ polarization. The regions where no data was measured are shown in gray.

XX^0, negatively charged trion X^- and positively charged trion X^+. We have performed the same measurements as in Supplementary Figs. 4 and 10c but with σ^- pump polarization (producing positive E_{hf}). We find that the spectral positions E_{Pump} of the optimal nuclear spin pumping peaks under σ^+ and σ^- pumping are split by ≈ 900 μeV at $B_{\mathrm{z}} = 10$ T, matching excitonic spectral splitting observed in photoluminescence (Supplementary Fig. 9). However, it is not possible to determine directly which excitonic feature is responsible for nuclear spin pumping with maximum $|E_{\mathrm{hf}}|$. The biexciton XX^0 is unlikely to play a role – it consists of an electron spin singlet (two electrons, one with spin projection $s_{\mathrm{z}} = -1/2$ and one with $s_{\mathrm{z}} = +1/2$) and a hole spin singlet (two holes, one with momentum projection $j_{\mathrm{z}} = -3/2$ and one with $j_{\mathrm{z}} = +3/2$) which do not couple to nuclear spins. The X^- trion is also unlikely to cause efficient nuclear spin pumping, because the two electrons are in a singlet state. Moreover, spin-selective optical excitation of

X^- requires prior injection of another spin-flipped electron, for which there is no sufficiently fast process that could compete with rapid tunneling. The X^+ trion is more likely to contribute to nuclear spin pumping, since it contains only one (spin-polarized) electron. However, to form a hole spin singlet, X^+ excitation would still need to be accompanied by hole spin flipping, which would create a bottleneck and slow down the cyclic nuclear spin pumping process. Therefore, we argue that resonant optical excitation of X^0 is the most likely route for efficient nuclear spin pumping, as it enables fast optical reexcitation upon tunneling of the previously-excited electron-hole pair out of the QD. In addition to the broad resonance that gives the most efficient nuclear spin pumping ($E_{\mathrm{Pump}} \approx 1.564$ eV at $V_{\mathrm{Pump}} = -2.3$ V in Supplementary Fig. 10c), there are narrower and less efficient Stark-shifting resonances observed at lower E_{Pump} (intersecting the same $V_{\mathrm{Pump}} = -2.3$ V around $E_{\mathrm{Pump}} \approx 1.552$ eV and $E_{\mathrm{Pump}} \approx 1.546$ eV). These narrow resonances may correspond to optical excitation of X^+ and X^-. This would be consistent with photoluminescence spectra (Supplementary Fig. 10a), which show that all charged exciton transitions appear on the low-energy side of X^0. Further investigation, both experimental and theoretical, would be needed to establish with certainty which excitonic transition is responsible for high-efficiency nuclear spin pumping in the regime of fast tunneling.

Supplementary Fig. 10c shows that nuclear spins can be polarized at photon energies down to $E_{\mathrm{Pump}} \approx 1.25$ eV, which is well below the ground state QD exciton energy and bulk GaAs bandgap. This mechanism leads to negative hyperfine shifts $E_{\mathrm{hf}} \approx -30$ μeV for both σ^+ and σ^- pumping, suggesting that optical excitation plays a different role, possibly related to activation of charge traps or Auger effect. The trapped-charge hypothesis is further supported by the bias dependence, which shows that the sub-bandgap nuclear spin pumping disappears for $V_{\mathrm{Pump}} < -0.5$ V and $V_{\mathrm{Pump}} > +0.9$ V. The buildup time of the nuclear spin polarization is found to be around ≈ 5 s, which is approximately an order of magnitude slower than nuclear spin pumping via resonant excitation of the QD excitons (see Supplementary Note 4 C). The exact mechanism of sub-bandgap optical nuclear spin pumping is currently unclear and would require a separate systematic investigation.

Focusing on the s-shell pumping, we plot the minimum E_{hf} (i.e. maximum $|E_{\mathrm{hf}}|$) as a function of bias V_{Pump} in Supplementary Fig. 11a. The corresponding photoluminescence intensity of the s-shell exciton under above-gap excitation is shown in Supplementary Fig. 11b. At positive V_{Gate} the electric field in the sample is small and the band structure is close to flat-band (right sketch in Supplementary Fig. 11c). As a result, optical recombination is the only way the photo-generated carriers can leave the QD. The typical radiative lifetimes for the studied type of GaAs QDs are

≈ 300 ps, which creates a bottleneck for how quickly the spin-polarized electrons can be injected into the QD, limiting in turn the rate of the nuclear spin pumping. When the sample gate is tuned towards larger reverse (negative) bias, the maximum hyperfine shift $|E_{\mathrm{hf}}|$ increases. At the same time, photoluminescence intensity gradually decreases when $V_{\mathrm{Gate}} < -1$ V, indicating that electrons and holes tunnel out of the QD (left sketch in Supplementary Fig. 11c) faster than they can recombine optically. We attribute this correlation to the key role that the tunneling plays in dynamic nuclear spin polarization. Fast tunneling overcomes the radiative-recombination bottleneck, so that high-power optical excitation can be used to inject spin polarized electrons at a high rate. The efficiency of nuclear spin pumping reaches its maximum at $V_{\mathrm{Pump}} = -2.3$ V. For even larger reverse bias (i.e. more negative V_{Pump}) the maximum hyperfine shift $|E_{\mathrm{hf}}|$ is seen to reduce slightly. For $V_{\mathrm{Pump}} < -2.3$ V tunneling becomes even faster, which would require an even higher pump power $P_{\mathrm{Pump}} \gtrsim 10$ mW to maintain steady-state occupation of the QD with spin-polarized electrons. However, when focused into a diffraction-limited spot, such high-power optical excitation causes heating of the crystal lattice, which may result in accelerated nuclear spin relaxation, explaining why the highest achievable nuclear spin polarization is reduced at very large reverse biases.

B. Estimate of the electron tunneling rate

In order to quantify the optical nuclear spin pumping process, we estimate the electron tunneling rate using the photoluminescence intensity data. We employ a rate equation approach by considering the probability p_{eh} that the QD s-shell is occupied by an electron-hole pair. Since we are only interested in an order of magnitude estimate, we ignore the contributions of the biexcitons (two electron-hole pairs) and charged excitons (an electron-hole pair with extra one electron or one hole). The steady state is defined by the balance between the rate of the optical excitation (Γ_{Exc}) and the rates of deexcitation through optical recombination (Γ_{R}) and tunneling ionization (Γ_{Tun}):

$$\begin{aligned} &(1 - p_{\mathrm{eh}})\Gamma_{\mathrm{exc}} - p_{\mathrm{eh}}(\Gamma_{\mathrm{R}} + \Gamma_{\mathrm{Tun}}) = 0 \Rightarrow \\ &p_{\mathrm{eh}} = \Gamma_{\mathrm{exc}}/(\Gamma_{\mathrm{exc}} + \Gamma_{\mathrm{R}} + \Gamma_{\mathrm{Tun}}) \end{aligned} \tag{S8}$$

The intensity of photoluminescence I_{PL} is proportional to p_{eh}. We further assume that the maximum observed PL intensity $I_{\mathrm{PL,max}}$ corresponds to a regime where the tunneling rate is negligible $\Gamma_{\mathrm{Tun}} \to 0$. With this assumption we eliminate the unknown PL intensity that would be observed

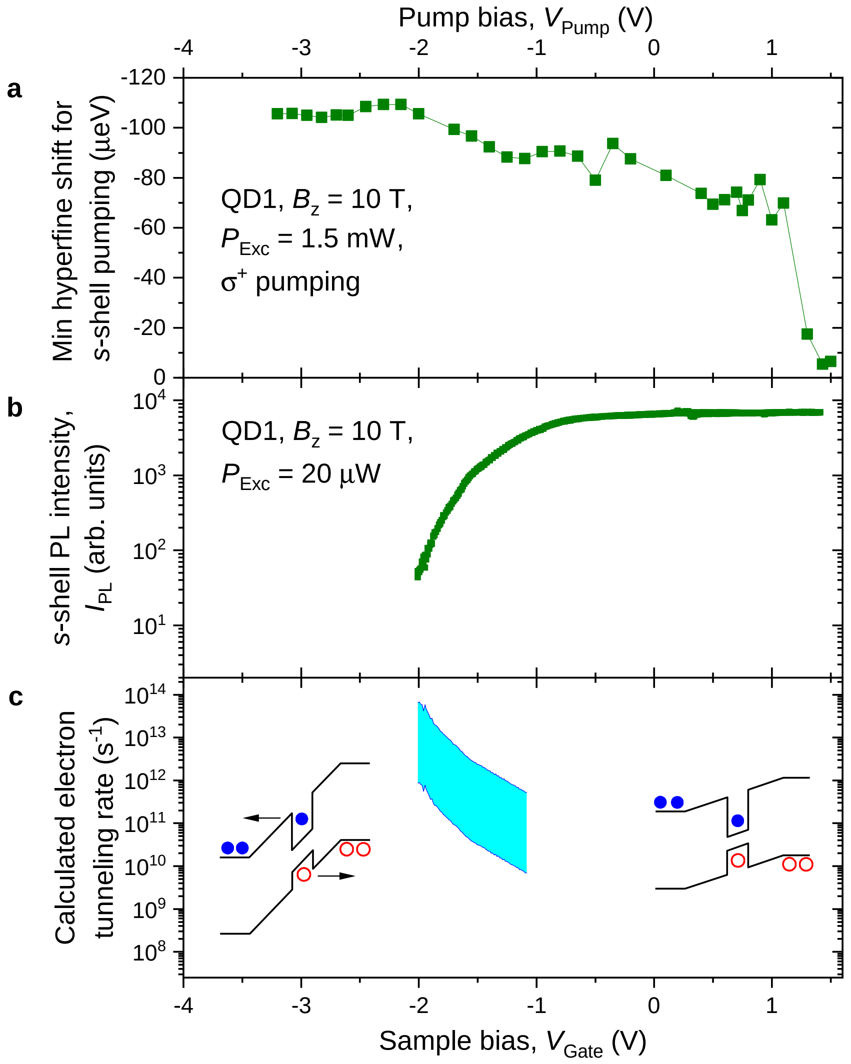

Supplementary Figure 11. **Bias dependence of nuclear spin pumping via s-shell excitation. a,** Steady-state hyperfine shift measured as a function of the pump bias V_{Pump} for pump photon energy E_{Pump} tuned into resonance with the s-shell exciton at each bias. Measurement is conducted on QD1 at $B_{\mathrm{z}} = 10$ T. **b,** Photoluminescence intensity of the s-shell exciton measured as a function of bias V_{Gate} under 632.8 nm high-power saturation. **c,** Calculated rate for the electron to tunnel out of the QD, shown as a shaded area between lower and upper bound estimates. Sketches show conduction and valence band profiles at positive and negative V_{Gate}.

at $p_{\mathrm{eh}} = 1$ and find for the tunneling rate at an arbitrary bias:

$$\Gamma_{\mathrm{Tun}} = (\Gamma_{\mathrm{R}} + \Gamma_{\mathrm{exc}}) \left(\frac{I_{\mathrm{PL,max}}}{I_{\mathrm{PL}}} - 1 \right) \tag{S9}$$

It then follows that the reduction of the photoluminescence intensity I_{PL} under reverse bias signifies that the tunneling rate exceeds the sum $(\Gamma_{\mathrm{R}} + \Gamma_{\mathrm{exc}})$ of the radiative recombination and optical excitation rates. The radiative recombination time is $1/\Gamma_{\mathrm{R}} \approx 300$ ps [25] for the studied type of

QDs ($\Gamma_{\mathrm{R}} \approx 3.3 \times 10^9$ s^{-1}). Since photoluminescence intensity of the s-shell exciton is saturated, we assume that the excitation rate exceeds the recombination rate $\Gamma_{\mathrm{Exc}} > \Gamma_{\mathrm{R}}$. This gives the lower bound estimate for the tunneling rate. The photoluminescence measurement shown in Supplementary Fig. 11b was conducted at high excitation power, exceeding the ground state exciton saturation power by a factor of ≈ 150. Thus we write $\Gamma_{\mathrm{Exc}} \lesssim 150\Gamma_{\mathrm{R}}$, which gives an upper bound estimate, since some of the photo-excited electron-hole pairs can recombine from higher shells, without reaching the s-shell. Using Supplementary Eq. S9 we calculate Γ_{Tun} taking I_{PL} and the maximum observed intensity $I_{\mathrm{PL,max}}$ from Supplementary Fig. 11b. The range between the lower bound ($\Gamma_{\mathrm{Exc}} = \Gamma_{\mathrm{R}}$) and the upper bound ($\Gamma_{\mathrm{Exc}} = 150\Gamma_{\mathrm{R}}$) estimates is shown by the shaded area in Supplementary Fig. 11c. Such direct evaluation of Γ_{Tun} is possible only when the bias is high enough to detect photoluminescence, but also low enough to have a substantial reduction of I_{PL} compared to $I_{\mathrm{PL,max}}$. These estimates show that the tunneling time at $V_{\mathrm{Gate}} = -2$ V is on the order of $\approx 0.1 - 1$ ps.

In order to estimate Γ_{Tun} below $V_{\mathrm{Gate}} < -2$ V we consider the well-known WKB approximation of the tunneling rate through a triangular barrier (see e.g. Ref. [26]). Up to a constant factor, we have:

$$\Gamma_{\mathrm{Tun}} \propto \exp\left(-\frac{4\sqrt{2m_{\mathrm{e}}}}{\hbar e F_z}|\epsilon_{\mathrm{e}}|^{3/2}\right), \tag{S10}$$

where m_{e}^* is the effective electron mass, ϵ_{e} is the electron ionization energy and F_z is the electric field in the growth direction. The total thickness of the structure between the doped layers is ≈ 300 nm, so the electric field is estimated as $F_z = -(V_{\mathrm{Gate}} - V_{\mathrm{Gate,0}})/300$ nm, where $V_{\mathrm{Gate,0}} = +1.55$ V is the built-in potential of the structure, deduced as the bias at which the PL Stark shift vanishes. Based on Ref. [27] we estimate the conduction band discontinuity between GaAs and $Al_{0.33}Ga_{0.67}As$ to be $U_{\mathrm{e}} \approx 0.28$ eV and take $m_{\mathrm{e}}^* = 0.067m_{\mathrm{e}}$, where m_{e} is the free electron mass. The s-shell photoluminescence of the QD is observed at ≈ 0.055 eV above the bulk GaAs. Taking that 0.63 of this offset is in the conduction band [27], we estimate the ionization energy $\epsilon_{\mathrm{e}} = U_{\mathrm{e}} - 0.63 \times (0.055 \text{ eV}) \approx 0.25$ eV. Substituting this, we find the numerical estimate $\Gamma_{\mathrm{Tun}} \propto \exp\left(\frac{64.2}{V_{\mathrm{Gate}}-1.55}\right)$, where V_{Gate} is in the units of Volts. Firstly, we see that the exponent is far from saturation in the studied range of V_{Gate}, so that further reduction of V_{Gate} (i.e. making the bias more negative) would result in shortened tunneling times. Secondly, going from $V_{\mathrm{Gate}} = -2$ V, where photoluminescence is measurable, to $V_{\mathrm{Gate}} \in [-2.7, -2.3]$ V, where dynamical nuclear spin polarization is most efficient, results in an order of magnitude higher Γ_{Tun}. Therefore we estimate $\Gamma_{\mathrm{Tun}} \approx 10^{13} - 10^{14}$ s^{-1} for optimal nuclear spin pumping (tunneling time $\approx 0.01 - 0.1$ ps).

We can now independently estimate the optical excitation rate that leads to optimal nuclear spin pumping. Resonance fluorescence intensity, measured on InGaAs/GaAs QDs in the same setup and under similar experimental conditions, saturates at $P_{\mathrm{Exc}} \approx 5$ nW. We assume that $\Gamma_{\mathrm{Exc}} \approx \Gamma_{\mathrm{R}}$ at saturation, where the radiative rate is $\Gamma_{\mathrm{R}} \approx 10^9$ s^{-1} for InGaAs QDs. Assuming that the excitation rate scales linearly with optical power, we find that the resonant pumping power of $P_{\mathrm{Pump}} \approx 1.5$ mW, used for optimal nuclear spin pumping, corresponds to $\Gamma_{\mathrm{Exc}} \approx \frac{1.5\ \mathrm{mW}}{5\ \mathrm{nW}} 10^9\ \mathrm{s}^{-1} \approx 3 \times 10^{14}\ \mathrm{s}^{-1}$. This corresponds to optical reexcitation time of ≈ 0.0033 ps. These estimates yield Γ_{Exc} that are comparable to or somewhat higher than the above-calculated Γ_{Tun}, as would be expected for a steady-state condition. In other words, having $\Gamma_{\mathrm{Exc}} \gtrsim \Gamma_{\mathrm{Tun}}$ ensures that optical excitation generates as many spin-polarized electrons per unit of time as permitted by the rate of the tunnel escape. The typical linewidths of the excitonic shell peaks in the spectra of the steady state E_{hf} at large reverse biases (Supplementary Figs. 4, 10c) are on the order of ≈ 10 meV, which translates to ≈ 0.4 ps, corroborating our order-of-magnitude estimates of Γ_{Tun}.

Apart from the fast cycling of the optically-generated electrons, we expect that fast tunneling also facilitates the nuclear spin pumping by disrupting the formation of coherent nuclear "dark" states, which are otherwise predicted to prevent the approach to a near-unity nuclear spin polarization [28, 29]. Indeed, recent studies have shown that fluctuations of the electron spin reduce the coherence of the nuclear spins in a QD [30]. Moreover, the short (tunneling-limited) lifetime of the electron spin can be interpreted as spectral broadening of the electron spin levels. Such spectral broadening can facilitate nuclear spin pumping by compensating the energy mismatch of the electron and nuclear spin Zeeman energies, which otherwise inhibits the electron-nuclear spin flip-flops. The role played by tunneling is then similar to the effect that elevated lattice temperatures have on nuclear spin polarization, as studied previously in InGaAs QDs [24]. Future theoretical work may explain the details of the nuclear spin pumping process by treating optical excitation, tunneling and electron-nuclear spin interactions in a unified framework.

C. Nuclear spin buildup dynamics

The nuclear spin buildup dynamics under optical pumping are non-exponential (Fig. 4a of the main text). The exact description of nuclear spin buildup dynamics is complicated due to the multitude of the contributing factors, including spin diffusion [15] and the inhomogeneity of the hyperfine coupling between the nuclei and the localized QD electron. Precise description of the buildup dynamics is beyond the scope of this work. Therefore, we limit the analysis to an empirical

fit of the data by a sum of two stretched exponentials:

$$E_{\rm hf} = E_{\rm hf,fast}\,(1-\exp(-(t/\tau_{\rm fast})^{\kappa_{\rm fast}})) + E_{\rm hf,slow}\,(1-\exp(-(t/\tau_{\rm slow})^{\kappa_{\rm slow}}))\,. \tag{S11}$$

The best fits are shown by the solid lines in Fig. 4a of the main text and are seen to capture well the strongly non-exponential buildup dynamics. The fitting parameters for the optimal steady-state nuclear spin pumping of QD2 at $B_z = 10$ T are as follows:

Parameter	σ^+ pumping	σ^- pumping
$\tau_{\rm fast}$	0.1215 s	0.2446 s
$\tau_{\rm slow}$	0.5174 s	1.1903 s
$E_{\rm hf,fast}$	-43.03 μeV	69.46 μeV
$E_{\rm hf,slow}$	-67.51 μeV	41.44 μeV
$\kappa_{\rm fast}$	0.88	0.68
$\kappa_{\rm slow}$	0.44	0.36

(S12)

Due to the empirical nature of the fitting model, its parameters should be treated as estimates. Nevertheless, we can establish that the fast initial buildup occurs on a $0.1-0.3$ s timescale, slowing down to $0.5-1.5$ s when the nuclear spin polarization approaches closer to its steady state. The fast initial buildup is likely dominated by the nuclear spins located at the center of the QD, and therefore characterizes the inherent nuclear spin pumping rate. The slower dynamics at the later stage are likely dominated by the nuclei that are further away from the QD center. This slower component is a result of a complex interplay between several competing processes. On the one hand, these distant nuclei are polarized both directly by the electron and indirectly through spin diffusion from the center of the QD. On the other hand, spin diffusion from these nuclei further into the barriers also causes the depolarization.

Electron-nuclear spin dynamics become nonlinear when electron spin Zeeman splitting is cancelled by the hyperfine shift. This is manifested in a kink in the nuclear spin buildup dynamics, observed at $E_{\rm hf} \approx +50$ μeV in Fig. 4a of the main text. From the zero-splitting condition $E_{\rm hf} \approx -\mu_{\rm B} g_{\rm e,z} B_{\rm z}$ we can estimate the electron g-factor $g_{\rm e,z} \approx -0.09$, in agreement with previous measurements on the same structure [15].

It is also interesting to estimate the rate of the electron-nuclear spin flip-flops. Starting from a depolarized state, it takes IN spin flips to achieve a fully polarized state of $N \approx 10^5$ nuclei with spin I. The hyperfine shift $E_{\rm hf}$ corresponding to a fully polarized state is $IF_{\rm tot} \approx 110-115$ μeV. On the other hand, from the nuclear spin buildup dynamics measurements we find that the highest rate of change in $E_{\rm hf}$ (the derivative at the start of pumping from a depolarized state) is ≈ 600 μeV/s, in

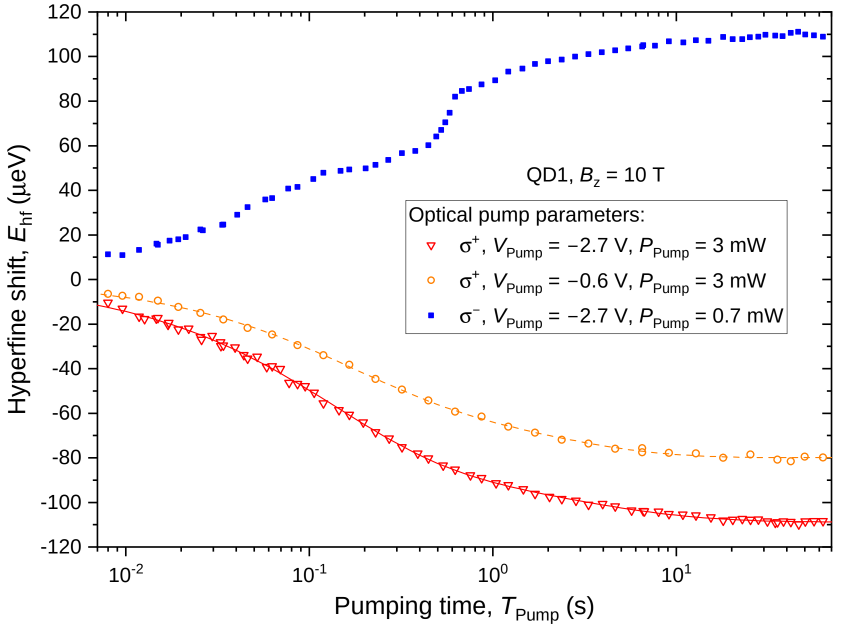

Supplementary Figure 12. **Nuclear spin buildup dynamics.** Symbols show nuclear spin buildup dynamics measured for an individual dot QD1 at $B_z = 10$ T under σ^- (squares) and σ^+ (triangles, circles) optical pumping. Optical pump photon energy is tuned into the s-shell resonance at pump bias $V_{\mathrm{Pump}} = -2.7$ V (squares, triangles) or $V_{\mathrm{Pump}} = -0.6$ V (circles). Lines show biexponential fitting.

agreement with τ_{fast} derived above. Combining these parameters we estimate the electron-nuclear flip-flop rate to be $\approx 8 \times 10^5$ s^{-1} (i.e. one nucleus flipped every ≈ 1 μs). Assuming that the cycling of the spin-polarized electrons is limited by the tunneling rate of $\Gamma_{\mathrm{Tun}} \approx 10^{13}$ s^{-1}, we estimate that only a small fraction $\approx 10^{-7}$ of the injected electrons transfer their spin to the nuclei, while the rest tunnel out of the QD without polarizing the nuclear spins.

Supplementary Fig. 12 shows the buildup dynamics in another individual quantum dot (QD1). Here, the pump bias is set to either the optimal value of $V_{\mathrm{Pump}} = -2.7$ V (squares, triangles) or a suboptimal value of $V_{\mathrm{Pump}} = -0.6$ V (circles), and the photon energy of the optical pump is tuned into the s-shell resonance at each pump bias. The best fits for σ^+ pumping are shown by the lines.

The fitting parameters for QD1 at $B_z = 10$ T are as follows:

Parameter	$\sigma^-, V_{\text{Pump}} = -0.6$ V	$\sigma^+, V_{\text{Pump}} = -2.7$ V
τ_{fast}	0.1391 s	0.1228 s
τ_{slow}	1.325 s	1.5697 s
$E_{\text{hf,fast}}$	-44.13 μeV	-71.37 μeV
$E_{\text{hf,slow}}$	-35.80 μeV	-37.39 μeV
κ_{fast}	0.72	0.69
κ_{slow}	0.58	0.50

(S13)

The fitted buildup times for QD1 are similar to those found for QD2 (Supplementary Eq. S12). Moreover, although the steady-state nuclear polarization is lower at the suboptimal bias of $V_{\text{Pump}} = -0.6$ V, the buildup times are nearly the same as at the optimal bias $V_{\text{Pump}} = -2.7$ V where the steady-state nuclear polarization is maximized. This is rather unexpected and is in contrast to the tunneling rate dependence, which is seen to change by orders of magnitudes between $V_{\text{Pump}} = -2.7$ V and -0.6 V (Supplementary Fig. 11c). This indicates that the change in the cycling rate of the spin-polarized electrons is not the only effect of the bias. The alternative effects of a large reverse bias, discussed above, may include the relaxation of the coherent-dark-state bottleneck, and the bridging of the electron-nuclear energy gap by the tunneling-broadened electron spin states. There are further effects of the large reverse bias which may explain why the largest nuclear polarization is observed at $V_{\text{Pump}} = -2.7$ V, even though the tunneling escape rate is already sufficiently fast at $V_{\text{Pump}} = -0.6$ V. For example, the large electric field, produced by the reverse bias, changes the composition of the valence band in terms of the heavy and light hole contributions [31], which in turn may change the purity of the electron spin polarization produced by the elliptically polarized pump light. A large electric field may also increase the spatial spread of the electron envelope wavefunction, helping to polarize the nuclear spins that are further away from the center of the QD. These considerations highlight the considerable complexity of the processes responsible for cooling of a many-body nuclear spin ensemble. Our demonstration of experimental conditions that lead to a large nuclear polarizations is an important starting point for future work which would be needed to unravel this complexity.

D. Relation between nuclear spin cooling and state narrowing

Here we compare nuclear spin polarization, described in this work, to the class of "state narrowing" techniques [32–34], which are sometimes also described as spin cooling. The simplest way

to describe the difference is to note that dynamic nuclear polarization with $|P_{\mathrm{N}}| \to 1$ corresponds to the true cooling of the nuclear spin ensemble, whereas the narrowing schemes can be described as partial cooling of certain degrees of freedom, while leaving the remaining degrees of freedom of the nuclear ensemble in a "hot" state. In order to explain this in more detail, we refer to Figs. 4d,e of the main text. Fig. 4d of the main text sketches the density of states calculated for $N = 6$ dipolar-coupled $I = 3/2$ nuclei (without the electron). At high magnetic fields the density of state spectrum consists of well resolved bands. Each individual band corresponds to a well-defined total spin projection M. The adjacent bands are split by the Zeeman energy $h\nu_{\mathrm{L}}$, where the Larmor frequency is in the $\nu_{\mathrm{L}} \approx 30 - 100$ MHz range in our experiments. The transition between any two adjacent bands can be described as flipping (i.e. a ± 1 change in the z-projection m) of a single spin in the ensemble. Without the nuclear-nuclear interaction, each band would represent a degenerate state. The dipole-dipole interaction between the nuclei lifts the degeneracy, broadening each band. The broadening of each band is governed by the magnitude of the nuclear-nuclear dipolar couplings $h\nu_{\mathrm{dd}} \propto \max |b_{j,k}|$ and is on the order of $\nu_{\mathrm{dd}} \approx 1$ kHz in GaAs based structures. The origin of this broadening can be understood as follows. There are a large number of combinations of the individual nuclear states that result in a given total polarization M. However, each such combination (microstate) has a slightly different energy depending on the mutual orientation of the adjacent nuclei in the ensemble. For $M \approx 0$ (i.e. for small nuclear spin polarization $P_{\mathrm{N}} \approx 0$) the broadening is maximal and the number of microstates is large, meaning that such bands have a quasicontinuous spectrum. With the increasing polarization degree P_{N} the number of possible microscopic configuration reduces and the distribution of the available dipolar energies becomes narrower. There are only two non-degenerate many-body eigenstates, achieved in the ultimate limit of a fully-polarized ensemble (i.e. at $M = \pm NI$, $P_{\mathrm{N}} = \pm 1$). Notably, these fully-polarized states are separated by a large Zeeman energy gap $h\nu_{\mathrm{L}}$ from all the other states many-body states.

The difference between polarization and state narrowing is illustrated in Fig. 4e of the main text. In case of state narrowing, the degree of freedom that is cooled down is the distribution of the total z-projection M of the nuclear spins (blue bars show Gaussian distribution of M with a full width of half maximum of 2). In an ideal case, one aims to narrow the distribution down to just one value of M. However, even for such ultimate narrowing the dipolar energy reservoir remains in a "hot" state, in a sense that a fixed M can be implemented with equal probability by any of the large number of microstates. In case of a polarized state (true cooling), the probability to find the ensemble in a microstate with a total projection M approximately follows the Boltzmann distribution $\propto \exp(\beta M)$, where β is the dimensionless inverse spin temperature. The probabilities

of the microstates are shown by the red bars in Fig. 4e for a polarized ensemble at $P_{\mathrm{N}} = 0.99$. It can be seen that true cooling (polarization) both narrows the distribution M and narrows the dipolar energy distribution. This is a fundamental advantage of true cooling via polarization.

Due to the difference between polarization and state narrowing the figures of merit are also different. One metric is the polarization degree P_{N}. Although state narrowing protocols are primarily designed to reduce the uncertainty in M, they can be tailored to produce a non-zero mean M. However, the resulting polarization degrees are modest, for example $P_{\mathrm{N}} \approx 0.3$ in a recent demonstration [34]. The likely limiting factor is the nuclear spin polarization rate, arising from the need to have a coherent electron spin qubit in the QD. The polarization protocol reported here benefits from operating the QD in a regime of fast tunneling, which allows much high polarizations $|P_{\mathrm{N}}| \geq 0.95$ to be achieved.

Another figure of merit is the uncertainty in M. Recent algorithmic cooling experiments [34] reported an uncertainty of 5.7. In case of polarization, currently there is no measurement of uncertainty in M. Such measurement would require additional experiments, for example a measurement of the electron spin qubit dephasing. However, we can calculate the uncertainty in M from the first principles. To this end we describe the state of the nuclear spin ensemble in terms of probabilities p_m for each nuclear spin to occupy a state with spin z projections m. In case of the spin-3/2 nuclei $m \in \{-3/2, -1/2, +1/2, +1/2\}$. For the state induced by optical dynamical nuclear polarization we model these probabilities using the Boltzmann distribution:

$$p_m = e^{m\beta} / \sum_{m=-I}^{+I} e^{m\beta}, \tag{S14}$$

where $\beta = h\nu_{\mathrm{L}}/k_{\mathrm{b}}T_{\mathrm{N}}$ is the dimensionless inverse temperature, expressed in terms of the nuclear spin Larmor frequency ν_{L} and the spin temperature T_{N} (h is Planck's constant and k_{b} is the Boltzmann constant). We then consider an ensemble of N identical spins, assuming that their states follow the Boltzmann distribution independently. We are interested in the random variable M, which is the total of the spin projections m of the individual spins. We calculate the variance of M. The details of the calculations, carried out using Wolfram Mathematica software, can be found in the Supplementary file `SupplementaryData1.nb` (pdf version of this file can be found in `SupplementaryData2.pdf`). The variance can be written in terms of the hyperbolic cosecant functions:

$$V(M) = \frac{N}{4}\left(\mathrm{csch}^2[\beta/2] - (2I+1)^2\mathrm{csch}^2[(I+1/2)\beta]\right), \tag{S15}$$

We consider a QD with $N \approx 10^5$ nuclei with spin $I = 3/2$. For an unpolarized state ($P_{\mathrm{N}} \approx 0$)

the standard deviation uncertainty is $\sqrt{V(M)} \approx 354$. For a conservative estimate of $P_\mathrm{N} \approx 0.95$ ($\beta \approx 2.66$) in the best QDs we find $\sqrt{V(M)} \approx 90$. For $P_\mathrm{N} \approx 0.99$ ($\beta \approx 4.2$), the uncertainty in M reduces to $\sqrt{V(M)} \approx 39$. This is still worse than the state-of-the-art performance of the state narrowing techniques. However, our assumption of independent spins could be leading to an overestimate. Usually nuclear spin pumping processes result in some additional narrowing of M, for example through electron-nuclear spin feedback [6]. Investigation of nuclear spin statistics beyond the measurement of P_N is a subject for future work.

Overall, the polarization protocol implemented in our work and the state narrowing techniques use rather different approaches, with their advantages and disadvantages. The state narrowing methods offer a reliable reduction of M, which is key to reducing the QD electron spin qubit dephasing time $T^*_{2,\mathrm{e}}$. Yet, even in an ideal case, where M is known down to one unit of spin, such a narrowed state still has a large statistical uncertainty associated with different mutual orientations of the nuclear spins. When the electron spin qubit is added into consideration, such uncertainty in the dipolar energy also translates into uncertainty of the effective nuclear field acting on the electron (due to the inhomogeneity of the hyperfine coupling of the localized electron). The quasicontinuous distribution of the dipolar energies for any given M opens the channels for electron-nuclear spin dynamics which can reduce the electron spin coherence time $T_{2,\mathrm{e}}$. Our approach of cooling through polarization requires further investigation and improvement to be comparable with state narrowing. However, true cooling has a unique fundamental advantage of being the only route towards a ground eigenstate of a many-body nuclear spin ensemble. Since such a ground state is non-degenerate and is spectrally isolated by a large energy gap $h\nu_\mathrm{L}$ it results in essentially nondecaying electron spin coherence, as shown through exact analytical solution [35]. For practical applications one may envisage combining the true cooling and the state narrowing into a two-stage process with the aim of achieving new levels of control over the nuclear spin ensemble state.

Supplementary Note 5. DERIVATION OF NUCLEAR SPIN POLARIZATION

A. Nuclear magnetic resonance thermometry of spin-3/2 nuclei

We use again the model of Supplementary Eq. S14. We assume $\nu_\mathrm{L} > 0$ and $B_\mathrm{z} > 0$, so that $m = +I$ is the ground state for nuclei with $\gamma > 0$, in agreement with Supplementary Eq. S3. For spin $I = 1/2$ where $m = \pm 1/2$ any statistical distribution is described by Supplementary Eq. S14 with some T_N. By contrast, for $I > 1/2$ Supplementary Eq. S14 states the non-trivial nuclear

spin temperature hypothesis [36] – previous experimental studies on low-strain epitaxial quantum dots [13] have shown its validity for the state induced by optical dynamical nuclear polarization. Nuclear spin polarization degree is defined as

$$P_{\mathrm{N}} = \frac{1}{I}\sum_{m=-I}^{+I} m p_m. \tag{S16}$$

For the Boltzmann distribution of Supplementary Eq. S14 the polarization degree is given by the Brillouin function:

$$P_{\mathrm{N}} = \frac{1}{2I}\left((2I+1)\coth[(I+1/2)\beta] - \coth[\beta/2]\right). \tag{S17}$$

It is worth noting that the polarization degree P_{N} and the dimensionless inverse temperature β provide more relevant description of the nuclear spin state than the temperature T_{N}. Indeed, when the Larmor frequency ν_L is changed (by varying the external magnetic field) P_{N} and β are preserved, whereas T_{N} is not constant, even if it is the same optically-pumped nuclear spin state. The temperature T_{N} only gains physical meaning at low magnetic fields $\lesssim 1$ mT, comparable to the local nuclear dipolar fields. P_{N} and β are also related to entropy [37, 38]. For N spin-1/2 nuclei the entropy reads:

$$\frac{S}{Nk_{\mathrm{B}}} \approx \ln 2 - \frac{1}{2}\left[(1+P_{\mathrm{N}})\ln(1+P_{\mathrm{N}}) + (1-P_{\mathrm{N}})\ln(1-P_{\mathrm{N}})\right]. \tag{S18}$$

For $P_{\mathrm{N}} \to 1$ this can be approximated as

$$\frac{S}{Nk_{\mathrm{B}}} \approx \frac{1-P_{\mathrm{N}}}{2}(1+\ln 2 - \ln(1-P_{\mathrm{N}})) = \frac{1-P_{\mathrm{N}}}{2}\left(1 - \ln\left(\frac{1-P_{\mathrm{N}}}{2}\right)\right). \tag{S19}$$

The dependence is plotted by the solid line in Supplementary Fig. 13 and is very nonlinear. A significant reduction in the entropy (i.e. reduction in disorder) requires P_{N} to be close to unity. For example, at $P_{\mathrm{N}} \approx 0.8$ the entropy is reduced only to $\approx 1/2$ of its maximum value at $P_{\mathrm{N}} = 0$, while a factor of 10 reduction requires $P_{\mathrm{N}} \approx 0.974$. The minimum in entropy is achieved only for $P_{\mathrm{N}} = \pm 1$ ($\beta \to \pm\infty$). An alternative way to characterize the polarization degree of a nuclear spin ensemble is through the reduction of the dephasing time of the central (electron) spin qubit coupled to such an ensemble. Under certain approximations, the dephasing rate can be shown to scale as $\sqrt{1-P_{\mathrm{N}}^2}$ with nuclear spin polarization degree [39]. This is shown by the dashed line in Supplementary Fig. 13, once again demonstrating that nuclear polarization must be close to unity in order to make a difference in terms of the central spin coherence.

The hyperfine shift experienced by the quantum dot exciton is linearly proportional to the nuclear spin polarization degree P_{N}:

$$E_{\mathrm{hf}} = \sum_j F^{(j)} I_j P_{\mathrm{N},j}, \tag{S20}$$

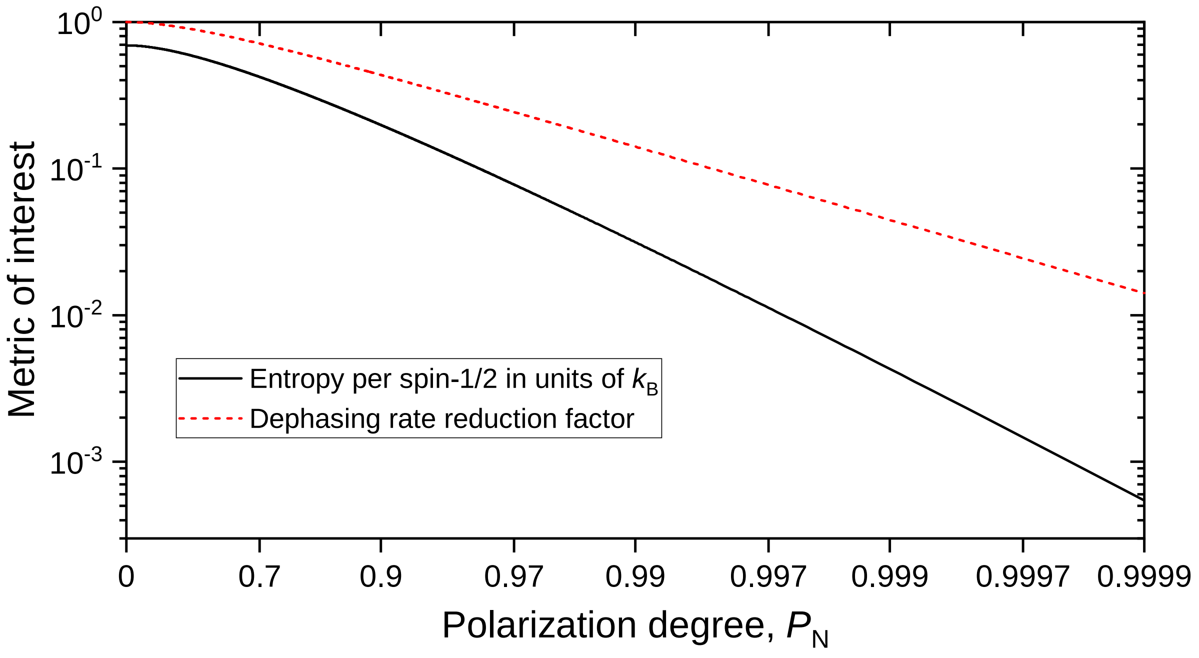

Supplementary Figure 13. **Nuclear spin ensemble properties as a function of nuclear spin polarization degree P_{N}.** Solid line shows entropy per nucleus of a nuclear spin ensemble. Dashed line shows the factor by which the dephasing time of an electron spin qubit is reduced, when its nuclear spin environment is polarized.

where the sum is over individual isotope species. Although the hyperfine shift E_{hf} can be measured accurately from the photoluminescence spectra, the proportionality factor $F^{(j)}$ depends not only on the material's hyperfine constants $A^{(j)}$, but also on the leakage of the electron wavefunction into the AlGaAs barrier. Since it is difficult to estimate this leakage independently, the measurement of E_{hf} alone is not suitable for accurate derivation of P_{N}. The unknown proportionality factor between E_{hf} and P_{N} can be eliminated for $I > 1/2$ nuclei, provided that it is possible to address selectively the magnetic dipole transitions between states with spin projections m and $m+1$. For example, if a long radiofrequency (Rf) pulse is applied to saturate the $m \leftrightarrow m+1$ NMR transition, it equalizes the populations of these states. The resulting final population probabilities p_m, p_{m+1} both equal the average $(p_m + p_{m+1})/2$ of their initial populations. For an ideal selective NMR Rf excitation the population probabilities of all other nuclear spin states remain unchanged.

One can then substitute Supplementary Eq. S16 into Supplementary Eq. S20 to calculate the change in the optically detected hyperfine shift ΔE_{hf} resulting from selective saturation of a single NMR transition $m \leftrightarrow m+1$. For example, for $+1/2 \leftrightarrow +3/2$ of the j-th isotope, we calculate $\Delta E_{\mathrm{hf}}^{+1/2\leftrightarrow+3/2} = F^{(j)}\left[(+\frac{3}{2})\frac{p_{+3/2}+p_{+1/2}}{2} + (+\frac{1}{2})\frac{p_{+3/2}+p_{+1/2}}{2}\right] - F^{(j)}\left[(+\frac{3}{2})p_{+3/2} + (+\frac{1}{2})p_{+1/2}\right] =$

$-F^{(j)}(p_{+3/2} - p_{+1/2})/2$. This result has a simple interpretation that the hyperfine shift variation $\Delta E_{\rm hf}$ depends only on the difference in the initial populations of the states that are selectively saturated with Rf.

In the same manner, simultaneous selective saturation of the NMR transitions $m \leftrightarrow m+1$ and $m+1 \leftrightarrow m+2$ leads to complete averaging of the populations of the three involved spin states. Their final population probabilities become $p_m, p_{m+1}, p_{m+2} \rightarrow (p_m + p_{m+1} + p_{m+2})/3$. Saturation of all three NMR transitions of spin $I = 3/2$ nuclei leads to complete depolarization and equal populations of all four spin states $p_{-3/2} = p_{-1/2} = p_{+1/2} = p_{+3/2} = 1/4$. Using Supplementary Eqs. S16 and S20 we evaluate the changes in the hyperfine shift $\Delta E_{{\rm hf},j}$ arising from the j-th isotope, to arrive to the following results, derived previously in Ref. [13]:

$$\begin{aligned}
\Delta E_{{\rm hf},j}^{m\leftrightarrow m+1} &= -F^{(j)}(p_{m+1,j} - p_{m,j})/2 = -F^{(j)}\frac{e^{(m+1)\beta_j} - e^{m\beta_j}}{4\cosh(\beta_j/2) + 4\cosh(3\beta_j/2)},\\
\Delta E_{{\rm hf},j}^{m\leftrightarrow m+2} &= -F^{(j)}(p_{m+2,j} - p_{m,j}) = -F^{(j)}e^{(m+1)\beta_j}\sinh(\beta_j/2)/\cosh(\beta_j),\\
\Delta E_{{\rm hf},j}^{-I\leftrightarrow +I} &= -F^{(j)}P_{{\rm N},j}I_j = -F^{(j)}[3/2 + 1/\cosh(\beta_j)]\tanh(\beta_j/2).
\end{aligned} \tag{S21}$$

The last expression in each of these equations is obtained by substituting the Boltzmann distribution (Supplementary Eq. S14) for spin $I = 3/2$.

B. Corrections for the nuclei with small or inverted quadrupolar shifts

For a fully resolved NMR triplet, Supplementary Eq. S21 is sufficient to extract the inverse temperatures β_j and derive the polarization degree of the spin-3/2 nuclei. In a real semiconductor system the separation of the quadrupolar NMR components is not perfect. Here we examine the role that the nuclei with small or inverted quadrupolar shift $\nu_{\rm Q}$ have on the derivation of nuclear spin polarization from experimental data. The case of an experiment where a radiofrequency comb is used to saturate two out of three NMR transitions is considered in Supplementary Fig. 14. For unstrained GaAs, nuclear quadrupolar effects are absent ($\nu_{\rm Q} = 0$) and all NMR transitions of the spin-3/2 nucleus appear at the same Larmor frequency $\nu_{\rm L}$. Strain induces quadrupolar effects which are characterized to first order by the shift $\nu_{\rm Q}$. In all our experiments $|\nu_{\rm Q}| \ll |\nu_{\rm L}|$, so that first-order approximation is valid. The central transition $-1/2 \leftrightarrow +1/2$ between nuclear states with spin projections $m = \pm 1/2$ is unaffected by quadrupolar shifts in the first order, hence its NMR frequency is $\nu_{\rm L}$ for all nuclei (vertical solid line in Supplementary Fig. 14). The satellite transitions are affected by quadrupolar shifts: the NMR frequency of the $-3/2 \leftrightarrow -1/2$ transition is $\nu_{\rm L} + \nu_{\rm Q}$, whereas the NMR frequency of the $+1/2 \leftrightarrow +3/2$ transition is $\nu_{\rm L} - \nu_{\rm Q}$ (solid lines in

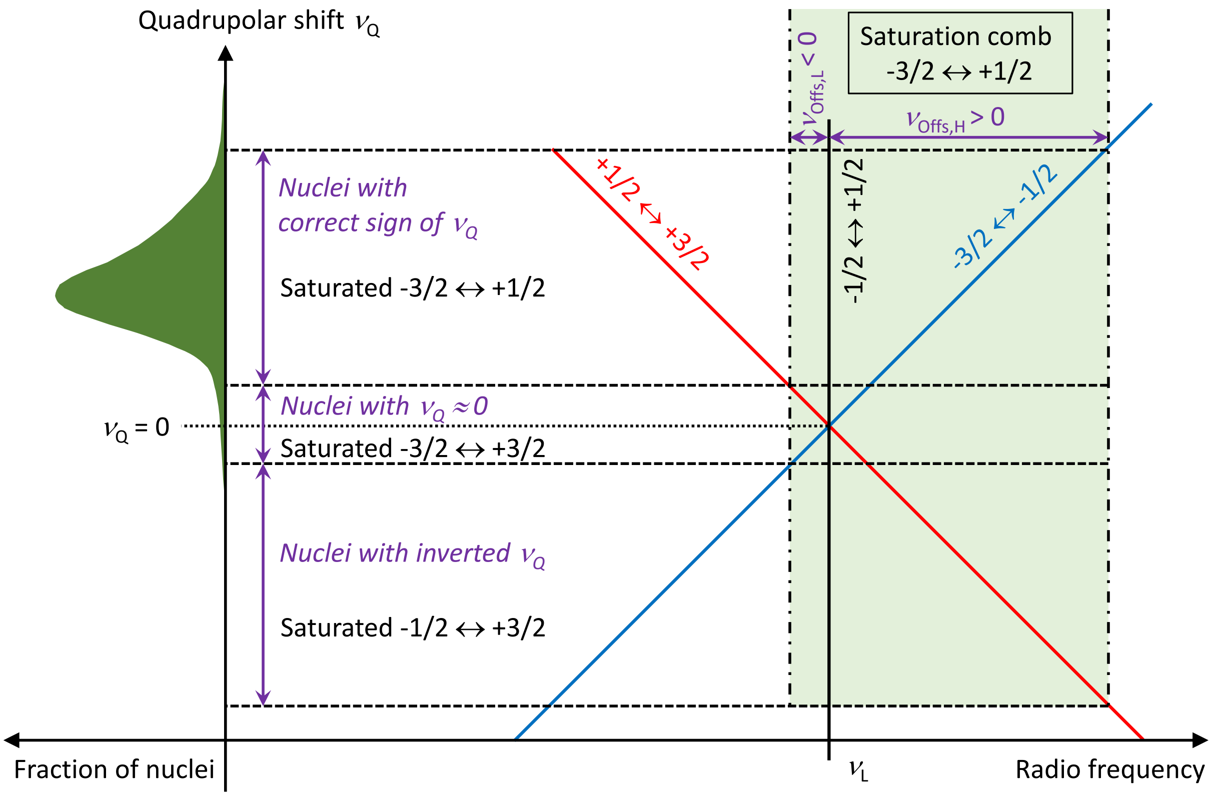

Supplementary Figure 14. **Effect of the nuclei with different quadrupolar shifts in a two-transition comb saturation measurement.**

Supplementary Fig. 14 with slopes +1 and −1, respectively).

The strain varies within the QD volume, so there is a statistical distribution of ν_Q values within the ensemble of the nuclei (sketched in the left part of Supplementary Fig. 14). The majority of the ^{75}As nuclei have a positive quadrupolar shift $\nu_Q > 0$ (for Ga nuclei the shift is predominantly negative $\nu_Q < 0$). Therefore, if we want to saturate simultaneously the two NMR transitions $-3/2 \leftrightarrow -1/2$ and $-1/2 \leftrightarrow +1/2$ (labelled $-3/2 \leftrightarrow +1/2$ for brevity) we choose a radiofrequency comb band sketched by the shaded area in Supplementary Fig. 14. The low-offset edge of the band at frequency $\nu_L + \nu_{Offs,L}$ (with negative $\nu_{Offs,L} < 0$) is tuned just below the Larmor frequency, in order to saturate the narrow $-1/2 \leftrightarrow +1/2$ transition. The high-offset edge of the band $\nu_L + \nu_{Offs,H}$ (with positive $\nu_{Offs,H} > 0$) is chosen to be far enough from the Larmor frequency to cover the $-3/2 \leftrightarrow -1/2$ satellite transition for most nuclei. The typical values in two-transition comb saturation experiments are $\nu_{Offs,L} = -5$ kHz, $\nu_{Offs,H} = +178$ kHz for ^{75}As and $\nu_{Offs,L} = +5$ kHz, $\nu_{Offs,H} = -80$ kHz for ^{69}Ga. For the $-1/2 \leftrightarrow +3/2$ two-transition saturation the values of $\nu_{Offs,L}$ and $\nu_{Offs,H}$ are inverted.

As can be seen in Supplementary Fig. 14 the nominal $-3/2 \leftrightarrow +1/2$ comb saturates the desired

transitions for the majority of nuclei, which have $-\nu_{\mathrm{Offs,L}} < \nu_{\mathrm{Q}} < \nu_{\mathrm{Offs,H}}$ (note that the lower bound $-\nu_{\mathrm{Offs,L}}$ is positive for a negative $\nu_{\mathrm{Offs,L}} < 0$). These nuclei give a correct contribution to the Rf-induced hyperfine shifts. But there are also several cases, where nuclei give contributions that differ from those intended. For a fraction of nuclei with small quadrupolar shifts $\nu_{\mathrm{Offs,L}} < \nu_{\mathrm{Q}} < -\nu_{\mathrm{Offs,L}}$ all three NMR transitions are excited by the comb, resulting in full depolarization of such nuclei. Furthermore, for those ^{75}As nuclei where quadrupolar shift is inverted $-\nu_{\mathrm{Offs,H}} < \nu_{\mathrm{Q}} < \nu_{\mathrm{Offs,L}}$, the $-3/2 \leftrightarrow -1/2$ transition will be out of resonance with the Rf band, while the $+1/2 \leftrightarrow +3/2$ transition will be saturated. Such nuclei will produce hyperfine shifts that would correspond to the $-1/2 \leftrightarrow +3/2$ two-transition saturation, rather than the intended $-3/2 \leftrightarrow +1/2$ saturation. Finally, for a small fraction of nuclei with very large absolute quadrupolar shifts $|\nu_{\mathrm{Q}}| > |\nu_{\mathrm{Offs,H}}|$, only the central $-1/2 \leftrightarrow +1/2$ transition will be saturated. Thus, introducing the empirical coefficients c, the observed hyperfine shift $\Delta E_{\mathrm{hf,Obs}}^{-3/2\leftrightarrow+1/2}$ in the $-3/2 \leftrightarrow +1/2$ two-transition saturation experiment can be written as:

$$\begin{aligned}\Delta E_{\mathrm{hf,Obs}}^{-3/2\leftrightarrow+1/2} &= c_{\mathrm{Sat,Ideal}}\Delta E_{\mathrm{hf}}^{-3/2\leftrightarrow+1/2} + \\ &+ c_{\mathrm{Sat,Full}}\Delta E_{\mathrm{hf}}^{-3/2\leftrightarrow+3/2} + c_{\mathrm{Sat,Inv}}\Delta E_{\mathrm{hf}}^{-1/2\leftrightarrow+3/2} + c_{\mathrm{Sat,CT}}\Delta E_{\mathrm{hf}}^{-1/2\leftrightarrow+1/2},\end{aligned} \tag{S22}$$

where we have dropped the isotope index. If the quadrupolar NMR triplet is fully resolved, then $c_{\mathrm{Sat,Ideal}} = 1$ with all other c coefficient equal to zero. In a real quantum dot $c_{\mathrm{Sat,Ideal}} < 1$ and the remaining coefficients are non-zero, so that the observed hyperfine shift $\Delta E_{\mathrm{hf,Obs}}^{-3/2\leftrightarrow+1/2}$ deviates from the ideal $\Delta E_{\mathrm{hf}}^{-3/2\leftrightarrow+1/2}$. For example, if all nuclei are in a $m = +3/2$ state (that is $P_{\mathrm{N}} = +1$), the expected ideal is $\Delta E_{\mathrm{hf}}^{-3/2\leftrightarrow+1/2} = 0$. In reality, due to the non-zero contributions of the fully-saturated nuclei ($c_{\mathrm{Sat,Full}} > 0$) and the nuclei with an inverted quadrupolar shift ($c_{\mathrm{Sat,Inv}} > 0$) the observed hyperfine shift $\Delta E_{\mathrm{hf,Obs}}^{-3/2\leftrightarrow+1/2}$ is non-zero even if nuclei are fully polarized.

The expression for the hyperfine shift in the other two-transition experiment $\Delta E_{\mathrm{hf,Obs}}^{-1/2\leftrightarrow+3/2}$ can be obtained from Supplementary Eq. S22 by changing the sings of all the m indices. It is worth noting that in the experiment with intentional saturation of all three-transitions ($-3/2 \leftrightarrow +3/2$) all nuclei get fully depolarized as long as the satellite transitions fit within the Rf band. In the three-transition experiments we use frequency combs with total widths of 347 kHz (^{69}Ga) and 578 kHz (^{75}As), centred at the Larmor frequency ν_{L}. These widths are sufficient for complete depolarization of essentially all the nuclei of the quantum dot and the surrounding barriers. Therefore, the three-transition non-selective saturation measurement is expected to be more robust than the selective two-transition Rf depolarization.

Similar analysis applies to selective Rf excitation of a single NMR transition (Supplementary

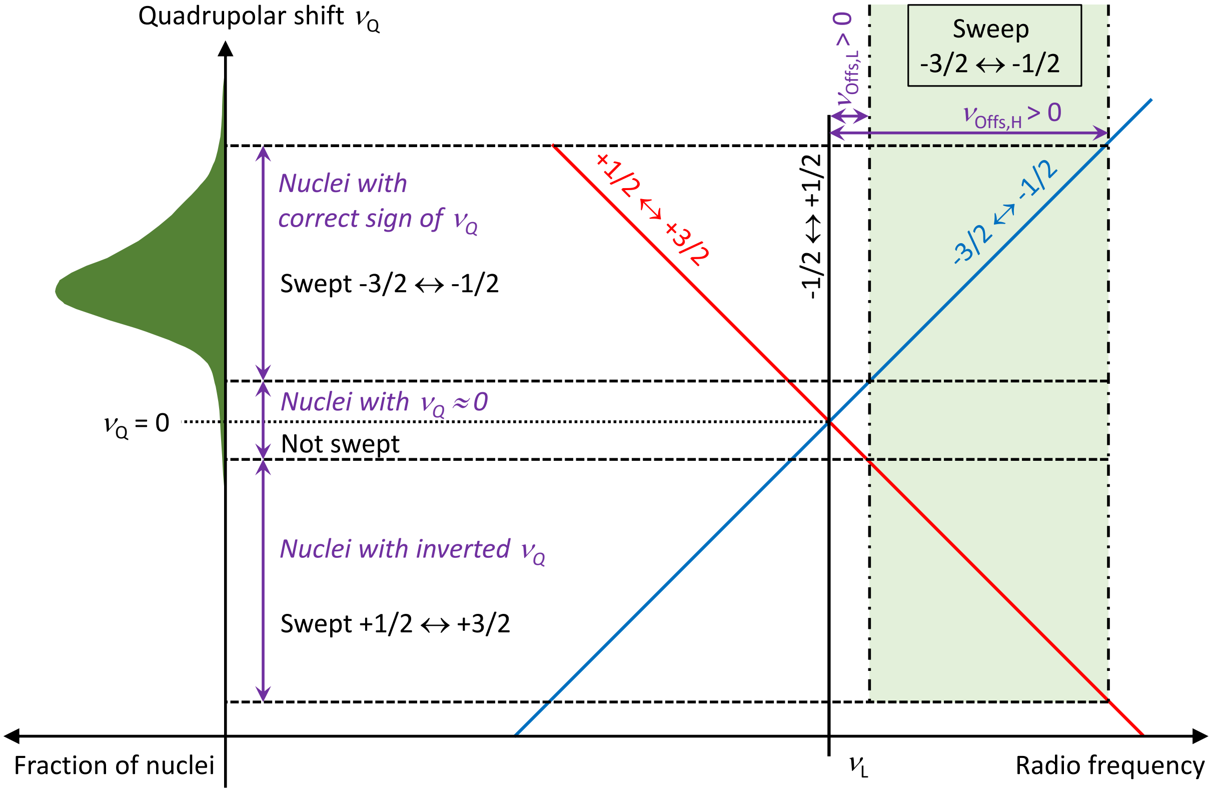

Supplementary Figure 15. **Effect of the nuclei with different quadrupolar shifts in a single-transition adiabatic inversion measurement.**

Fig. 15. Here, rather than saturating the NMR resonance we perform a radiofrequency sweep, which adiabatically inverts the populations of the chosen pair of spin states. Adiabatic sweep has the advantage of doubling the hyperfine shift compared to saturation – this simple relation holds only if a single NMR transition is excited. By contrast, for an adiabatic sweep over multiple quadrupolar NMR transitions the result is more complicated, making comb saturation preferable for two-transition and three-transition NMR excitation. The radiofrequency is always swept in the direction away from the central NMR transition, starting at $\nu_L + \nu_{Offs,L}$ and ending at $\nu_L + \nu_{Offs,H}$. The amplitude and the sweep rate are derived from calibration measurements discussed in Supplementary Note 5 D. For adiabatic sweeping of the $-3/2 \leftrightarrow -1/2$ transition we use $\nu_{Offs,L} = +8$ kHz, $\nu_{Offs,H} = +180$ kHz for ^{75}As and $\nu_{Offs,L} = -5$ kHz, $\nu_{Offs,H} = -50$ kHz for ^{69}Ga. For the sweep over the $+1/2 \leftrightarrow +3/2$ transition the values of $\nu_{Offs,L}$ and $\nu_{Offs,H}$ are inverted. The $-3/2 \leftrightarrow -1/2$ sweep works as designed for the majority of nuclei with $\nu_{Offs,L} < \nu_Q < \nu_{Offs,H}$ (note that both $\nu_{Offs,L}$ and $\nu_{Offs,H}$ are positive in this example). For the small number of nuclei with $-\nu_{Offs,H} < \nu_Q < -\nu_{Offs,L}$ the nominal $-3/2 \leftrightarrow -1/2$ sweep results in an adiabatic inversion of the $+1/2 \leftrightarrow +3/2$ transition instead. The remaining nuclei with $|\nu_Q| < |\nu_{Offs,L}|$ or $|\nu_Q| > |\nu_{Offs,H}|$ are

not affected by the Rf sweep. Thus the observed hyperfine shift $\Delta E_{\mathrm{hf,Obs}}^{-3/2\leftrightarrow-1/2}$ in the $-3/2 \leftrightarrow -1/2$ single-transition sweep experiment can be written as:

$$\Delta E_{\mathrm{hf,Obs}}^{-3/2\leftrightarrow-1/2} = 2\left(c_{\mathrm{Swp,Ideal}}\Delta E_{\mathrm{hf}}^{-3/2\leftrightarrow-1/2} + c_{\mathrm{Swp,Inv}}\Delta E_{\mathrm{hf}}^{+1/2\leftrightarrow+3/2}\right) \quad \text{(S23)}$$

An important property of the single-transition selective excitation is that the nuclei with small absolute quadrupolar shifts ν_{Q} are eliminated from the measured hyperfine shifts. This is preferred over the two-transition saturation measurement, where such nuclei are fully depolarized, resulting in a parasitic hyperfine shift characterised by the $c_{\mathrm{Sat,Full}}$ coefficient in Supplementary Eq. S22.

C. NMR spectra of the QD nuclei

Supplementary Figs. 16a,b show typical nuclear magnetic resonance spectra measured on ^{75}As and ^{69}Ga nuclei in QD1. The three magnetic dipole transitions of each of the spin-3/2 isotopes are split due to the natural elastic strain within the quantum dot volume, arising most likely from the lattice mismatch between the GaAs QD and the AlGaAs barriers. Although the NMR triplet is well resolved, there is a few-percent overlap between the spectral components. When quantifying nuclear spin polarization degrees close to unity, such overlap must be taken into consideration. In order to quantify the spectral overlap, we study a piece of the same QD sample but subject to a uniaxial stress along the [110] crystallographic direction (i.e. the strain is applied perpendicular to the sample growth direction). Nuclear quadrupolar shifts induced by the external stress significantly exceed the intrinsic quadrupolar shifts. As a result the NMR triplet is fully resolved, as can be seen in the inverse NMR spectra of Supplementary Figs. 17a,b where we focus on the $-1/2 \leftrightarrow +1/2$ and $-3/2 \leftrightarrow -1/2$ transitions.

The spectral shapes of the $-3/2 \leftrightarrow -1/2$ satellites measured in a stressed QD sample are similar to those in the unstressed sample (Supplementary Fig. 16). The satellite lineshape consists mainly of an asymmetric peak, but also shows evidence of spectral wings that are broad enough to overlap with the $-1/2 \leftrightarrow +1/2$ central transition in an unstressed sample. In principle, the overlap can be derived by integrating the relevant part of the satellite lineshape, measured with inverse NMR and shown in Supplementary Figs. 17a,b. However, this approach is vulnerable to noise – it is more efficient to incorporate integration into the NMR spectroscopy method [40, 41]. Such an integral NMR measurement is performed by selectively saturating all nuclear spin transitions within a certain spectral band. The high-frequency edge of the saturating band (implemented as a frequency comb) is kept fixed. The low-frequency edge is scanned and the resulting change in

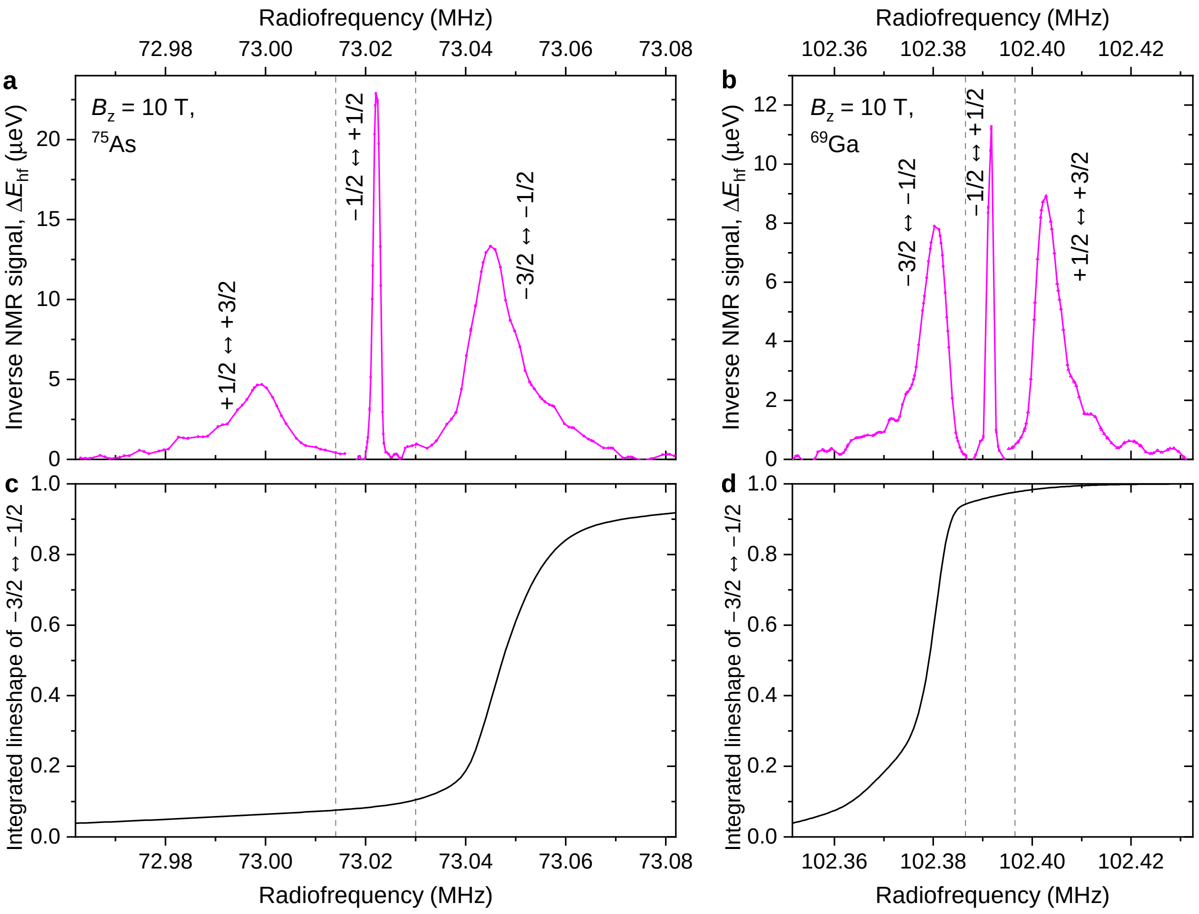

Supplementary Figure 16. **Nuclear magnetic resonance spectra of a single quantum dot. a,** Nuclear magnetic resonance spectra of the ^{75}As nuclei measured in QD1 at B_z = 10 T using the "inverse NMR" technique for signal enhancement [23]. The $-1/2 \leftrightarrow +1/2$ central transition is measured with a 2 kHz resolution, while the satellites $-3/2 \leftrightarrow -1/2$ and $+1/2 \leftrightarrow +3/2$ are measured with a 6 kHz resolution. Vertical dashed lines are offset from the central transition by ±8 kHz and indicate the starting points of the frequency sweeps over the satellite peaks. **b,** Same as (a) but for ^{69}Ga nuclei. The $-1/2 \leftrightarrow +1/2$ central transition is measured with a 2 kHz resolution, while the satellites are measured with a 4 kHz resolution. Vertical dashed lines are offset from the central transition by ±5 kHz. **c,** Integrated lineshape of the $-3/2 \leftrightarrow -1/2$ transition of ^{75}As, derived from experiments on a stressed piece of the same semiconductor QD sample. **d,** Same as (c) but for the $-3/2 \leftrightarrow -1/2$ transition of ^{69}Ga.

the hyperfine shift ΔE_{hf} is measured. This dependence of ΔE_{hf} reveals the fraction of the nuclei covered by the saturating Rf band, and therefore provides a scaled definite integral of the NMR lineshape. For ^{75}As nuclei the fixed-frequency edge of the Rf band is detuned by +500 kHz from the central transition to ensure that the entire $-3/2 \leftrightarrow -1/2$ transition can be covered. For ^{69}Ga nuclei the fixed-frequency edge of the Rf band is detuned by +230 kHz from the central transition,

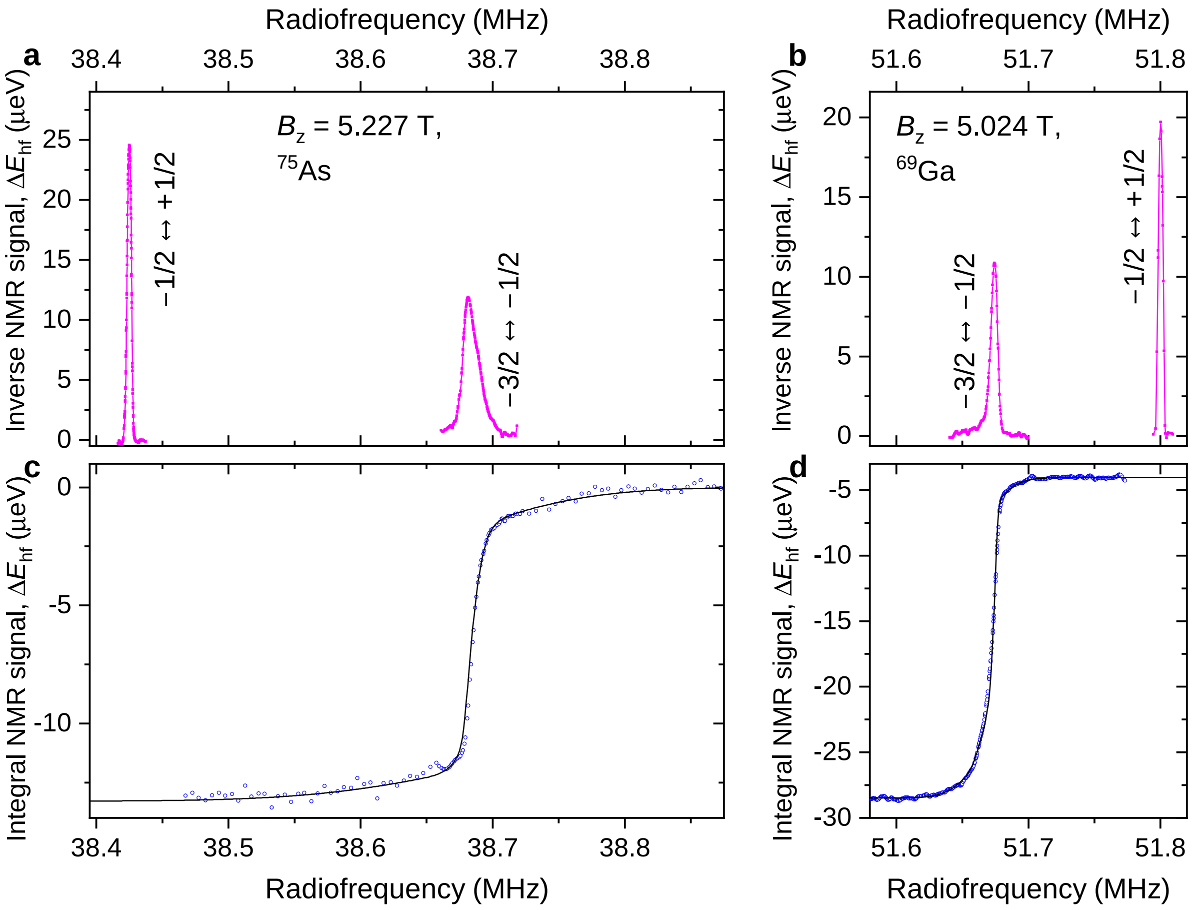

Supplementary Figure 17. **Nuclear magnetic resonance spectra of a quantum dot under external uniaxial stress. a,** Inverse NMR spectrum of ^{75}As at $B_z = 5.227$ T measured with a 4 kHz resolution. **b,** Inverse NMR spectrum of ^{69}Ga at $B_z = 5.024$ T measured with a 4 kHz resolution. **c,** Integral saturation NMR spectrum of ^{75}As measured (symbols) under the same condition as inverse NMR in (a). Line shows a smoothed fitted profile. **d,** Integral saturation NMR spectrum of ^{69}Ga measured under the same condition as inverse NMR in (b).

so that the entire $-1/2 \leftrightarrow +1/2$ and $+1/2 \leftrightarrow +3/2$ transitions are also included in the band, in order to amplify the integral NMR signal of the $-3/2 \leftrightarrow -1/2$ satellite.

Supplementary Fig. 17c shows the integral NMR spectrum of the $-3/2 \leftrightarrow -1/2$ transition of the ^{75}As nuclei in a stressed sample. The steepest rise in the integral signal matches the position of the sharp peak in the inverse NMR spectrum of Supplementary Fig. 17a. However, we also observe the slopes that stretch as far as $\approx \pm 150$ kHz from the satellite peak maximum, indicating the contribution of a broad NMR signal. Broad spectral features are also observed in Supplementary Fig. 17d for ^{69}Ga nuclei, though in a narrower spectral range and with an overall smaller contribution. This broad background can be ascribed to the NMR signal from AlGaAs

barriers or any Al atoms diffusing into the GaAs QD layer [41]. The Al atoms that randomly replace the Ga atoms distort the tetrahedral symmetry of the four nearest neighbours surrounding each As atom. The resulting unit-cell-scale strain results in pronounced quadrupolar shifts. By contrast, all Ga atoms have four identical As atoms as nearest neighbours. Therefore, Ga atoms are affected by Al/Ga random alloying only through next-nearest neighbours, explaining why the broad nuclear quadrupolar wings are smaller than for As nuclei.

Integral NMR spectra are processed in order to derive the correction coefficients. The experimental data is first smoothed (lines in Supplementary Figs. 17c,d) by fitting with a sum of three skew normal distribution peaks. The integral lineshapes are then normalized and shifted along the frequency scale to have the $-3/2 \leftrightarrow -1/2$ satellite NMR peaks in the stressed sample (Supplementary Figs. 17a,b) match the peak positions in the unstressed sample (Supplementary Figs. 16a,b). The resulting integrals of the $-3/2 \leftrightarrow -1/2$ lineshapes are shown by the solid lines in Supplementary Figs. 16c,d and are used to derive the c correction coefficients in Supplementary Eqs. S22, S23. For example, the vertical dashed lines in Supplementary Figs. 16a,b indicate the starting points of the frequency sweeps over the satellite peaks. The integral value at the lower starting point for ^{75}As is ≈ 0.07 and approximately corresponds to the fraction $c_{\mathrm{Swp,Inv}}$ of the nuclei where the $+1/2 \leftrightarrow +3/2$ satellite is swept instead of the intended $-3/2 \leftrightarrow -1/2$. The difference of the integral at the higher and lower sweep starting points gives approximately the fraction of nuclei (≈ 0.03) that are not swept at all. The summary of all the coefficients derived from the integrated lineshapes can be found in the following table:

Coefficient	^{75}As	^{69}Ga
$c_{\mathrm{Sat,Ideal}}$	0.8979	0.9424
$c_{\mathrm{Sat,Full}}$	0.0170	0.0341
$c_{\mathrm{Sat,Inv}}$	0.0750	0.0234
$c_{\mathrm{Sat,CT}}$	0.0100	0.0003
$c_{\mathrm{Swp,Ideal}}$	0.8893	0.9272
$c_{\mathrm{Swp,Inv}}$	0.0721	0.0234

(S24)

It can be seen that the contributions of the ideal signals are higher for the ^{69}Ga nuclei due to their smaller inhomogeneous quadrupolar broadening. As a result, nuclear spin polarization measurements are more accurate for ^{69}Ga than for ^{75}As. It is worth noting that the nuclear spin thermometry data measured on QDs in an unstrained sample is corrected with the c coefficients measured on a different individual QD (in a stressed sample). However, measurements conducted

on several individual QDs from the same sample reveal NMR spectra very similar to those shown Supplementary Fig. 16a,b. Thus, while there is always some uncertainty arising from dot-to-dot variation, its effect is expected to be smaller than the actual correction introduced through the c coefficients.

D. Calibration of the adiabatic radiofrequency sweeps

Supplementary Fig. 18 shows the dependence of the Rf-induced hyperfine shift on the frequency sweep rate. The amplitude of the Rf field is expressed in terms of the corresponding Rabi frequency ν_1. Supplementary Fig. 18a shows the results of an experiment where radiofrequency is swept from -50 kHz to $+50$ kHz around the ^{69}Ga Larmor frequency. This sweep range covers nearly the entire ^{69}Ga quadrupolar triplet. For a sufficiently large Rf amplitude $\nu_1 \gtrsim 1.64$ kHz and a sufficiently low rate the sweep is adiabatic, resulting in population transfer from the optically-populated $m = -3/2$ states into the $m = +3/2$ states. The variation of the hyperfine shift under adiabatic conditions is $\Delta E_{\rm hf} \approx 53$ μeV (dashed horizontal line). As expected [42], the sweep rate that produces adiabatic transfer increases quadratically with the Rf amplitude ν_1. When ν_1 is reduced below ≈ 1 kHz, the magnitude of the hyperfine shift $\Delta E_{\rm hf}$ in the slow-sweep limit decreases, indicating that population transfer becomes non-adiabatic. This non-adiabaticity is a result of demagnetization in the rotating frame, where Zeeman energy is transferred into the nuclear dipole-dipole interaction reservoir [10, 42, 43]. For all ν_1 the sweep also becomes non-adiabatic in the large-rate limit.

Supplementary Fig. 18b shows sweep rate dependence for the range starting from -5 kHz to -50 kHz, which selectively covers the $-3/2 \leftrightarrow -1/2$ satellite NMR transition (the starting points of the sweeps are shown by the dashed lines in Supplementary Fig. 16b). The adiabatic inversion of the subspace spanned by the $m = -1/2$ and $m = -3/2$ states results in a hyperfine shift of $\Delta E_{\rm hf} \approx 20$ μeV. Unlike for $-1/2 \leftrightarrow +1/2$, adiabaticity is achieved at a lower Rf amplitude $\nu_1 \gtrsim 0.27$ kHz. This is explained by the difference in the inhomogeneous broadening of the satellite transitions and the central transition $-1/2 \leftrightarrow +1/2$. In case of the $-1/2 \leftrightarrow +1/2$ transition (that is driven when the frequency is swept over the entire quadrupolar triplet) the inhomogeneous broadening is due to the second order quadrupolar shifts which are small compared to dipolar nuclear-nuclear interactions. By contrast, the first-order inhomogeneous quadrupolar broadening of the $-3/2 \leftrightarrow -1/2$ satellite ($\gtrsim 10$ kHz) is much larger than the dipole-dipole interaction. As a result the Zeeman and the dipolar energy reservoirs remain isolated during the sweep over the satellite, inhibiting the demagnetization. In other words, the Rf field sweeping over the broadened

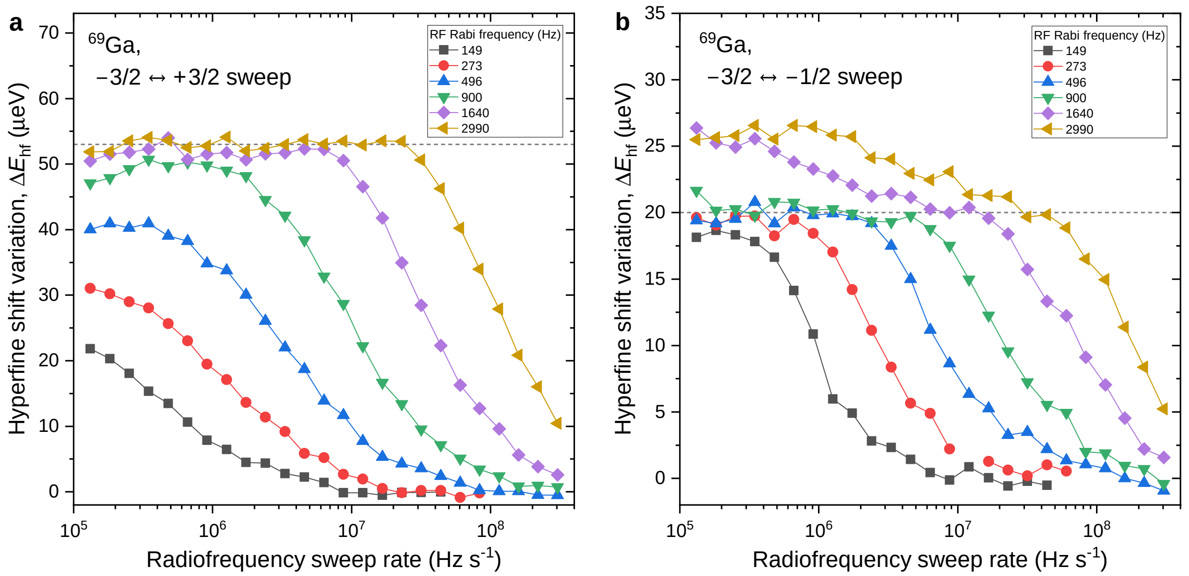

Supplementary Figure 18. **Calibration of the radiofrequency adiabatic sweeps. a,** In these experiments σ^+ optical pumping is first used to produce negative nuclear spin polarization. Optical pumping is followed by a frequency-swept Rf burst and the resulting hyperfine shift variation is plotted as a function of the frequency sweep rate. The radiofrequency field is swept from −50 kHz to +50 kHz with respect to the Larmor frequency of the ^{69}Ga nuclear spins. This range covers all three quadrupolar-split NMR transitions. Results are shown for several amplitudes of the radiofrequency field, expressed in terms of the Rabi frequency ν_1 that such a field produces when tuned in resonance with the satellite NMR transition $-3/2 \leftrightarrow -1/2$. Dashed horizontal line shows the hyperfine shift variation under adiabatic conditions. **b,** Same as (a), but the frequency range of the sweep is from −5 kHz to −50 kHz, covering only the satellite NMR transition $-3/2 \leftrightarrow -1/2$.

$-3/2 \leftrightarrow -1/2$ satellite excites only a small fraction of the nuclei at any given frequency, while the majority of the nuclear spins remain out of resonance and therefore cannot participate in the exchange between the Zeeman and dipolar reservoirs. When the Rf amplitude is increased ($\nu_1 \gtrsim 1.64$ kHz), the magnitude $|\Delta E_{hf}|$ of the hyperfine shift increases further beyond the adiabatic-inversion level. This can be explained by the parasitic driving of the $-1/2 \leftrightarrow +1/2$ transition, which occurs when ν_1 becomes non-negligible compared to the minimal offset (−5 kHz from the $-1/2 \leftrightarrow +1/2$ frequency) during the sweep over the $-3/2 \leftrightarrow -1/2$ transition. Based on these calibrations, we use adiabatic frequency sweeps only on the $\pm 3/2 \leftrightarrow \pm 1/2$ satellites, avoiding any sweeps that involve the $-1/2 \leftrightarrow +1/2$ central transition. For the spin temperature measurements on ^{69}Ga we use $\nu_1 \approx 0.496$ kHz and a sweep rate of 1 MHz s^{-1}. From the data of Supplementary Fig. 18b this combination of parameters is seen to provide good adiabatic inversion of the satellite

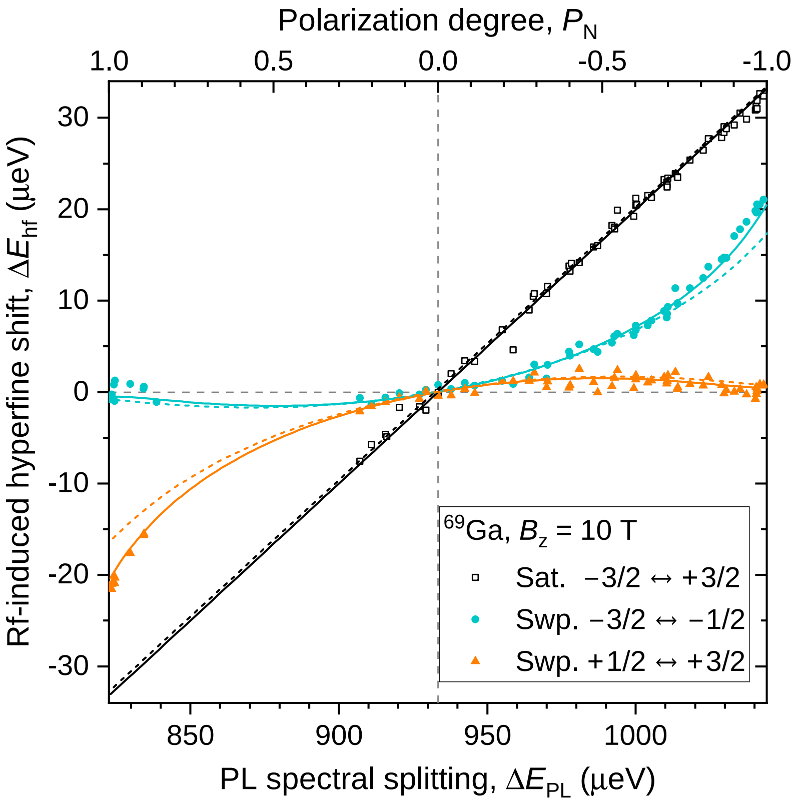

Supplementary Figure 19. **Derivation of nuclear spin polarization from selective NMR excitation.** Spectral splitting $\Delta E_{\rm PL}$ of a QD negatively charge trion measured without Rf excitation is plotted on the bottom horizontal axis. Rf-induced hyperfine shift variation $\Delta E_{\rm hf}$ is plotted on the vertical axis. Each point is obtained by changing the initial degree of the optically induced nuclear spin polarization and conducting two measurements: with selective Rf excitation and without Rf excitation. $\Delta E_{\rm hf}$ is the difference of these two measurements. Several types of ^{69}Ga Rf excitation are employed: non-selective saturation of the entire NMR triplet (squares), adiabatic frequency sweep over the $-3/2 \leftrightarrow -1/2$ satellite (circles) and adiabatic frequency sweep over the $+1/2 \leftrightarrow +3/2$ satellite (triangles). Solid lines show the best fit, whereas dashed lines show a fit constrained by the $|P_{\rm N}| < 0.9$ hypothesis. The top horizontal scale shows the nuclear spin polarization degree $P_{\rm N}$ evaluated from the best fit. Experiments are conducted on an individual QD1 at $B_{\rm z} = 10$ T.

transitions without any noticeable parasitic excitation of the central transition. Similar results were obtained from calibrations on the ^{75}As satellite transition resonance – the optimal Rf amplitude was found to be $\nu_1 \approx 0.560$ kHz, with a sweep rate of 0.8 MHz s^{-1}.

E. Model fitting for derivation of the nuclear spin polarization degree

The experimental measurement of the nuclear spin polarization (spin thermometry) uses the Pump-Rf-Probe cycle shown in Supplementary Fig. 3a. The variable parameter is the initial degree of nuclear spin polarization produced by the optical pumping. The steady-state nuclear spin polarization is changed either by detuning the pump laser wavelength away from the optimum or by altering the degree of circular polarization of the pump. For any given initial nuclear polarization the Pump-Rf-Probe measurements are carried out with different types of NMR Rf excitation or with no Rf pulse at all. The spectral splitting of the trion ΔE_{PL} detected in the probe pulse is then used as the horizontal axis for the data plots in Supplementary Fig. 19. On the vertical axis we plot the difference between the trion spectral splitting measured with the Rf pulse (final state) and without the Rf pulse (initial state). This difference yields the change in the hyperfine shift resulting purely from the selective Rf excitation of a certain NMR transition for a chosen isotope, whereas hyperfine shifts arising from other transitions and isotopes remain unaffected. In the experiment we avoid a certain range of positive initial nuclear spin polarizations (characterised by 850 μeV $< \Delta E_{\mathrm{PL}} <$ 900 μeV for QD1 at $B_z = 10$ T) where electron spin energy splitting is close to zero due to the hyperfine shift and the Zeeman effect cancelling each other out. Such cancellation is characterised by accelerated nuclear spin dynamics (observed as a kink in Fig. 4a of the main text), making it difficult to perform non-perturbing optical probing. Therefore, in order to discuss the spin thermometry fitting, we focus on the negative nuclear polarizations, where most of the datapoints are collected (Supplementary Fig. 20).

The splitting in the photoluminescence spectrum of a negatively charged trion X^- (see Supplementary Fig. 2b) can be written as (see Supplementary Eq. S2):

$$\Delta E_{\mathrm{PL}} = \Delta E_{\mathrm{PL},0} - \sum_j F^{(j)} I_j P_{\mathrm{N},j}. \tag{S25}$$

where $\Delta E_{\mathrm{PL},0}$ is the trion splitting corresponding to depolarized nuclei and the summation goes over all isotope species with their individual polarization degrees $P_{\mathrm{N},j}$. The individual proportionality constants can be written as $F^{(j)} = k_j(A^{(j)} - C^{(j)})$, where both the electron ($A^{(j)}$) and the hole ($C^{(j)}$) hyperfine material constants are included since the photoluminescence of the trion is only observed for recombination of an electron and a hole with the opposite spin z projections (see Supplementary Note 2). The factors $0 < k_j \leq 1$ account for the Ga nuclei atoms replaced by Al, resulting in a reduced hyperfine shift experienced by the electron spin. If all $I = 3/2$ isotopes have

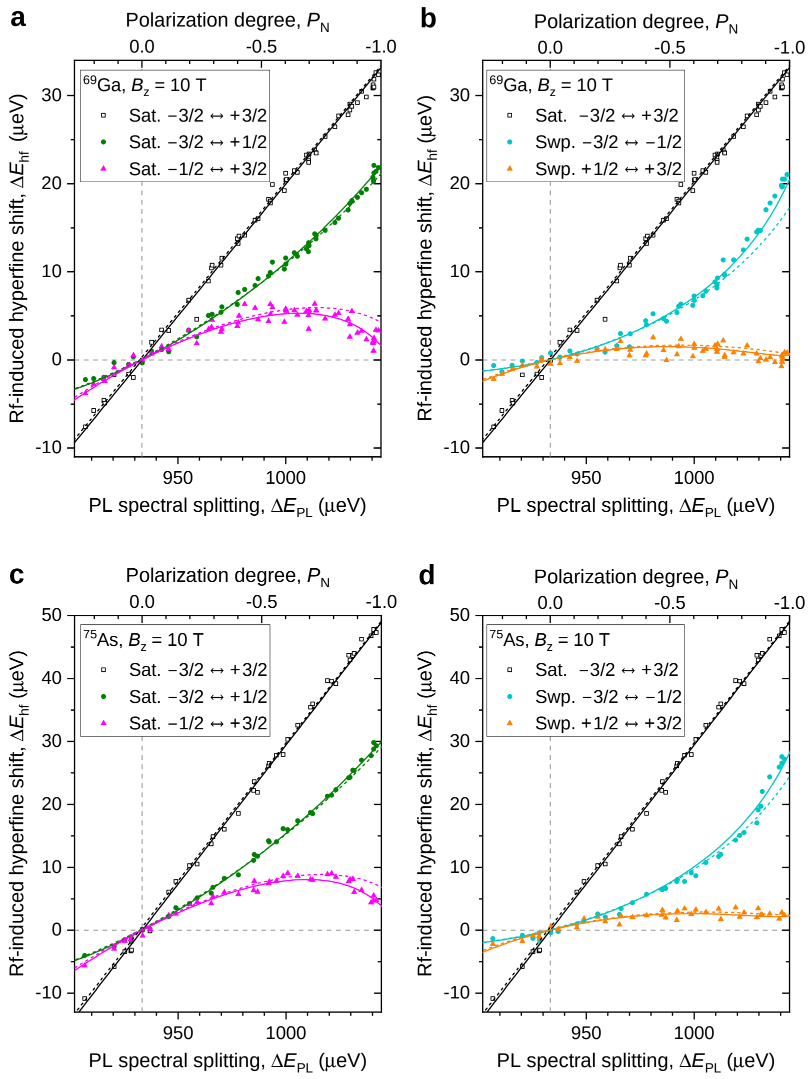

Supplementary Figure 20. **Derivation of nuclear spin polarization from selective NMR measurements.** Same as Supplementary Fig. 19, but focusing on the range of negative nuclear spin polarizations. **a,** Data and fits for ^{69}Ga nuclei. Hyperfine shift variations are shown for saturation of the entire NMR triplet (squares) and selective two-transition saturation (circles for $-3/2 \leftrightarrow +1/2$, triangles for $-1/2 \leftrightarrow +3/2$). **b,** Data for the non-selective saturation (same data as in (a), squares) and selective adiabatic frequency sweeps over NMR satellites (circles for $-3/2 \leftrightarrow -1/2$, triangles for $+1/2 \leftrightarrow +3/2$). **c, d,** Same as (a,b) but for ^{75}As nuclei.

the same polarization degree, Supplementary Eq. S25 simplifies to

$$\Delta E_{\mathrm{PL}} = \Delta E_{\mathrm{PL},0} - I P_{\mathrm{N}} \sum_j F^{(j)}. \tag{S26}$$

Resolving this for P_{N} and substituting into the last of Supplementary Eq. S21, we find that the change in the hyperfine shift (final minus initial) arising from the non-selective saturation of all

three NMR transitions of the j-th isotope is a linear function of the trion spectral splitting:

$$\Delta E_{\mathrm{hf},j}^{-I\leftrightarrow+I} = (\Delta E_{\mathrm{PL}} - \Delta E_{\mathrm{PL},0})\frac{F^{(j)}}{F_{\mathrm{tot}}} = w_j(\Delta E_{\mathrm{PL}} - \Delta E_{\mathrm{PL},0}),$$
$$F_{\mathrm{tot}} = \sum_j F^{(j)}, \tag{S27}$$

where w_j is the weight coefficient of the j-th isotope in the total hyperfine shift E_{hf}, and F_{tot} is the total proportionality factor. The measured $\Delta E_{\mathrm{hf},j}^{-I\leftrightarrow+I}$ are shown by the squares in Supplementary Figs. 20a,c for ^{75}As and ^{69}Ga, respectively. The dependence on ΔE_{PL} is indeed seen to be linear. For precise modelling we take the squared differences between the measured $\Delta E_{\mathrm{hf},j}^{-I\leftrightarrow+I}$ and $\Delta E_{\mathrm{hf},j}^{-I\leftrightarrow+I}$ computed from Supplementary Eq. S27 with spectral splitting ΔE_{PL} measured under the same optical pumping but without radiofrequency depolarization.

The same approach is applied to the hyperfine shift variations $\Delta E_{\mathrm{hf},j}^{m\leftrightarrow m+1}$ and $\Delta E_{\mathrm{hf},j}^{m\leftrightarrow m+2}$ arising from selective saturation (or adiabatic inversion) of one or two NMR transitions, respectively. Here, Supplementary Eq. S26 is first resolved to find P_{N} as a function of ΔE_{PL}, and P_{N} is then substituted into Supplementary Eq. S17 to find β. Since there is no explicit form for the inverse of the Brillouin function, this relation is kept in an exact analytical form using the Root[ ] function in Wolfram Mathematica 12.3 software. The inverse temperature β is then inserted into Supplementary Eqs. S21. Finally, the ideal $\Delta E_{\mathrm{hf},j}^{m\leftrightarrow m+1}$ and $\Delta E_{\mathrm{hf},j}^{m\leftrightarrow m+2}$ calculated in this way, are inserted into Supplementary Eqs. S22, S23 using the c coefficients from Supplementary Eq. S24 to account for the small spectral overlaps between the individual components of the quadrupolar NMR triplet. Taking these model hyperfine shifts at the experimentally measured ΔE_{PL}, we calculate the squared differences with respect to the measured Rf-induced hyperfine shifts (triangles and circles in Supplementary Figs. 20a,c for two-transition saturation and in Supplementary Figs. 20b,d for single-transition sweeps). We then sum up the squared differences for all individual Rf types and all isotopes to form the total χ^2 functional.

As a last step, we include in our model the possibility that different isotopes have different polarization degrees $P_{\mathrm{N},j}$. For arbitrary $P_{\mathrm{N},j}$ it is not possible to resolve the trion PL splitting ΔE_{PL} as a function of $P_{\mathrm{N},j}$, requiring some explicit assumptions. As a simplest approximation, we assume that polarization degrees of the three abundant spin-3/2 isotopes (^{75}As, ^{69}Ga and ^{71}Ga) are linearly interdependent. Mathematically, this is equivalent to allowing the total scaling factor F_{tot} to deviate for the different measured isotopes. The introduction of F_{tot} and w_j as model fitting parameters is also convenient in that the data does not have to be collected on all isotopes, in particular on ^{71}Ga, which has not be studied in this work.

The χ^2 is a function of only five fitting parameters: the zero-polarization trion splitting $\Delta E_{\mathrm{PL},0}$,

the total scaling factors $F_{\rm tot}$ of ^{75}As and ^{69}Ga as well as the weight coefficients w_j of ^{75}As and ^{69}Ga. In case of QD1, where the data was measured at two different magnetic fields, we fit these datasets independently in order to account for the different degree to which the probe laser pulse introduces parasitic depolarization in the optically measured hyperfine shifts. The best fits obtained by minimizing the χ^2 functional are plotted by the solid lines in Supplementary Fig. 20 and show a good match to the measured data. The best fit parameters are listed below together with the total number of experimental datapoints $N_{\rm data}$ and the root-mean-square (RMS) residual $R_{\rm min} = \sqrt{\chi^2_{\rm min}/N_{\rm data}}$ derived from the minimized functional value $\chi^2_{\rm min}$. In addition, we quote the residual $R'_{\rm min}$ obtained from a separate linear fit where only the three-transition saturation hyperfine shift $\Delta E_{{\rm hf},j}^{-I\leftrightarrow+I}$ is considered:

QD	$B_{\rm z}$	$w(^{75}\rm As)$	$w(^{69}\rm Ga)$	$IF_{\rm tot}(^{75}\rm As)$	$IF_{\rm tot}(^{69}\rm Ga)$	$N_{\rm data}$	$R_{\rm min}$	$R'_{\rm min}$
QD1	4 T	0.416	0.292	112.0 μeV	104.5 μeV	346	0.875 μeV	0.803 μeV
QD1	10 T	0.442	0.300	112.3 μeV	110.7 μeV	510	0.749 μeV	0.778 μeV
QD2	10 T	0.429	0.294	112.3 μeV	110.3 μeV	336	1.108 μeV	0.946 μeV
QD3	10 T	0.424	0.294	113.9 μeV	112.8 μeV	170	0.987 μeV	0.968 μeV

(S28)

As discussed above, the three-transition saturation experiment is the most robust against the errors arising from NMR spectral overlaps. Therefore, the RMS residual $R'_{\rm min}$ obtained from linear fitting of $\Delta E_{{\rm hf},j}^{-I\leftrightarrow+I}$ alone characterizes the true random measurement errors. These errors in the optically-detected hyperfine shifts originate mainly from the noise of the CCD detector used to collect the optical photoluminescence spectra of a single quantum dot. Any excess of $R_{\rm min}$ obtained from the nonlinear fit of the entire selective-NMR dataset over $R'_{\rm min}$ is an indicator of systematic deviation between the model and the data. According to the table of Supplementary Eq. S28 such excess is small, confirming the validity of the Boltzmann distribution model (Supplementary Eq. S14). The spread in the isotope-specific weights w_i, and the scaling factor $F_{\rm tot}$ is on the order of 1% for the $B_{\rm z} = 10$ T data collected from three individual quantum dots, affirming the systematic nature of these results. The fit of the $B_{\rm z} = 4$ T data shows the most deviation, which is explained by the need for shorter probe pulses $T_{\rm Probe}$, resulting in more noisy photoluminescence spectra as well as larger systematic deviations arising from the probe-induced nuclear spin depolarization.

In order to derive the nuclear spin polarization degree, we use the highest and the lowest trion spectral splitting $\Delta E_{\rm PL}$ detected without any radiofrequency manipulation in a separate measurement. For this measurement we use the timing diagram of Supplementary Fig. 3d, where we allow the nuclear spin polarization to build up over $T_{\rm Buildup} > 100$ s, giving a closer approach to the

steady state than what can be achieved in the NMR thermometry measurements (Supplementary Fig. 20), where the pumping time $T_{\rm Pump} \lesssim 30$ s is limited by the maximum duration of the CCD detector exposure. It is worth noting that this approach of using the separately measured steady-state $\Delta E_{\rm PL}$ is the reason why we build our fitting model on relating the polarization degree $P_{\rm N}$ to spectral splitting $\Delta E_{\rm PL}$ via Supplementary Eq. S25. Otherwise, $\Delta E_{\rm PL}$ can be eliminated and polarization degree can be derived purely from the Rf-induced hyperfine shifts of Supplementary Eq. S21. The best fit value of $\Delta E_{\rm PL,0}$ is subtracted from the steady state $\Delta E_{\rm PL}$ to derive the lowest negative and the highest positive hyperfine shifts. For QD1 at $B_{\rm z} = 10$ T we find $E_{\rm hf} = -109.6$ μeV and $E_{\rm hf} = +112.3$ μeV. The latter number exceeds the best-fit product $IF_{\rm tot}(^{69}{\rm Ga})$ by $\approx 1\%$. By definition, the $IF_{\rm tot}$ product is the maximum $|E_{\rm hf}|$ corresponding to full polarization $P_{\rm N} = \pm 1$. The discrepancy with the measured $E_{\rm hf}$ reveals the scale of errors in the derived polarization degrees $P_{\rm N}$, both due to the random noise in the raw data and any systematic inaccuracy of the fitting model.

F. Error analysis in model fitting of the nuclear spin polarization data

In order to systematically analyze the fitting errors we construct a multidimensional confidence region (Chapter 9 in Ref.[44]) defined as a collection of all points in the fitting parameter space for which

$$\chi^2 < (1 + Q(\gamma, n)/N_{\rm data})\chi^2_{\rm min}, \tag{S29}$$

where we have approximated the standard error in the experimental data by the RMS fit residual $R_{\rm min} = \sqrt{\chi^2_{\rm min}/N_{\rm data}}$. (Note that here we define χ^2 and $\chi^2_{\rm min}$ as sums that are not normalized by the standard error.) We define $Q(\gamma, n)$ as a quantile of the χ^2-distribution with n parameters corresponding to the confidence level $1 - \gamma$. We use $1 - \gamma = 0.95$ where the relevant quantile is $Q(1 - 0.95, 5) \approx 11.07$. We implement a Monte-Carlo calculation, where the χ^2 sum is computed for a large number of random sets of the fitting parameters around the best-fit point. For each trial parameter set that satisfies Supplementary Eq. S29 we calculate the polarization degrees $P_{\rm N}$ from the maximum and minimum steady-state spectral splitting $\Delta E_{\rm PL}$ measured with long $T_{\rm Buildup} > 100$ s. Finally, the confidence intervals are derived separately for the maximum positive and the minimum negative polarization degree as maximum and minimum $P_{\rm N}$ values from the random Monte-Carlo set, coerced to satisfy the condition $-1 \leq P_{\rm N} \leq 1$. The confidence intervals

are shown by the symbols in Fig. 3e of the main text and are tabulated below:

QD	B_z	max ^{75}As P_N	min ^{75}As P_N	max ^{69}Ga P_N	min ^{69}Ga P_N
QD1	4 T	$[0.890, 0.974]$	$[-0.989, -0.903]$	$[0.982, 1]$	$[-1, -0.998]$
QD1	10 T	$[0.969, 1]$	$[-0.994, -0.954]$	$[1, 1]$	$[-1, -0.984]$
QD2	10 T	$[0.956, 1]$	$[-1, -0.962]$	$[0.989, 1]$	$[-1, -0.998]$
QD3	10 T	$[0.954, 1]$	$[-1, -0.951]$	$[0.964, 1]$	$[-1, -0.959]$

(S30)

This systematic evaluation agrees with the rough estimates above, confirming that the accuracy of our P_N estimates is on the other of a few percent. The fit returns similar values for polarization degrees of ^{75}As and ^{69}Ga – this is expected for a spin pumping mechanism [13] where the inverse temperature β of each individual nucleus is independently equilibrated with the β of a spin-polarized electron. The somewhat wider confidence intervals of ^{75}As could be simply due to the larger overlaps of the NMR spectral components, making the fit less sensitive to P_N and more dependent on the accuracy of the c coefficients tabulated in Supplementary Eq. S24. The derived $|P_N|$ values are similarly high at $B_z = 4$ and 10 T, in a sense that their deviation from unity is comparable to the uncertainty of the estimate. For that reason, it is currently not possible to make any conclusion about the dependence of nuclear polarization on the external magnetic field, which is a point of interest for a follow up work.

In order to further evaluate the error estimates we approach the problem of data modelling from the opposite direction. Namely, we start with a hypothesis that the maximum absolute polarization degree $|P_N|$ is no more than a certain value < 1, and then evaluate how well our experimental data can be matched to this hypothesis. In order to demonstrate this approach, we constrain the fit to $|P_N| < 0.9$ and search for the fitting parameter combination that minimizes the χ^2 functional (using the same model as the one used to derive the unconstrained best fit). The resulting constrained best-fit is shown for QD1 by the dashed lines in Supplementary Fig. 20. There are visible systematic deviations from the measured data, already suggesting that the $|P_N| < 0.9$ hypothesis is inadequate, and the actual absolute polarization degree is well above 0.9. Quantitatively, the RMS residual from the fit of the $B_z = 10$ T datasets constrained to $|P_N| < 0.9$ is ≈ 1.73 μeV for QD1, ≈ 2.91 μeV for QD2 and ≈ 2.17 μeV for QD3. These residuals are a factor of $\gtrsim 2$ larger than the best-fit residuals tabulated in Supplementary Eq. S28 – statistically, such deviations are improbable for our datasets containing hundreds of datapoints. It then follows that the $|P_N| < 0.9$ hypothesis should be rejected, leading to a conclusion that polarization is in fact well above 0.9.

Next, we fit the same experimental data but without correcting for the overlaps of the NMR spectral components. This is equivalent to setting $c_{\mathrm{Sat,Ideal}} = c_{\mathrm{Swp,Ideal}} = 1$ with the remaining

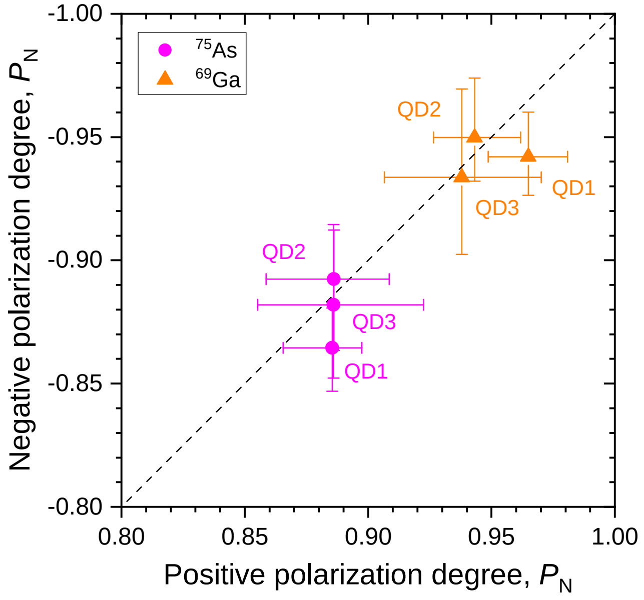

Supplementary Figure 21. **Nuclear spin polarization derived from an uncorrected fit.** Maximum positive (horizontal axis) and minimum negative (vertical axis) nuclear spin polarization degree $P_{\rm N}$ derived from the measurements on ^{75}As (circles) and ^{69}Ga (triangles) nuclei in individual dots QD1 - QD3. The $P_{\rm N}$ values in this plot are derived from a fit that ignores the small spectral overlaps between the NMR triplet components. Error bars are 95% confidence intervals.

c coefficients set to 0. Such a fit can be seen as a lower bound estimate for the absolute polarization degree $|P_{\rm N}|$. Without correction, the RMS fit residual slightly increases from 0.749 μeV to 0.819 μeV for QD1 at $B_{\rm z} = 10$ T. The resulting uncorrected $P_{\rm N}$ are shown in Supplementary Fig. 21. The uncorrected polarization degrees for ^{75}As ($P_{\rm N} \approx 0.88$) are lower than for ^{69}Ga ($P_{\rm N} \approx 0.94$), contradicting the expectation of equal β across different isotopes. Moreover, the maximum uncorrected $|P_{\rm N}|$ are very close to the corresponding $c_{\rm Sat,Ideal}$ coefficients. Additional computations confirm that this is to be expected – a naive uncorrected fit of the data affected by NMR spectral overlap returns for fully polarized nuclei a reduced polarization $|P_{\rm N}|$ which roughly equals the fraction of the "ideal" nuclei that are not affected by the overlap. Nevertheless, even without the corrections, a high polarization degree is derived for ^{69}Ga nuclei, since they are less prone to NMR spectral overlaps than ^{75}As.

Finally, we discuss the different possible sources of systematic errors. Since electron localization in a GaAs quantum dot is not infinitely strong, the electron wavefunction leaks into the AlGaAs barriers where it gradually decays with the increasing distance from the dot. This means that both the nuclear spin pumping efficiency and the sensitivity of the electron hyperfine shift to nuclear

spin polarization are spatially inhomogeneous. The resulting steady-state nuclear spin polarization is also spatially inhomogeneous, if only because the QD layer is sandwiched between the two doped semiconductor layers, where free charge carriers result in $P_{\rm N} \approx 0$. On the other hand, when considering spins as a quantum resource, a significant role is played only by the nuclei within the QD electron wavefunction. Recent studies of nuclear spin relaxation in the same sample have shown that spin diffusion is the dominant mechanism of nuclear spin decay in a QD [15]. The nuclear spins at the center of the QD are quickly polarized by the optically-pumped electron spin and then transfer their polarization to more distant nuclei via nuclear spin flip-flops. For a sufficiently long pumping the nuclei in the AlGaAs barriers around the dot become gradually polarized. This manifests in a slow-down of the subsequent relaxation without the pump. The relaxation times of the nuclear spins are on the order of hundreds of seconds, much longer than the nuclear spin buildup times, which are less than one second. Such a large ratio of the timescales suggests that competition between spin pumping and polarization leakage would not be a limiting factor for achieving $|P_{\rm N}|$ up to ≈ 0.99. Moreover, relaxation much slower than pumping means that spin diffusion creates a smooth spatial profile of the nuclear spin polarization – the extent of the polarized volume is larger than the volume of the electron wavefunction. Therefore, we expect that the electron probes a volume with a nearly uniform nuclear spin polarization degree $P_{\rm N}$. In other words, the existence of spin diffusion combined with long nuclear spin pumping means that there is no realistic mechanism that would result in abrupt spatial variations of $P_{\rm N}$. Under these conditions the selective-NMR thermometry measurements return the average of nuclear polarization, weighted by the electron envelope wavefunction density. Then, observation of a near-unity average polarization itself implies that polarization is very homogeneous for all nuclei within the electron wavefunction volume. For example, if we take the typical leakage of the electron wavefunction into the AlGaAs barriers at ≈ 0.1 (estimated previously in Ref. [13]) and assume full polarization within the GaAs layer ($|P_{\rm N}| = 1$), then observation of a weighted average of $|P_{\rm N}| = 0.99$ implies that polarization within the AlGaAs barriers cannot be much smaller than $|P_{\rm N}| = 0.9$. To summarize, although our present technique is not capable of revealing the spatial profile of the nuclear spin polarization, the most plausible hypothesis is that nuclear spin polarization achieved under steady-state optical pumping is nearly uniform within the volume of the QD electron wavefunction. In practice, this implies the ability to polarize nearly all nuclei whose coupling to the electron is strong enough to have any relevance to electron-nuclear coherent spin dynamics [14]. This also justifies our model, which assumes $P_{\rm N}$ to be constant within the quantum dot volume and its surrounding. From the ratio of the nuclear spin buildup and decay times we estimate the errors caused by $P_{\rm N}$ inhomogeneity to

be on the order of 1%.

Although ^{75}As, ^{69}Ga and ^{71}Ga are the three abundant isotopes, the inevitable penetration of the electron into the AlGaAs barriers implies some hyperfine interaction with the spin-5/2 ^{27}Al nuclei. And yet it turns out that ^{27}Al hyperfine shift is too small to be studied quantitatively with our present spin thermometry techniques. As it has been shown previously [13], this is a combined effect of several factors. The small fraction of the wavefunction overlapping with AlGaAs (≈ 0.1), the small fraction of Al atoms (0.33 in our sample) and the small hyperfine constant (≈ 0.3 of that of As and Ga) mean that the ^{27}Al relative contribution to the total electron hyperfine shift is within $\approx 1\%$. In addition to that, the lack of Al at the center of the QD, where the overlap with the electron is the strongest, suggests inhibition of the pumping-through-diffusion mechanism discussed above. The ^{27}Al spins can only be polarized through direct (and weak) contact with the spin-polarized electron, meaning that aluminium polarization can be reduced. In the context of the present work where we focus on polarization of As and Ga, these observations mean that any systematic errors arising from ^{27}Al are small (within $\approx 1\%$). Investigation of ^{27}Al spin polarization would be an interesting subject for future work – this would require more sensitive experimental techniques, such as trigger detection via abundant isotopes [36].

Summarising this analysis, we see that there is a handful of potential error sources, both random and systematic, but all on the order of 1%. Taking a conservative approach we conclude with confidence that nuclear spin polarization degrees well above 0.95 are achieved. In reality, the polarization is likely to be higher, with rigorous confidence-interval analysis returning polarization degrees as high as $|P_{\rm N}| \approx 0.99$ (for ^{69}Ga at high magnetic field $B_{\rm z} = 10$ T in all three selected individual quantum dots). Achieving even higher polarizations would depend critically on development of more sensitive thermometry techniques. One possibility is to use the dephasing dynamics of the electron spin qubit, since this would gain sensitivity at high polarizations as $\propto 1/\sqrt{1-P_{\rm N}^2}$ (Ref. [39]).

Supplementary Note 6. RAW DATA

Raw data is provided in text files using a tab-separated format.

Photoluminescence (PL) spectra (Figs. 2a,b of the main text) are tabulated in files `Fig2a.tsv` and `Fig2b.tsv`, respectively. Each file is a matrix of the measured PL intensity values. There are 787 rows, each row corresponding to a fixed sample gate bias $V_{\rm Gate}$. The bias changes linearly from $V_{\rm Gate} = +1.43$ V for the first row, to $V_{\rm Gate} = -2.5$ V for the last row. There are 1617

columns, each column corresponding to a fixed PL energy E_{PL}. The energy changes linearly from $E_{\mathrm{PL}} = 1.4995$ eV for the first column, to $E_{\mathrm{PL}} = 1.83961$ eV for the last column.

The file `EhfPumpDep(sigma+).txt` contains the data for the dependence of the hyperfine shift E_{hf} on the optical pumping parameters. The data is for σ^+ optical pumping. Each line represents a measurement datapoint. The first column is the sample bias V_{Pump} during the optical pumping in V. The second column is the measured optical pump power P_{Pump} in μW. The third column is the measured optical pump photon energy E_{Pump} in eV. The fourth column is the measured QD hyperfine shift E_{hf} in μeV. This dataset is used to generate subsets of data, such as shown in Fig. 2c of the main text, Supplementary Fig. 4, Supplementary Figs. 8c,d, Supplementary Fig. 10c.

The data of the nuclear spin temperature measurements can be found in several files named `SpinT(QDn,Isotope,Bz=x).txt`, where "QDn" in the filename is the label of the individual quantum dot between QD1 and QD3, "Isotope" is either ^{69}Ga or ^{75}As, and "Bz=x" gives the numeric value x of the static magnetic field, which is either 4 or 10 T. Each file consists of pairs of columns of data. For each pair, the first column is the measured value, while the second column is half of the confidence interval for this value. All data values are in μeV. The column header labels the type of the measurement, which is "NoRf" for a measurement without any radiofrequency excitation, "Comb" for a selective frequency comb saturation, "Swp" for an adiabatic sweep measurement. For the last two types, the type of the measurement is followed by a list of the NMR transitions that are excited by the radiofrequency. The list is given in curly brackets {}, and can include "CT" for the central transition $-1/2 \leftrightarrow +1/2$ and "STL"("STH") for the low-frequency (high-frequency) quadrupolar satellite transition. For ^{69}Ga, the low-frequency satellite corresponds to the $-3/2 \leftrightarrow +1/2$ NMR transition, while for ^{75}As it corresponds to the $+1/2 \leftrightarrow +3/2$ NMR transition. These datasets are used to generate subsets of data, such as shown in Fig. 3d of the main text, Supplementary Fig. 19, and Supplementary Fig. 20.

The data for the maximum positive and minimum negative hyperfine shifts shown for individual dots QD1 - QD12 in Fig. 3f of the main text can be found in file `Fig3f.txt`. Each line corresponds to an individual QD and all values are in μeV.

The data of the nuclear spin buildup dynamics (Fig. 4a of the main text) measured under σ^+ and σ^- optical pumping can be found in files `Fig4a(sigma+).txt` and `Fig4a(sigma+).txt`, respectively. The first column is the pumping time T_{Pump} in s, the second column is the hyperfine shift E_{hf} in μeV.

The data of the nuclear spin relaxation dynamics (Fig. 4b of the main text) can be found in file `Fig4b.txt`. The first column is the dark time T_{Dark} in s. The subsequent columns are the hyperfine

shift E_{hf} in μeV. Each column corresponds to a fixed duration of the second Rf pulse $T_{\mathrm{Rf},2}$ used to control the degree of the initial nuclear spin polarization. The value of $T_{\mathrm{Rf},2}$ in s is given in square brackets at the top of each column. Values that are missing for a particular combination of T_{Dark} and $T_{\mathrm{Rf},2}$ are shown with "-".

Nuclear spin relaxation times $T_{1,\mathrm{N}}$ as a function of the initial hyperfine shift E_{hf} (Fig. 4c of the main text) can be found in files `Fig4c(QD2).txt` and `Fig4c(QD3).txt` for QD2 and QD3, respectively. The first column in each file is the initial E_{hf} in μeV, the second column is half the confidence interval of the initial E_{hf}, also in μeV. The third column is $T_{1,\mathrm{N}}$ in s, with its half confidence interval given in the fourth column.

The calibration data of the optical polarization of the pump (Supplementary Fig. 5) can be found in files `FigS5(sigma+).txt` and `FigS5(sigma-).txt` for optical pumping close to σ^{+} and σ^{-}, respectively. The first column is the rotation angle of the half-wave plate in degrees, the second column is the hyperfine shift E_{hf} in μeV.

---

[1] A. Oshiyama and S. Ohnishi, DX center: Crossover of deep and shallow states in Si-doped $\mathrm{Al_xGa_{1-x}As}$, Phys. Rev. B **33**, 4320 (1986).

[2] P. M. Mooney, Deep donor levels (DX centers) in III-V semiconductors, Journal of Applied Physics **67**, R1 (1990).

[3] L. Zhai, M. C. Löbl, G. N. Nguyen, J. Ritzmann, A. Javadi, C. Spinnler, A. D. Wieck, A. Ludwig, and R. J. Warburton, Low-noise GaAs quantum dots for quantum photonics, Nat. Commun. **11**, 4745 (2020).

[4] C. Heyn, A. Stemmann, T. Koppen, C. Strelow, T. Kipp, M. Grave, S. Mendach, and W. Hansen, Highly uniform and strain-free GaAs quantum dots fabricated by filling of self-assembled nanoholes, Appl. Phys. Lett. **94**, 183113 (2009).

[5] P. Atkinson, E. Zallo, and O. G. Schmidt, Independent wavelength and density control of uniform GaAs/AlGaAs quantum dots grown by infilling self-assembled nanoholes, J. Appl. Phys. **112**, 054303 (2012).

[6] B. Urbaszek, X. Marie, T. Amand, O. Krebs, P. Voisin, P. Maletinsky, A. Högele, and A. Imamoğlu, Nuclear spin physics in quantum dots: An optical investigation, Rev. Mod. Phys. **85**, 79 (2013).

[7] Y. El Khalifi, B. Gil, H. Mathieu, T. Fukunaga, and H. Nakashima, Dependence of the light-hole—heavy-hole splitting on layer thickness and substrate orientation in GaAs-(GaAl) As single quantum wells, Phys. Rev. B **39**, 13533 (1989).

[8] V. B. Timofeev, M. Bayer, A. Forchel, and M. Potemski, Mixing of excitonic states containing light and heavy holes in an isolated GaAs/AlGaAs quantum well in a magnetic field, Journal of Experimental

and Theoretical Physics Letters **64**, 57 (1996).

[9] E. A. Chekhovich, M. M. Glazov, A. B. Krysa, M. Hopkinson, P. Senellart, A. Lemaître, M. S. Skolnick, and A. I. Tartakovskii, Element-sensitive measurement of the hole-nuclear spin interaction in quantum dots, Nat. Phys. **9**, 74 (2013).

[10] C. P. Slichter, *Principles of Magnetic Resonance* (Springer, 1990).

[11] C. Bulutay, E. A. Chekhovich, and A. I. Tartakovskii, Nuclear magnetic resonance inverse spectra of InGaAs quantum dots: Atomistic level structural information, Phys. Rev. B **90**, 205425 (2014).

[12] Y. Huang, Y. Puttisong, S. Filippov, I. Buyanova, and W. Chen, Oblique nuclear quadrupole interaction in self-assembled structures based on semiconductor quantum dots, Phys. Rev. Appl. **14**, 044019 (2020).

[13] E. A. Chekhovich, A. Ulhaq, E. Zallo, F. Ding, O. G. Schmidt, and M. S. Skolnick, Measurement of the spin temperature of optically cooled nuclei and GaAs hyperfine constants in GaAs/AlGaAs quantum dots, Nat. Mater. **16**, 982 (2017).

[14] J. M. Taylor, C. M. Marcus, and M. D. Lukin, Long-lived memory for mesoscopic quantum bits, Phys. Rev. Lett. **90**, 206803 (2003).

[15] P. Millington-Hotze, S. Manna, S. F. Covre da Silva, A. Rastelli, and E. A. Chekhovich, Nuclear spin diffusion in the central spin system of a GaAs/AlGaAs quantum dot, Nature Communications **14**, 2677 (2023).

[16] D. Huber, B. U. Lehner, D. Csontosová, M. Reindl, S. Schuler, S. F. Covre da Silva, P. Klenovský, and A. Rastelli, Single-particle-picture breakdown in laterally weakly confining GaAs quantum dots, Phys. Rev. B **100**, 235425 (2019).

[17] X. Yuan, F. Weyhausen-Brinkmann, J. Martín-Sánchez, G. Piredda, V. Křápek, Y. Huo, H. Huang, C. Schimpf, O. G. Schmidt, J. Edlinger, G. Bester, R. Trotta, and A. Rastelli, Uniaxial stress flips the natural quantization axis of a quantum dot for integrated quantum photonics, Nat. Commun. **9**, 3058 (2018).

[18] Y. H. Huo, A. Rastelli, and O. G. Schmidt, Ultra-small excitonic fine structure splitting in highly symmetric quantum dots on GaAs (001) substrate, Appl. Phys. Lett. **102**, 152105 (2013).

[19] A. V. Koudinov, I. A. Akimov, Y. G. Kusrayev, and F. Henneberger, Optical and magnetic anisotropies of the hole states in Stranski-Krastanov quantum dots, Phys. Rev. B **70**, 241305 (2004).

[20] M. Gawełczyk, M. Syperek, A. Maryński, P. Mrowiński, L. Dusanowski, K. Gawarecki, J. Misiewicz, A. Somers, J. P. Reithmaier, S. Höfling, and G. Sek, Exciton lifetime and emission polarization dispersion in strongly in-plane asymmetric nanostructures, Phys. Rev. B **96**, 245425 (2017).

[21] A. M. Waeber, M. Hopkinson, I. Farrer, D. A. Ritchie, J. Nilsson, R. M. Stevenson, A. J. Bennett, A. J. Shields, G. Burkard, A. I. Tartakovskii, M. S. Skolnick, and E. A. Chekhovich, Few-second-long correlation times in a quantum dot nuclear spin bath probed by frequency-comb nuclear magnetic resonance spectroscopy, Nat. Phys. **12**, 688 (2016).

[22] F. Bloch, Nuclear induction, Phys. Rev. **70**, 460 (1946).

[23] E. A. Chekhovich, K. V. Kavokin, J. Puebla, A. B. Krysa, M. Hopkinson, A. D. Andreev, A. M.

Sanchez, R. Beanland, M. S. Skolnick, and A. I. Tartakovskii, Structural analysis of strained quantum dots using nuclear magnetic resonance, Nat. Nanotechnol. **7**, 646 (2012).

[24] B. Urbaszek, P.-F. Braun, T. Amand, O. Krebs, T. Belhadj, A. Lemaítre, P. Voisin, and X. Marie, Efficient dynamical nuclear polarization in quantum dots: Temperature dependence, Phys. Rev. B **76**, 201301 (2007).

[25] C. Schimpf, M. Reindl, P. Klenovský, T. Fromherz, S. F. Covre Da Silva, J. Hofer, C. Schneider, S. Höfling, R. Trotta, and A. Rastelli, Resolving the temporal evolution of line broadening in single quantum emitters, Opt. Express **27**, 35290 (2019).

[26] J. M. Villas-Bôas, S. E. Ulloa, and A. O. Govorov, Decoherence of Rabi oscillations in a single quantum dot, Phys. Rev. Lett. **94**, 057404 (2005).

[27] S. Adachi, *Properties of Semiconductor Alloys: Group-IV, III-V and II-VI Semiconductors* (Wiley, 2009).

[28] A. Imamoḡlu, E. Knill, L. Tian, and P. Zoller, Optical pumping of quantum-dot nuclear spins, Phys. Rev. Lett. **91**, 017402 (2003).

[29] H. Christ, J. I. Cirac, and G. Giedke, Quantum description of nuclear spin cooling in a quantum dot, Phys. Rev. B **75**, 155324 (2007).

[30] G. Gillard, E. Clarke, and E. A. Chekhovich, Harnessing many-body spin environment for long coherence storage and high-fidelity single-shot qubit readout, Nature Communications **13**, 4048 (2022).

[31] H. Huang, D. Csontosová, S. Manna, Y. Huo, R. Trotta, A. Rastelli, and P. Klenovský, Electric field induced tuning of electronic correlation in weakly confining quantum dots, Phys. Rev. B **104**, 165401 (2021).

[32] X. Xu, W. Yao, B. Sun, D. G. Steel, A. S. Bracker, D. Gammon, and L. J. Sham, Optically controlled locking of the nuclear field via coherent dark-state spectroscopy, Nature **459**, 1105 (2009).

[33] D. A. Gangloff, L. Zaporski, J. H. Bodey, C. Bachorz, D. M. Jackson, G. Éthier-Majcher, C. Lang, E. Clarke, M. Hugues, C. Le Gall, and M. Atatüre, Witnessing quantum correlations in a nuclear ensemble via an electron spin qubit, Nat. Phys. **17**, 1247 (2021).

[34] D. M. Jackson, U. Haeusler, L. Zaporski, J. H. Bodey, N. Shofer, E. Clarke, M. Hugues, M. Atatüre, C. Le Gall, and D. A. Gangloff, Optimal purification of a spin ensemble by quantum-algorithmic feedback, Phys. Rev. X **12**, 031014 (2022).

[35] A. V. Khaetskii, D. Loss, and L. Glazman, Electron Spin Decoherence in Quantum Dots due to Interaction with Nuclei, Phys. Rev. Lett. **88**, 186802 (2002).

[36] M. Goldman, *Spin temperature and nuclear magnetic resonance in solids* (Oxford University Press, Oxford, 1970).

[37] T. A. Knuuttila, J. T. Tuoriniemi, K. Lefmann, K. I. Juntunen, F. B. Rasmussen, and K. K. Nummila, Polarized nuclei in normal and superconducting rhodium, Journal of Low Temperature Physics **123**, 65 (2001).

[38] W. T. Wenckebach, The solid effect, Applied Magnetic Resonance **34**, 227 (2008).

[39] C. Kloeffel and D. Loss, Prospects for spin-based quantum computing in quantum dots, Annual Review of Cond. Matt. Phys. **4**, 51 (2013).

[40] G. Ragunathan, *Nuclear Spin Phenomena in III-V and II-VI Semiconductor Quantum Dots*, Ph.D. thesis, University of Sheffield (2019).

[41] L. Zaporski, N. Shofer, J. H. Bodey, S. Manna, G. Gillard, M. H. Appel, C. Schimpf, S. F. Covre da Silva, J. Jarman, G. Delamare, G. Park, U. Haeusler, E. A. Chekhovich, A. Rastelli, D. A. Gangloff, M. Atatüre, and C. Le Gall, Ideal refocusing of an optically active spin qubit under strong hyperfine interactions, Nat. Nanotechnol. **18**, 257 (2023).

[42] W. Janzen, Adiabatic rapid passage NMR signal shape and passage conditions in solids, Journal of Magnetic Resonance (1969) **12**, 71 (1973).

[43] M. Goldman, J. Jacquinot, M. Chapellier, and V. H. Chau, Nonlinear effects in spin temperature, Journal of Magnetic Resonance (1969) **18**, 22 (1975).

[44] G. Cowan, *Statistical Data Analysis* (Clarendon Press, 1998).
