# Supplementary material for: Approaching a fully-polarized state of nuclear spins in a solid
Source: Nat Commun. 2024 Feb 2;15:985. doi: 10.1038/s41467-024-45364-2 (PMC10837425; doi:10.1038/s41467-024-45364-2)
Supplement: Supplementary file 3 — Description of Additional Supplementary Files [file 41467_2024_45364_MOESM3_ESM.docx]

File Name: Supplementary Data 1

Description: Derivation of Equation S15. This is a notebook with the code that can be evaluated using Wolfram Mathematica software.

File Name: Supplementary Data 2

Description: Derivation of Equation S15. This is a portable document format (pdf) version of Supplementary Data 1.
