# Supplementary material for: Approaching a fully-polarized state of nuclear spins in a solid
Source: Nat Commun. 2024 Feb 2;15:985. doi: 10.1038/s41467-024-45364-2 (PMC10837425; doi:10.1038/s41467-024-45364-2)
Supplement: Supplementary file 5 — Supplementary Data 2 [file 41467_2024_45364_MOESM5_ESM.pdf]

File description

Supplementary file for “Approaching a fully-polarized state of nuclear spins in a solid” by Peter Millington-Hotze, Harry E. Dyte, Santanu Manna, Saimon F. Covre da Silva, Armando Rastelli, and Evgeny A. Chekhovich (2023).

This file was generated using Wolfram Mathematica software, version:

In[ ]:=
```
$Version
```

Out[ ]=
```
13.2.0 for Microsoft Windows (64-bit) (November 18, 2022)
```

Analytical derivations

Calculation of the variance of polarization of an ensemble of nuclear spins.

An ensemble of identical nuclear spins is considered. The number of spins in the ensemble is taken to be NSpins. Each nucleus has a spin quantum number INuc. The possible nuclear spin projections onto the z-axis are Iz ∊ [-INuc,+INuc]. For INuc = 3/2, which is the case for Ga and As nuclei, the possible spin projections are Iz = -3/2, -1/2, +1/2, +3/2.

The key assumption is that each nuclear spin is polarized independently of all other nuclear spins. Upon dynamical nuclear polarization via the central electron spin, the z-projection of each nuclear spin is assumed to be described by an independent Boltzmann distribution.

```
Clear[NSpins]
```

The probability p[Iz] that one spin INuc occupies a state with a defined z-projection Iz is given by the Boltzmann distribution, where β is the dimensionless inverse spin temperature.

In[∘]:=
```
pBoltzmann = (p[Iz_] → Exp[β * Iz] / FullSimplify[Sum[Exp[β * Iz], {Iz, -INuc, INuc}]])
```

Out[∘]=

$$p[Iz\_] \rightarrow \frac{e^{Iz\,\beta}}{\text{Cosh}[INuc\,\beta] + \text{Coth}\left[\frac{\beta}{2}\right]\text{Sinh}[INuc\,\beta]}$$

Mean (expectation) Iz for one spin:

In[∘]:=
```
MeanBoltzmann = FullSimplify[Sum[Iz * p[Iz] /. pBoltzmann, {Iz, -INuc, INuc}]]
```

Out[∘]=

$$\frac{(1 + INuc)\,\text{Sinh}[INuc\,\beta] - INuc\,\text{Sinh}[(1 + INuc)\,\beta]}{\text{Cosh}[INuc\,\beta] - \text{Cosh}[(1 + INuc)\,\beta]}$$

Mean (expectation) of the polarization degree PN for one spin is the mean (expectation) Iz normalized by INuc. Example for spin-3/2.

In[∘]:=
```
FullSimplify[(MeanBoltzmann / INuc /. INuc → 3 / 2)]
Plot[%, {β, -10, 10}, Frame → True, FrameLabel → {"Dimensionless inverse spin temperature, β", "Polarization degree, PN"}]
```

Out[∘]=

$$\frac{1}{3}\left(\text{Tanh}\left[\frac{\beta}{2}\right] + 2\,\text{Tanh}[\beta]\right)$$

Out[∘]=

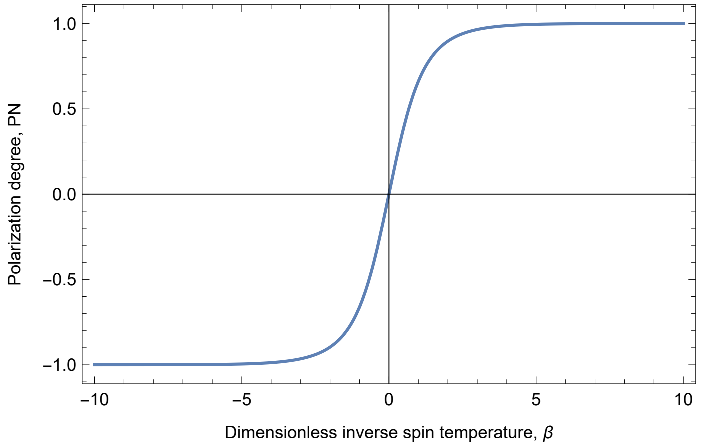

Polarization of an ensemble of N nuclear spins is described by a multivariate Boltzmann distribution. This is equivalent to assuming that individual nuclear spins are polarized by the central electron independently. The mean (ensemble-average expectation) z-projection of the nuclear spin

ensemble is described by the random variable IzMean. By definition, -INuc ≤ IzMean ≤ +INuc.
IzMean =

In[•]:= **Sum[Iz[i], {i, 1, NSpins}] / NSpins**

Out[•]=

$$\frac{\sum_{i=1}^{NSpins} Iz[i]}{NSpins}$$

The probability to find a particular microscopic configuration of the nuclear spin ensemble (i.e. a particular permutation of Iz[i], where index i∈ [1,NSpins] runs over all nuclear spins in the ensemble) is given by the product of the single-spin probabilities. This product is:

In[•]:= **Product[p[Iz[i]], {i, 1, NSpins}]**

Out[•]=

$$\prod_{i=1}^{NSpins} p[Iz[i]]$$

The variance of the mean Iz projection (IzMean) over the ensemble is by definition given by the following expectation value:

In[•]:= **Expectation[(IzMean - MeanBoltzmann)^2] /. {IzMean → Sum[Iz[i], {i, 1, NSpins}] / NSpins}**

••• Expectation: Expectation called with 1 argument; 2 or more arguments are expected. ⓘ

••• Expectation: Expectation called with 1 argument; 2 or more arguments are expected. ⓘ

Out[•]=

$$\text{Expectation}\left[\left(-\frac{(1+INuc)\,Sinh[INuc\,\beta]-INuc\,Sinh[(1+INuc)\,\beta]}{Cosh[INuc\,\beta]-Cosh[(1+INuc)\,\beta]}+\frac{\sum_{i=1}^{NSpins} Iz[i]}{NSpins}\right)^2\right]$$

This expectation can be found as a sum over all Iz[i] permutations (microstates), weighted by their probabilities:
Sum[p[IzPermutation] * (IzMean[IzPermutation] - MeanBoltzmann)^2, {IzPermutation}],
where p[IzPermutation] is the probability of a particular microscopic configuration (permutation of the individual Iz).

This expectation can also be written as $\frac{V[NSpins]}{NSpins^2}$, where V[NSpins] is the variance of the total Iz of the ensemble of NSpins nuclei (rather than the variance of the ensemble-average mean Iz, which ranges between -INuc ≤ IzMean ≤ +INuc).

The variance of the mean (ensemble-average) Iz can be calculated as follows.

Expanding the sum in the definition of the expectation value we have:

$$\frac{V[NSpins]}{NSpins^2} = Sum\left[p[Iz[1]] * p[Iz[2]] * ... * p[Iz[NSpins]] * \left(\frac{(Iz[1]+Iz[2]+...+Iz[NSpins])}{NSpins} - MeanBoltzmann\right)^2, \{Iz[1], -INuc, INuc\}, \{Iz[2], -INuc, INuc\}, ..., \{Iz[NSpins], -INuc, INuc\}\right]$$

where the summation goes over all possible spin z-projections Iz[1], Iz[2],...Iz[NSpins] of all the individual nuclei, and p[Iz[i]] is the probability to find the i-th individual spin in a state with z-projection Iz[i].

The above can be rewritten, taking out $\frac{1}{NSpins^2}$ as a common factor:

$$\frac{V[NSpins]}{NSpins^2} = \frac{1}{NSpins^2} * Sum\left[p[Iz[1]] * p[Iz[2]] * ... * p[Iz[NSpins]] * ((Iz[1] + Iz[2] + ... + Iz[NSpins]) - NSpins * MeanBoltzmann)^2, \{Iz[1], -INuc, INuc\}, \{Iz[2], -INuc, INuc\}, ..., \{Iz[NSpins], -INuc, INuc\}\right]$$

We further rewrite the expression in terms of the deviations (Iz[i]-MeanBoltzmann) of the spin projections from their mean (their expectation value). In this way, we regroup the squared sum in order to eliminate the NSpins*MeanBoltzmann product:

$$\frac{V[NSpins]}{NSpins^2} = \frac{1}{NSpins^2} * Sum\left[p[Iz[1]] * p[Iz[2]] * ... * p[Iz[NSpins]] * ((Iz[1] - MeanBoltzmann) + (Iz[2] - MeanBoltzmann) + ... + (Iz[NSpins] - MeanBoltzmann))^2, \{Iz[1], -INuc, INuc\}, \{Iz[2], -INuc, INuc\}, ..., \{Iz[NSpins], -INuc, INuc\}\right]$$

Expansion of the square in the sum gives two types of terms:
- Squared deviations for individual nuclei, $(Iz[i] - MeanBoltzmann)^2$
- Product terms 2(Iz[i]-MeanBoltzmann)(Iz[j]-MeanBoltzmann) for distinct pairs of nuclei i≠j

The expectation value of each deviation (Iz[i]-MeanBoltzmann) is 0. The deviations of the different nuclei are independent. Consequently, the expectation of the product of the deviations is the product of expectations, which is 0. This result can also be verified directly. The expectation of the deviation for any one spin is:

```
Simplify[Sum[(p[Iz[i]] /. pBoltzmann) * (Iz[i] - MeanBoltzmann), {Iz[i], -INuc, INuc}], NSpins > 1 && INuc > 1 / 2 && β ∈ Reals]
```

Out[•]=

0

Direct calculation of the expectation of a product for a pair of nuclei i ≠ j. The summation goes over only Iz[i] and Iz[j]. The summation over other Iz[k] simply gives the total of probabilities p[Iz[k]] as a factor, and this total is 1. Direct calculation of the expectation of the product:

```
Simplify[ExpandAll[
  Sum[ExpandAll[(p[Iz[i]] /. pBoltzmann) * (p[Iz[j]] /. pBoltzmann) * 2 * (Iz[i] - MeanBoltzmann) * (Iz[j] - MeanBoltzmann)],
   {Iz[i], -INuc, INuc}, {Iz[j], -INuc, INuc}]], NSpins > 1 && INuc > 1 / 2 && β ∈ Reals]
```

Out[•]=

0

Thus, only the terms with the squares of the deviations (Iz[*i*] – MeanBoltzmann)^2 contribute to the variance:

$$\frac{V[\text{NSpins}]}{\text{NSpins}^2} = \frac{1}{\text{NSpins}^2} * \text{Sum}\Big[p[\text{Iz}[1]] * p[\text{Iz}[2]] * ... * p[\text{Iz}[\text{NSpins}]] * \left((\text{Iz}[1] - \text{MeanBoltzmann})^2 + (\text{Iz}[2] - \text{MeanBoltzmann})^2 + ... + (\text{Iz}[\text{NSpins}] - \text{MeanBoltzmann})^2\right), \{\text{Iz}[1], -\text{INuc}, \text{INuc}\}, \{\text{Iz}[2], -\text{INuc}, \text{INuc}\}, ..., \{\text{Iz}[\text{NSpins}], -\text{INuc}, \text{INuc}\}\Big]$$

The contribution of the first nuclear spin is:

$$\frac{1}{\text{NSpins}^2} * \text{Sum}\Big[p[\text{Iz}[1]] * p[\text{Iz}[2]] * ... * p[\text{Iz}[\text{NSpins}]] * (\text{Iz}[1] - \text{MeanBoltzmann})^2, \{\text{Iz}[1], -\text{INuc}, \text{INuc}\}, \{\text{Iz}[2], -\text{INuc}, \text{INuc}\}, ..., \{\text{Iz}[\text{NSpins}], -\text{INuc}, \text{INuc}\}\Big]$$

The sums over all Iz[i] except Iz[1] are trivial, since they simply give the total of the probabilities Sum[p[Iz[i]],{Iz[i],-INuc,INuc}] as a factor, and this totaled probability is 1. Thus, the contribution of the first spin simplifies to:

$$\frac{1}{\text{NSpins}^2} * \text{Sum}\Big[p[\text{Iz}[1]]\,(\text{Iz}[1] - \text{MeanBoltzmann})^2, \{\text{Iz}[1], -\text{INuc}, \text{INuc}\}\Big]$$

The explicit form of this sum is:

```
1/NSpins^2 *
 FullSimplify[Sum[(p[Iz[1]] /. pBoltzmann) * (Iz[1] - MeanBoltzmann)^2, {Iz[1], -INuc, INuc}], NSpins > 1 && INuc > 1 / 2 && β ∈ Reals]
```

Out[•]=

$$-\frac{e^{\beta}\left(-4\,\text{INuc}\,(1+\text{INuc}) + (1+2\,\text{INuc})^2 \text{Cosh}[\beta] - \text{Cosh}[\beta + 2\,\text{INuc}\,\beta]\right) \text{Csch}\left[\left(\frac{1}{2}+\text{INuc}\right)\beta\right]^2}{2\left(-1+e^{\beta}\right)^2 \text{NSpins}^2}$$

The contribution of all other spins are identical. Thus the total variance is the variance originating from the first spin, times the number of spins NSpins.

Main result. The variance of the ensemble-average mean of the Iz polarization is:

```
VarianceBoltzmann = FullSimplify[NSpins * 1/NSpins^2 * Sum[(p[Iz[1]] /. pBoltzmann) * (Iz[1] - MeanBoltzmann)^2, {Iz[1], -INuc, INuc}],
  NSpins > 1 && INuc > 1 / 2 && β ∈ Reals]
```

Out[ ]=

$$\frac{\mathrm{Csch}\left[\frac{\beta}{2}\right]^2 - (1 + 2\,\mathrm{INuc})^2\,\mathrm{Csch}\left[\left(\frac{1}{2} + \mathrm{INuc}\right)\beta\right]^2}{4\,\mathrm{NSpins}}$$

The variance of the total Iz (rather than of the ensemble-average mean Iz) is found by multiplying the above result by NSpins^2:

In[ ]:=
```
FullSimplify[NSpins^2 * VarianceBoltzmann]
```

Out[ ]=

$$\frac{1}{4}\,\mathrm{NSpins}\left(\mathrm{Csch}\left[\frac{\beta}{2}\right]^2 - (1 + 2\,\mathrm{INuc})^2\,\mathrm{Csch}\left[\left(\frac{1}{2} + \mathrm{INuc}\right)\beta\right]^2\right)$$

The variance of the total Iz characterizes the uncertainty of the total nuclear spin magnetization. This uncertainty is responsible for the dephasing of the central electron spin:

Numerical verification

Once again, we assume that each nuclear spin is polarized independently, and that Iz of each spin follows the Boltzmann distribution. The standard deviation of the polarization degree as a function of the mean polarization degree can be calculated numerically by randomly sampling the Boltzmann distribution.

```
(*Number of nuclei NSpins, numerical value*)
nNSpins = 100000;

(*Number of different random polarization configurations of the nuclear spin ensemble*)
NRndTrials = 10000;

(*Spin quantum number, numerical value*)
nINuc = 3 / 2;
```

This function calculates n trial implementations (rolls of a dice) of a Boltzmann distribution. For each trial, a sample is drawn from the possible Iz∈ [-INuc,INuc] spin projections.

```
In[•]:= Clear[fRndSample];
fRndSample[β_, INuc_, n_(*Number of samples, which will be the number of nuclei*)] :=
 RandomChoice[(Table[Exp[β * Iz], {Iz, -INuc, INuc}]) → Table[Iz, {Iz, -INuc, INuc}], n]
```

Analytical result derived above

In[•]:= $$\text{VarianceBoltzmann} = \frac{\text{Csch}\left[\frac{\beta}{2}\right]^2 - (1 + 2\,\text{INuc})^2\,\text{Csch}\left[\left(\frac{1}{2} + \text{INuc}\right)\beta\right]^2}{4\,\text{NSpins}};$$

Some specific examples of the variance V of the total ensemble Iz spin projection at different polarization degrees PN. Using the analytical result and NSpins == 10^5, which corresponds to the number of nuclear spins in a typical epitaxial GaAs/AlGaAs quantum dot.

In[ ]:=
```
Map[{"PN" → #, βPN = (β /. FindRoot[(MeanBoltzmann/INuc /. INuc → 3 / 2) == #, {β, 0.1}]); β → βPN,
    "V" → NSpins * (*Sqrt to calculate std deviation from dispersion (variance)*) Sqrt[VarianceBoltzmann] /.
     {INuc → 3 / 2, β → βPN, NSpins → 10^5}} &, {1. * 10^-3, 0.1, 0.5, 0.9, 0.95, 0.99}] // MatrixForm
```

Out[ ]//MatrixForm=

PN → 0.001	β → 0.0012	V → 353.553
PN → 0.1	β → 0.120493	V → 351.385
PN → 0.5	β → 0.673996	V → 296.37
PN → 0.9	β → 2.03001	V → 130.11
PN → 0.95	β → 2.66141	V → 89.6402
PN → 0.99	β → 4.21458	V → 39.0185

Calculate numerically the ensemble-average mean polarization degree and the standard deviation of the mean polarization for different spin temperatures β. Computation with a large number of random samples is time-consuming, hence using parallelization.

```
AbsoluteTiming[
 ExpMeanStdDevTab = ParallelTable[Join[{β}, ({(*Polarization degree*)1. * Mean[#]/NINuc, (*Standard deviation normalized by spin
        number INuc and 1/ Sqrt[NSpins]. INuc converts it into polarization degree, and 1/ Sqrt[NSpins] removes the
        explicit dependence on NSpins*)1. * StandardDeviation[#]/(NINuc * (1/ Sqrt[nNSpins]))} &) @ (*NRndTrials implementations of Mean Iz*)
      Table[(*Mean Iz over NSpins spins*)Mean[fRndSample[β, NINuc, nNSpins]], NRndTrials]], {β, -10, 10, 0.049}];]
```

Out[ ]=

```
{8373.34, Null}
```

In[ ]:=
```
ListPlot[{ExpMeanStdDevTab[[All, {1, 2}]], Map[{#[[1]], (MeanBoltzmann/INuc /. INuc → 3 / 2) /. β → #[[1]]} &, ExpMeanStdDevTab]},
 Frame → True, FrameLabel → {"Dimensionless inverse spin temperature, β", "Nuclear spin polarization degree, PN"}]
```

```
ListLogPlot[{ExpMeanStdDevTab[[All, {2, 3}]],
  Map[{#[[2]], Sqrt[VarianceBoltzmann] / ((*Same normalization as for numerics*) (3 / 2) * (1 / Sqrt[nNSpins])) /. {INuc → 3 / 2, β → #[[1]], NSpins → nNSpins}} &,
   ExpMeanStdDevTab]}, Frame → True, FrameLabel → {"Polarization degree, PN",
   "Standard deviation (fluctuation) of PN, normalized by Sqrt[NSpins]\n to remove explicit dependence on NSpins"},
 PlotLegends → {"Numerics", "Exact analytical derivation"}, ImageSize → 600]

ListPlot[
 Map[{#[[2]], #[[3]] - Sqrt[VarianceBoltzmann] / ((*Same normalization as for numerics*) (3 / 2) * (1 / Sqrt[nNSpins])) /. {INuc → 3 / 2, β → #[[1]], NSpins → nNSpins}} &,
  ExpMeanStdDevTab], Frame → True, FrameLabel → {"Polarization degree, PN",
   "Residual difference between the numerical and the analytical results\n for the standard deviation of PN"},
 PlotRange → All, ImageSize → 600]

ListLogLogPlot[{
  Map[{1 - #[[2]], #[[3]]} &, ExpMeanStdDevTab],
  Map[{1 - #[[2]], Sqrt[1 - #[[2]]^2]} &, ExpMeanStdDevTab],
  Map[{1 - #[[2]], Sqrt[VarianceBoltzmann] / ((*Same normalization as for numerics*) (3 / 2) * (1 / Sqrt[nNSpins])) /. {INuc → 3 / 2, β → #[[1]], NSpins → nNSpins}} &,
   ExpMeanStdDevTab]}, Frame → True,
 FrameLabel → {"Deviation from full polarization, 1-PN", "Standard deviation of PN, normalized by Sqrt[NSpins]"},
 PlotLegends → {"Numerics", "Sqrt[1 - PN^2]", "Exact analytical derivation"}, ImageSize → 600, Joined → True]
```

Out[ ]=

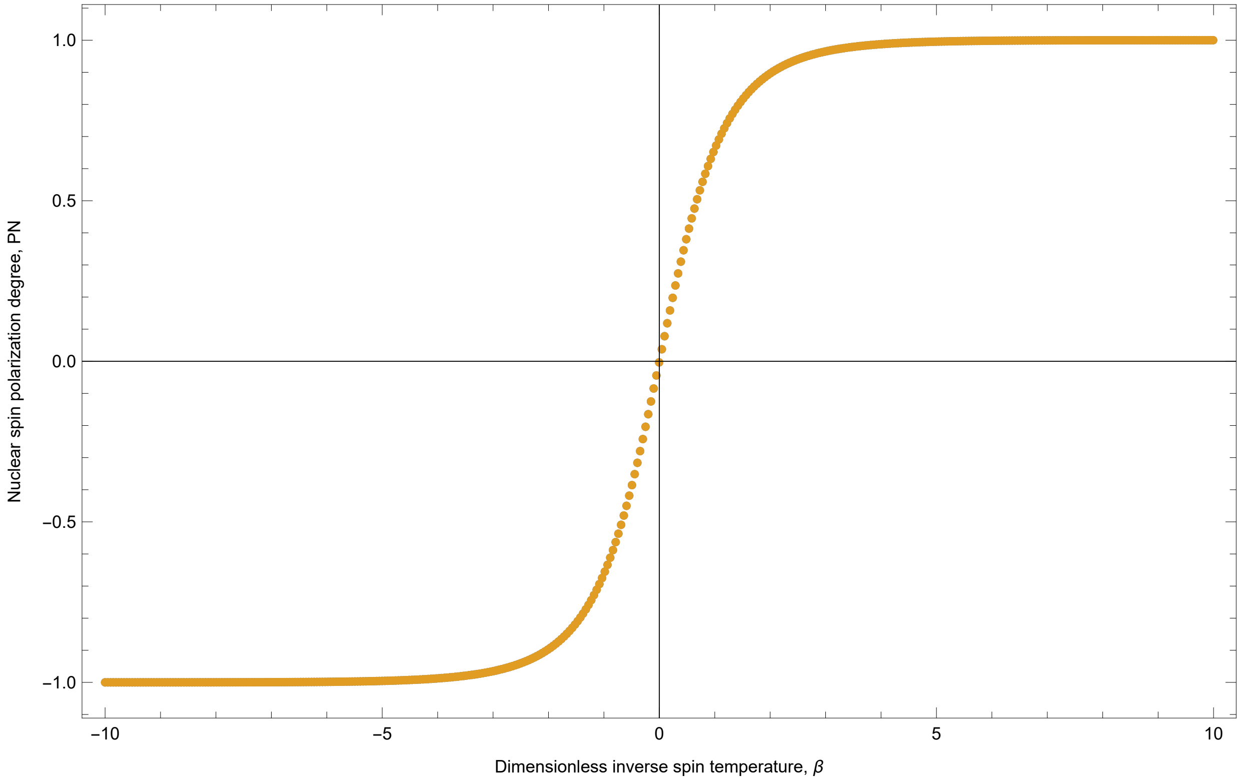

1.0
0.5
0.0
−0.5
−1.0
−10
−5
0
5
10
Nuclear spin polarization degree, PN
Dimensionless inverse spin temperature, β

Out[ ]=

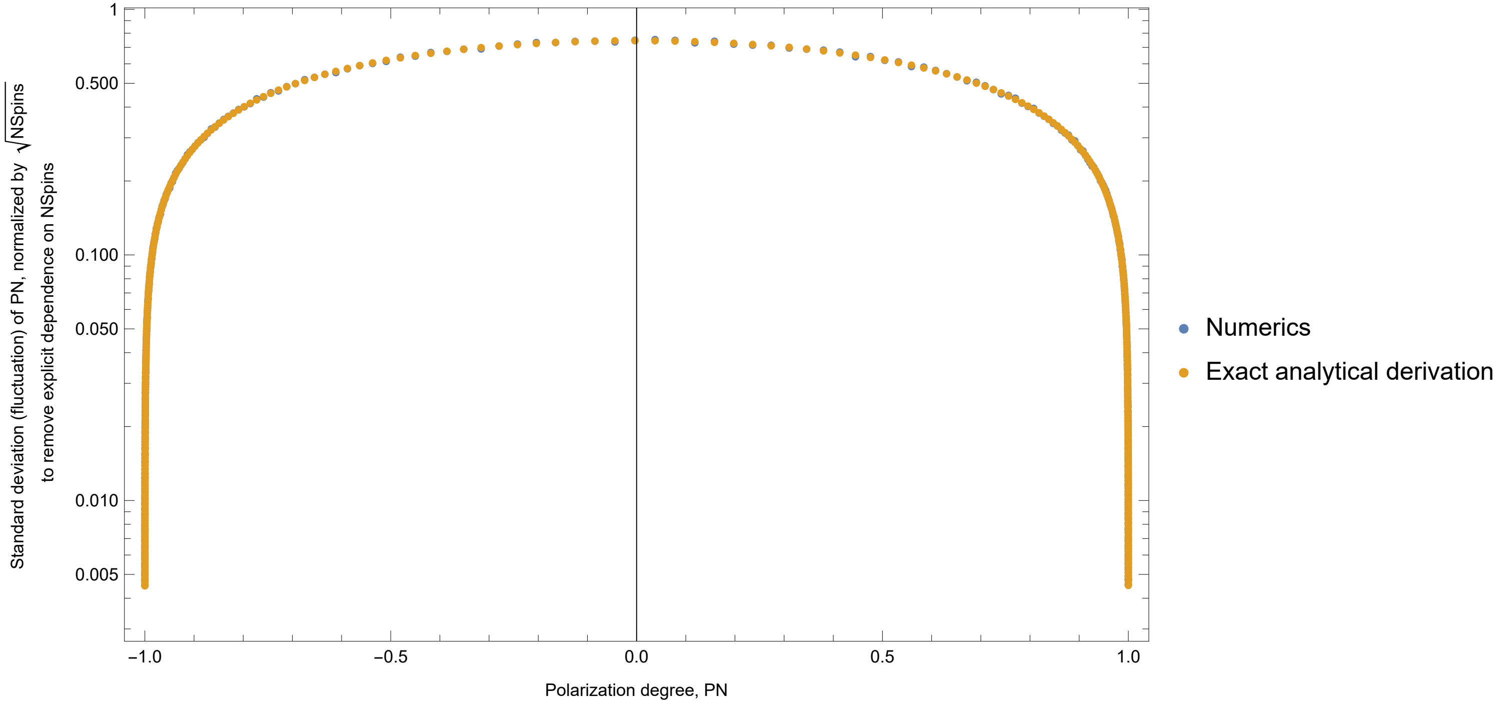
1
0.500
0.100
0.050
0.010
0.005
Standard deviation (fluctuation) of PN, normalized by √NSpins
to remove explicit dependence on NSpins
−1.0
−0.5
0.0
0.5
1.0
Polarization degree, PN
Numerics
Exact analytical derivation

Out[ ]=

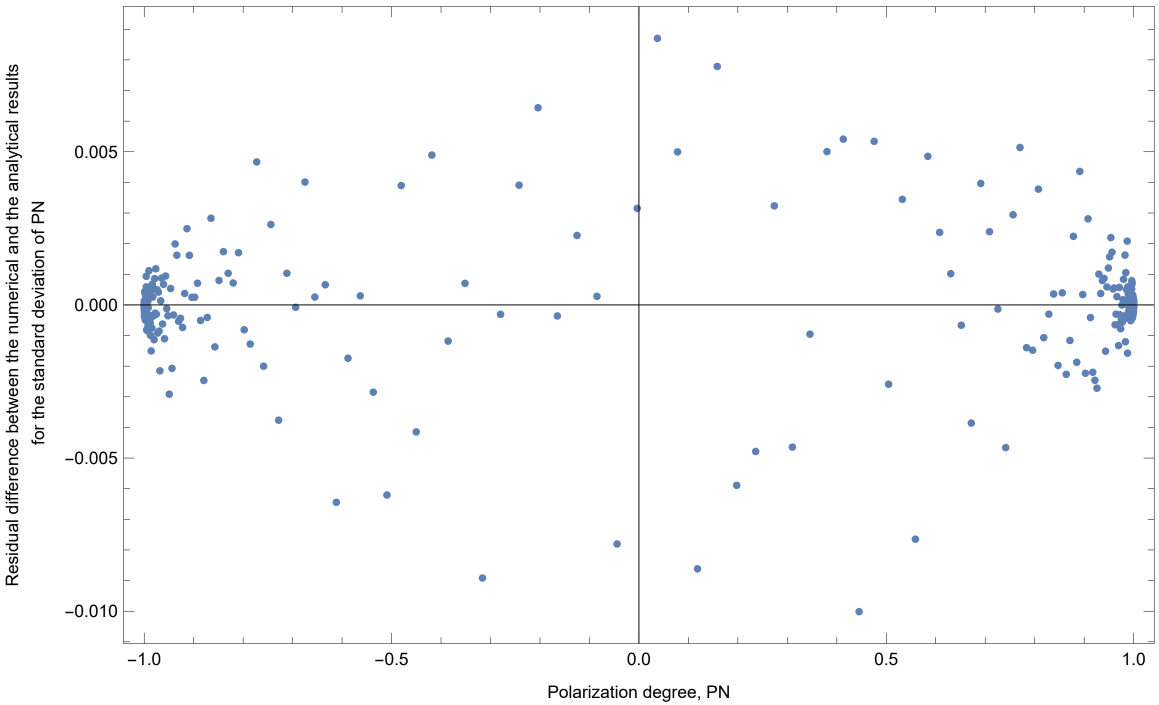

Residual difference between the numerical and the analytical results for the standard deviation of PN
0.005
0.000
−0.005
−0.010
−1.0
−0.5
0.0
0.5
1.0
Polarization degree, PN

Out[ ]=

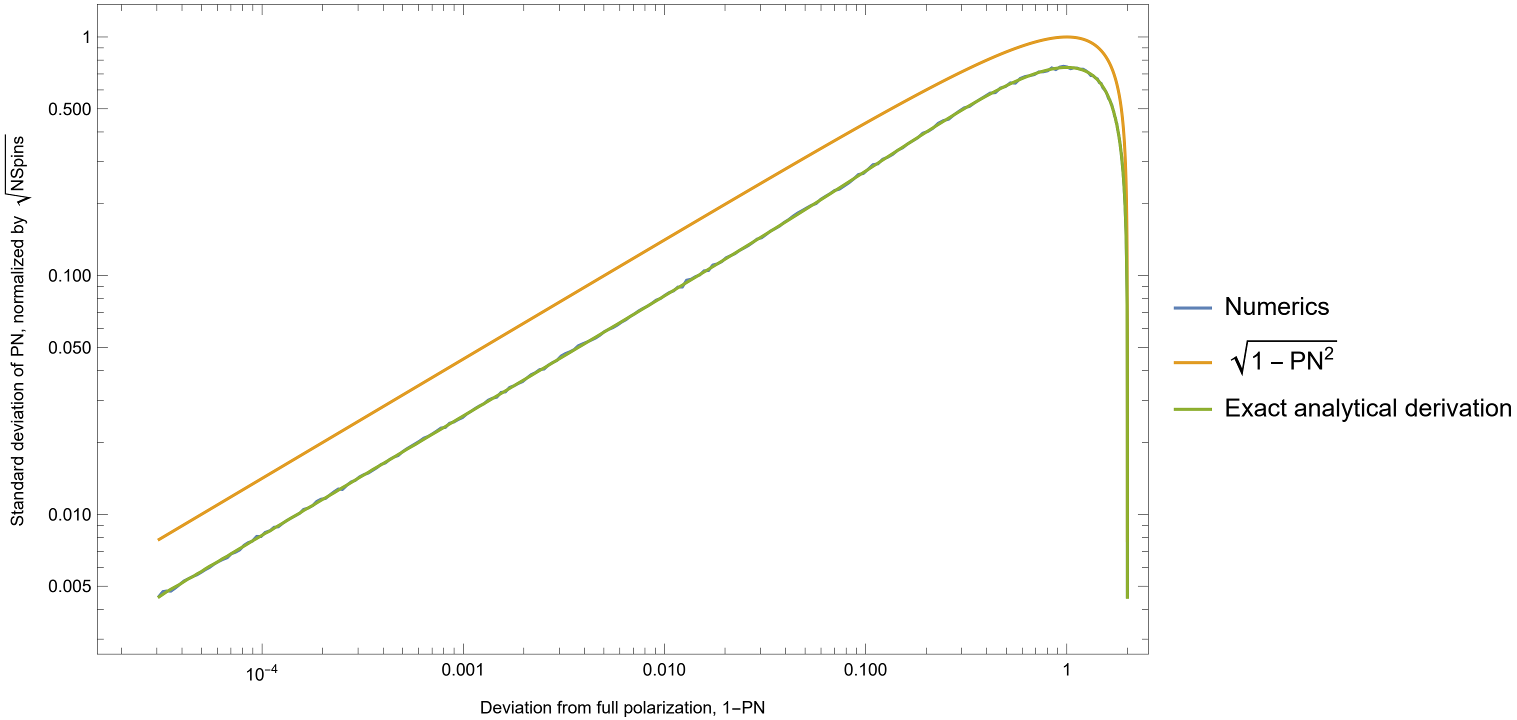

Summary of the results:

- Very high polarization degree PN~99.99% is required to get a factor of ~100 reduction in polarization fluctuations.
- The exact analytical equation gives a very good match (within ~ ±0.01) to the numerical result. (The larger the number of the random samples, the better the match.)
- Standard deviation of the nuclear polarization doesn't follow the $\sqrt{1 - PN^2}$ dependence. In the limit of high polarization degrees the exact variance follows $\sqrt{1 - PN^2}$ up to a constant factor on the order of ~1/2.
